# Characterization of a novel *Helitron* family in insect genomes: insights into classification, evolution and horizontal transfer

**DOI:** 10.1186/s13100-019-0165-4

**Published:** 2019-05-31

**Authors:** Guangjie Han, Nan Zhang, Jian Xu, Heng Jiang, Caihong Ji, Ze Zhang, Qisheng Song, David Stanley, Jichao Fang, Jianjun Wang

**Affiliations:** 1 College of Horticulture and Plant Protection, Yangzhou University, Yangzhou, 225009 China; 2 Jiangsu Lixiahe Institute of Agricultural Sciences, Yangzhou, 225007 China; 3 School of Life Sciences, Chongqing University, Chongqing, 400044 China; 4 Division of Plant Sciences, University of Missouri, Columbia, MO USA; 5 USDA/Agricultural Research Service, Biological Control of Insects Research Laboratory, Columbia, MO USA; 6 Institute of Plant Protection, Jiangsu Academy of Agricultural Sciences, Nanjing, 210014 China

**Keywords:** *Helitron*, Transposable elements, Horizontal transfer, Insects, Genome evolution

## Abstract

**Background:**

*Helitrons* play an important role in shaping eukaryotic genomes due to their ability to transfer horizontally between distantly related species and capture gene fragments during the transposition. However, the mechanisms of horizontal transfer (HT) and the process of gene fragment capturing of *Helitrons* still remain to be further clarified.

**Results:**

Here, we characterized a novel *Helitron* family discontinuously distributed in 27 out of 256 insect genomes. The most prominent characteristic of *Hel1* family is its high sequence similarity among species of different insect orders. Related elements were also identified in two spiders, representing the first report of spider *Helitrons*. All these elements were classified into 2 families, 9 subfamilies and 35 exemplars based on our new classification criteria. Autonomous partners of *Helitron* were reconstructed in the genomes of three insects and one spider*.* Integration pattern analysis showed that majority of *Hel1A* elements in *Papilio xuthus* and *Pieris rapae* inserted into introns. Consistent with filler DNA model, stepwise sequence acquisition was observed in *Sfru_Hel1Aa*, *Sfru_Hel1Ab* and *Sfru_Hel1Ac* in *Spodoptera frugiperda*. Remarkably, the evidence that *Prap_Hel1Aa* in a Lepdidoptera insect, *Pieris rapae*, was derived from *Cves_Hel1Aa* in a parasitoid wasp, *Cotesia vestalis*, suggested the role of nonregular host-parasite interactions in HT of *Helitrons*.

**Conclusions:**

We proposed a modified classification criteria of *Helitrons* based on the important role of the 5′-end of *Helitrons* in transposition, and provided evidence for stepwise sequence acquisition and recurrent HT of a novel *Helitron* family. Our findings of the nonregular host-parasite interactions may be more conducive to the HT of transposons.

**Electronic supplementary material:**

The online version of this article (10.1186/s13100-019-0165-4) contains supplementary material, which is available to authorized users.

## Introduction

As the single largest component of the genetic material of most eukaryotic and proeukaryotic species, transposable elements (TEs) play key roles in the epigenetic regulation of the genome and generation of genomic novelty [1, 2]. Depending on the mode of transposition, TEs are traditionally categorized as class-I elements or retrotransposons and class-II elements or DNA transposons [1, 3]. Copy and paste retrotransposons replicate via reverse transcription of an RNA intermediate of a source element, and can be further divided into long terminal repeat (LTR) and non-LTR retrotransposons. DNA transposons move through a single or double-stranded DNA intermediate, and are classified into three major subclasses, including the classic “cut-and-paste” transposons, rolling-circle (RC) transposons called *Helitrons*, and self-synthesizing transposons called *Mavericks/Polintons.* Both retrotransposons and DNA transposons exist as self-mobilizing autonomous elements or non-autonomous elements relying on trans-mobilization by the enzymatic machinery of their autonomous counterparts [4].

*Helitrons*, a novel superfamily of transposons, were originally discovered by *in silico* genome-sequence analysis [5], and later identified in a wide range of organisms, from protists to mammals [6, 7]. *Helitrons* are fundamentally different from classical transposons in terms of enzymatic activity and structure. *Helitrons* encode a RepHel protein homologous to RCR prokaryotic transposases, which comprises the replication initiator (Rep) and helicase (Hel) domains and is predicted to have both HUH (His-hydrophobe-His) endonuclease activity and 5′ to 3′ helicase activity [8]. *Helitrons* do not create target site duplications or contain terminal inverted repeats, and recent studies show that they transpose via copy-and-paste rather than cut-and-paste mechanism [9]. The characteristic features of *Helitrons* include a ‘TC’ motif on the 5′-end and a ‘CTRR’ motif on the 3′-end, and a palindromic sequence of 16–20 bp near the 3′-end, which can form a hairpin structure. Because of the minimal sequence feature and high sequence heterogeneity among *Helitron* copies, a classification system for family and subfamily definition has been proposed based on genome-wide analysis of *Helitrons* in the maize, *Zea mays* [10].

*Helitrons* have attracted widespread attention because their remarkable ability to capture gene fragments at the DNA level makes them play an important role in the host genome evolution. This process appears to have been particularly remarkable in the maize genome, where it is estimated that at least 20,000 gene fragments have been picked up and shuffled by *Helitrons* [10–12]. High frequency of *Helitron*-mediated gene capture is also reported in bats [13]. A recent study revealed that *Helitrons* have captured 3724 fragments from 268 genes in the silkworm, *Bombyx mori* [14]*.* Several models have been proposed to explain the mechanism of gene capture at the DNA level including end bypass and filler DNA model [8, 15].

Horizontal transfer (HT) is the non-vertical exchange of genetic material between reproductively isolated species. The inherent mobility and replication abilities of TEs facilitate them to undergo vector-mediated HT between organisms to avoid co-evolved host suppression mechanisms leading to vertical inactivation [1, 16–18]. The first evidence for the repeated HT of four different families of *Helitrons* including *Heligloria*, *Helisimi*, *Heliminu*, and *Helianu,* was described in an unprecedented array of organisms, including mammals, reptiles, fish, invertebrates, and polydnaviruses [19]. Subsequent identification of horizontally transferred *Helitrons*, such as *Hel-2* [20], *Lep1* [21], suggesting that *Helitrons* rely heavily on HT for their propagation and maintenance throughout evolution [21]. However, the physiological or ecological factors favoring the high frequency of HT still remains elusive.

Here, we have conducted a thorough search for the distribution of a novel *Helitron* family by analyzing the sequenced genomes of 256 insects and 22 spiders. We found that *Hel1* elements distributed in 27 investigated insect genomes as well as the genome of a distantly related spider, *Nephila clavipes*, which were classified into 9 subfamilies and 34 exemplars. A related *Hel2* family was identified in the genome of a spider, *Parasteatoda tepidariorum*. Furthermore, we provided evidence for stepwise sequence acquisition and recurrent HT of this novel *Helitron* family. Our results provided new insights into the classification and evolution of *Helitrons*, and suggested that the *Helitrons* can undergo horizontal transfer by diverse means.

## Results

### Identification and distribution of a novel *Helitron* transposon

A novel *Helitron* element was occasionally found during the analysis of flanking sequence of one short interspersed nuclear element (*SINE*) in *Chilo suppressalis* (Additional file 1: Figure S1). Subsequent database search detected a total of 884 similar sequences in *C. suppressalis* genome (Additional file 2: Table S1). Sequence analysis showed that these sequences present the typical structural features of the *Helitron* transposons: almost all copies have characteristic 5′-TC and 3′-CTRY nucleotide termini. The integration occurs precisely between the host A and T nucleotides, without duplications or deletions of the target sites, consistent with the RC mechanism (Additional file 1: Figure S2a). Further analysis showed that these *Helitron* sequences could be divided into three exemplars of two subfamilies, *Csup_Hel1Aa*, *Csup_Hel1Ab* and *Csup_Hel1Ea*. Their consensus sequences are of 162, 257 and 195 bp long, respectively. The conserved 3′-stem-loops (hairpins) were also predicted upstream of the 3′-CTRR termini of *Csup_Hel1A* exemplar (Additional file 1: Figure S2b).

**Fig. 1:**
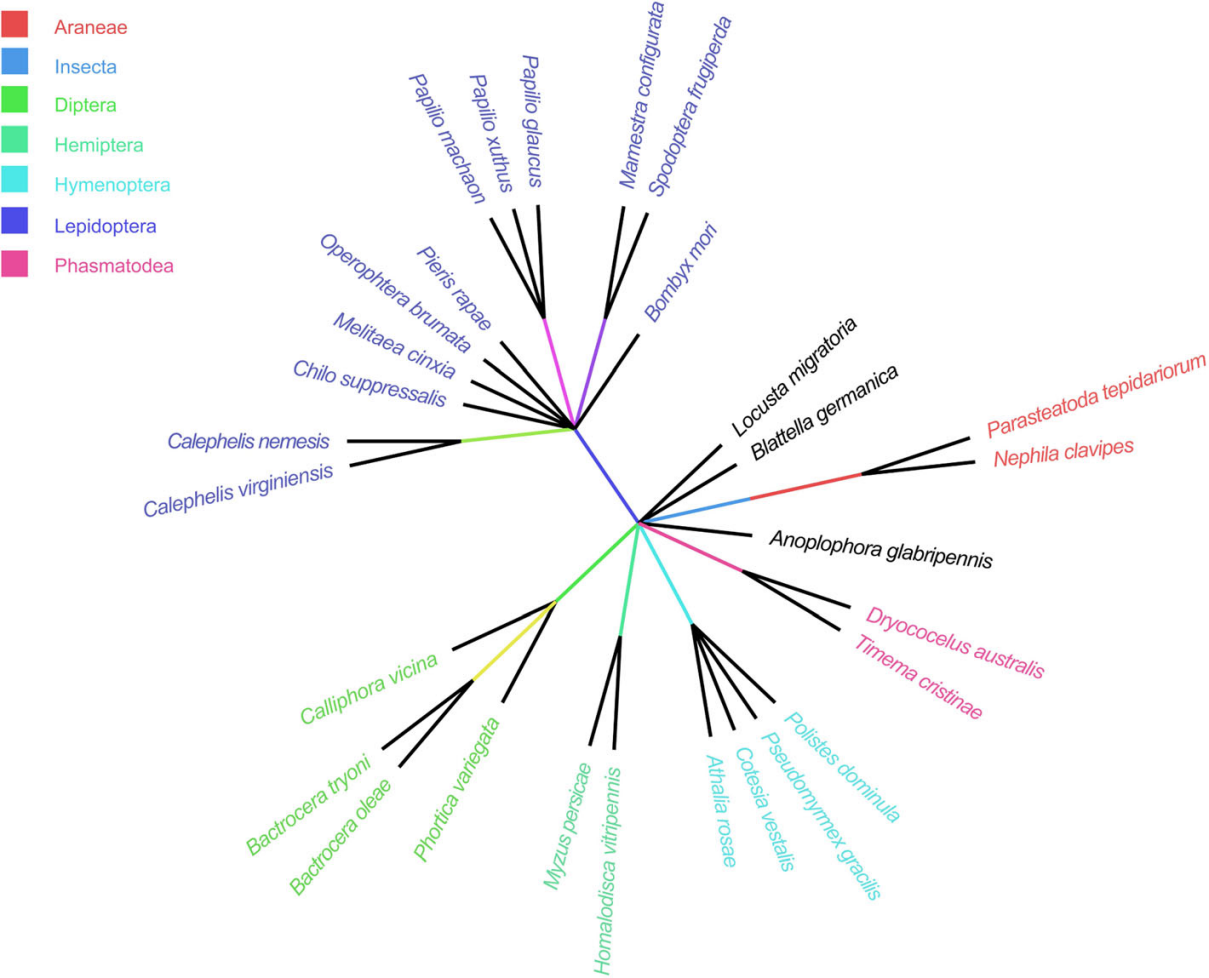
The taxonomy tree of 29 species containing *Csup_Hel1A*-like *Helitrons*

**Fig. 2:**
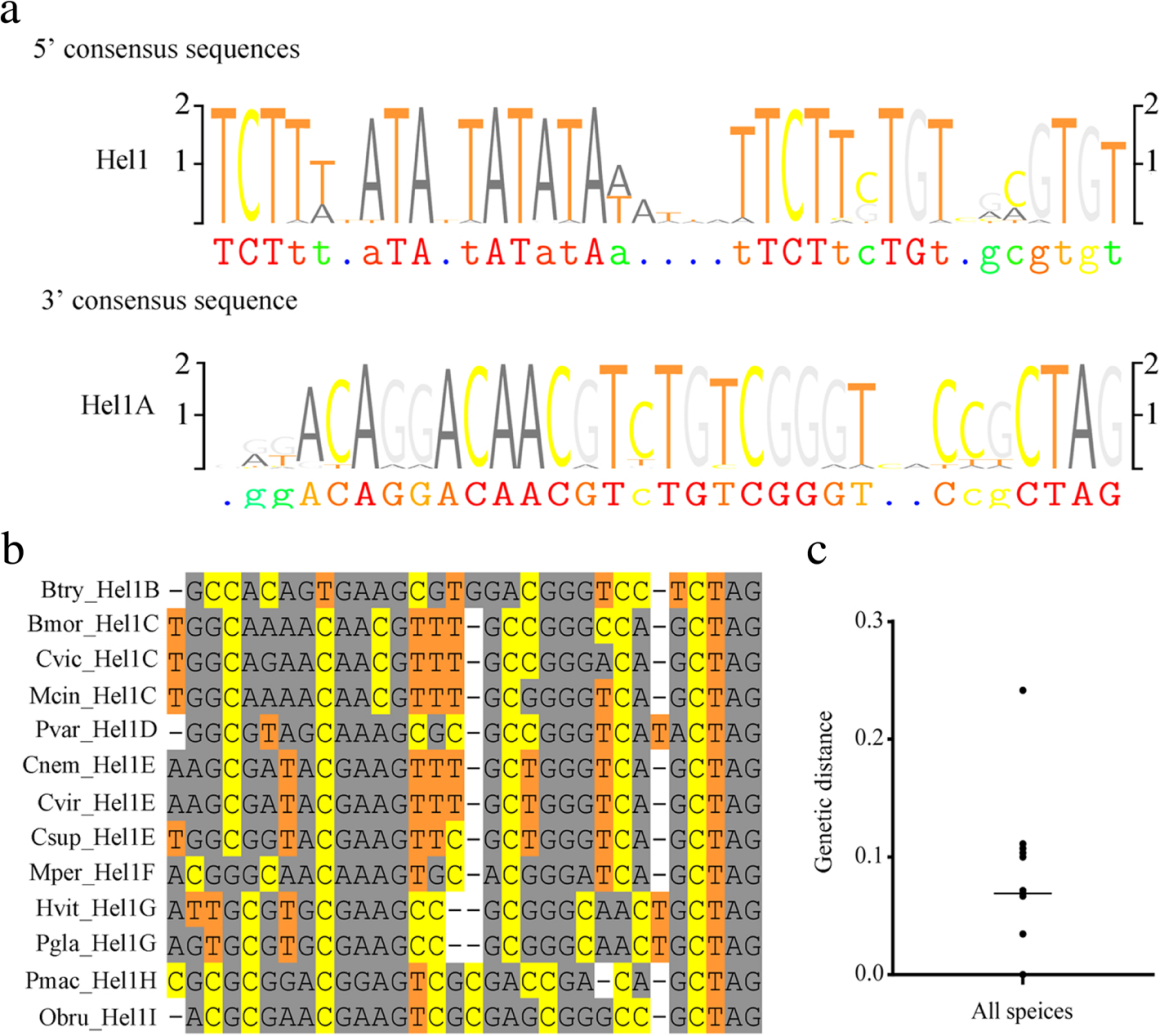
Multiple alignment of 34 *Hel1* exemplars. **a** Multiple alignment of 30 bp region at the 5′-ends from *Hel1* families and 30-bp region at the 3′-ends from *Hel1A* subfamilies. The alignment was graphically edited using TeXshade package. **b** Multiple alignment of 30 bp region at the 3′-ends from *Hel1B*-*Hel1I* subfamilies. **c** Genetic distance analysis of *Hel1* and *Hel2* from all species, the average distance is 0.069

A broad homology-based search of contemporary whole genome shotgun (WGS) databases of 256 insects identified similar elements in 27 species, including 12 out of 42 Lepidoptera, 4 out of 110 Diptera, 4 out of 52 Hymenoptera, 1 out of 2 Orthoptera, 2 out of 2 Phasmatodea, 1 out of 12 Coleoptera, 2 out of 23 Hemiptera, 1 out of 3 Blattodea. Among 22 spider WGS genome database, similar elements were only found in *P. tepidariorum* and *N. clavipes* (Additional file 1: Figure S3 and Fig. 1). These elements vary in size from 162 bp to 8065 bp. The number of copies also varies from exemplar to exemplar. *Cvir_Hel1Ea* in *Calephelis virginiensis* showed the highest copy numbers of 5578, occupies 0.479% of genome, while only 4 copies were found in *Aros_Hel1Aa* in *Athalia rosae.* The average percentage divergence varied from 0.01 to 15.672, indicating different invasion time (Table 1).

**Fig. 3:**
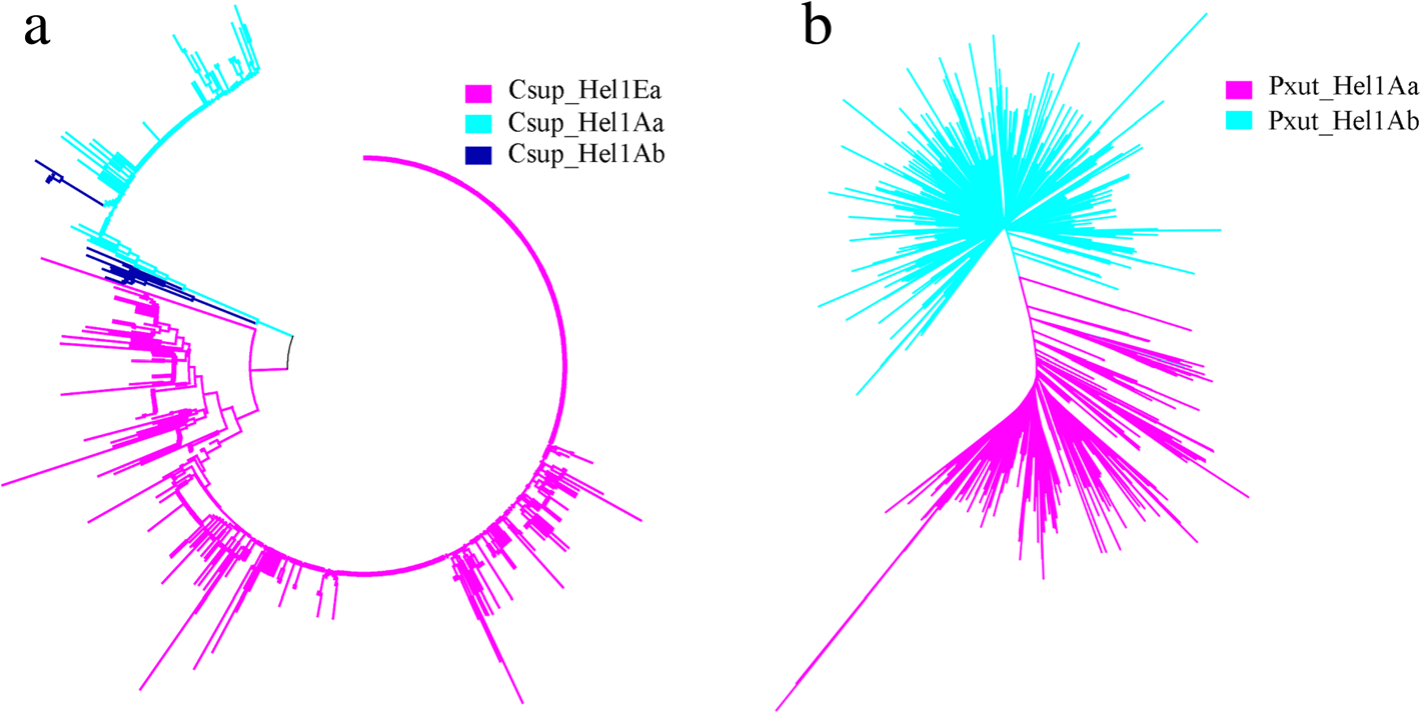
Phylogenetic analysis of all copies of *Hel1 Helitrons* in *C. suppressalis* (**a**) and *P. xuthus* (**b**). Copies of different exemplars are indicated by distinct colors. The phylogenetic tree was constructed by the neighbor-joining method using MEGA 7.0 software

**Table 1:** Characteristics of 35 *Helitron* exemplars from 29 species

Group		Average divergence^a^	Length (bp)^b^	Copies (% Genome)
Araneae (2/5)				
*Parasteatoda tepidariorum*	*Ptep_Hel2Aa*	0.322	2903 (11)	345 (0.069)
*Nephila clavipes*	*Ncla_Hel1Aa*	0.532	293 (79)	4611 (0.055)
Blattaria (1/3)				
*Blattella germanica*	*Bger_Hel1Aa*	ND	664 (5)	91 (0.003)
Orthoptera (1/2)				
*Locusta migratoria*	*Lmig_Hel1Aa*	0.319	2080 (3)	86 (0.003)
Hemiptera (2/23)				
*Myzus persicae*	*Mper_Hel1Fa*	1.278	551 (30)	420 (0.067)
*Homalodisca vitripennis*	*Hvit_Hel1Ga*	1.024	930 (165)	1914 (0.081)
Coleoptera (1/12)				
*Anoplophora glabripennis*	*Agla_Hel1Aa*	0.740	195 (17)	27 (0.001)
Lepidoptera (12/42)				
*Bombyx mori*	*Bmor_Hel1*			702
	*Bmor_Hel1Aa*	15.672	249 (4)	8 (0.000)
	*Bmor_Hel1Ca*	3.004	217 (67)	680 (0.037)
*Spodoptera frugiperda*	*Sfru_Hel1*			3404
	*Sfru_Hel1Aa*	0.852	196 (60)	495 (0.022)
	*Sfru_Hel1Ab*	0.164	262 (86)	463 (0.028)
	*Sfru_Hel1Ac*	0.016	378 (69)	1113 (0.096)
*Mamestra configurata*	*Mcon_Hel1Aa*	0.663	223 (63)	1814 (0.071)
*Melitaea cinxia*	*Mcin_Hel1Ca*	0.132	356 (62)	271 (0.025)
*Pieris rapae*	*Prap_Hel1Aa*	0.054	447 (449)	1520 (0.276)
*Papilio glaucus*	*Pgla_Hel1Ga*	0.467	165 (35)	461 (0.020)
*Papilio xuthus*	*Pxut_Hel1*			2326
	*Pxut_Hel1Aa*	0.069	296 (56)	796 (0.097)
	*Pxut_Hel1Ab*	0.066	204 (153)	722 (0.060)
*Papilio machaon*	*Pmac_Hel1Ha*	0.015	178 (20)	39 (0.002)
*Operophtera brumata*	*Obru_Hel1Ka*	12.134	193 (30)	437 (0.013)
*Chilo suppressalis*	*Csup_Hel1*			1760
	*Csup_Hel1Aa*	0.010	162 (95)	103 (0.005)
	*Csup_Hel1Ab*	0.018	257 (16)	95 (0.008)
	*Csup_Hel1Ea*	0.034	195 (86)	752 (0.047)
*Calephelis nemesis*	*Cnem_Hel1Ea*	0.919	271 (62)	5578 (0.481)
*Calephelis virginiensis*	*Cvir_Hel1Ea*	2.171	269 (158)	2703 (0.231)
Diptera (4/110)				
*Bactrocera tryoni*	*Btry_Hel1Ba*	0.207	1291 (4)	1325 (0.330)
*Bactrocera oleae*	*Bole_Hel1Aa*	0.302	1206 (4)	186 (0.056)
*Phortica variegata*	*Pvar_Hel1Da*	0.205	343 (5)	38 (0.008)
*Calliphora vicina*	*Cvic_Hel1Ca*	0.436	264 (52)	214 (0.012)
Hymenoptera (4/52)				
*Pseudomyrmex gracilis*	*Pgra_Hel1Aa*	ND	748 (5)	83 (0.022)
*Athalia rosae*	*Aros_Hel1Aa*	ND	8065 (1)	4 (0.020)
*Cotesia vestalis*	*Cves_Hel1Aa*	0.372	278 (76)	137 (0.020)
*Polistes dominula*	*Pdom_Hel1Ia*	0.968	395 (3)	165 (0.031)
Phasmatodea (2/2)				
*Dryococelus australis*	*Daus_Hel1Aa*	0.077	667 (27)	1326 (0.026)
*Timema cristinae*	*Tcri_Hel1Aa*	0.099	3435 (5)	105 (0.035)

*ND*, not determined

^a^Average divergence is calculated between copies within a species

^b^The number in bracket is the number of copies used to reconstruct the consensus sequences

Multiple alignment of the consensus sequences showed that the 30 bp fragments at the 5′-end showed as high as 93.1% average identity, however, the 30 bp fragments at the 3′-end showed somewhat sequence divergence. Among 35 exemplars, 22 showed above 80% pairwise identities, the rest 13 exemplars showed less than 80% identities with above 22 exemplars (Fig. 2). The high sequence identity of 5′-end extended to 126 bp (89% identity) except *Ptep_Hel2Ca* (Additional file 2: Table S2 and Additional file 1: Figure S4). According to our novel classification method, these elements were divided into 2 families, *Hel1* and *Hel2*, and 9 subfamilies, *Hel1A*-*Hel1I*. While the 30 bp fragment at the 3′-end of *Ptep_Hel2Ca* showed over 80% identity with *Hel1C*, as low as 73.3% identity was found between 30 bp fragment at the 5′-end of *Ptep_Hel2Ca* and *Csup_Hel1A* (Additional file 1: Figure S5). Different exemplars were found in the same species. For example, in the genome of *C. suppressalis*, three exemplars of two subfamilies, *Csup_Hel1Aa*, *Csup_Hel1Ab*, and *Csup_Hel1Eb*, were detected, which showed average percentage divergence of 0.01, 0.018 and 0.034, respectively (Table 1). The classification was supported by evolutionary analysis, which showed that these three exemplars are polyphyletic in origin and separated into three distinct clades. Similarly, two exemplars of *Helitron* subfamily, *Pxut_Hel1Aa* and *Pxut_Hel1Ab* were found in the genome of *Papilio xuthus* with the average percentage divergence of 0.064 and 0.066, respectively, and cluster into two distinct clades (Fig. 3).

**Fig. 4:**
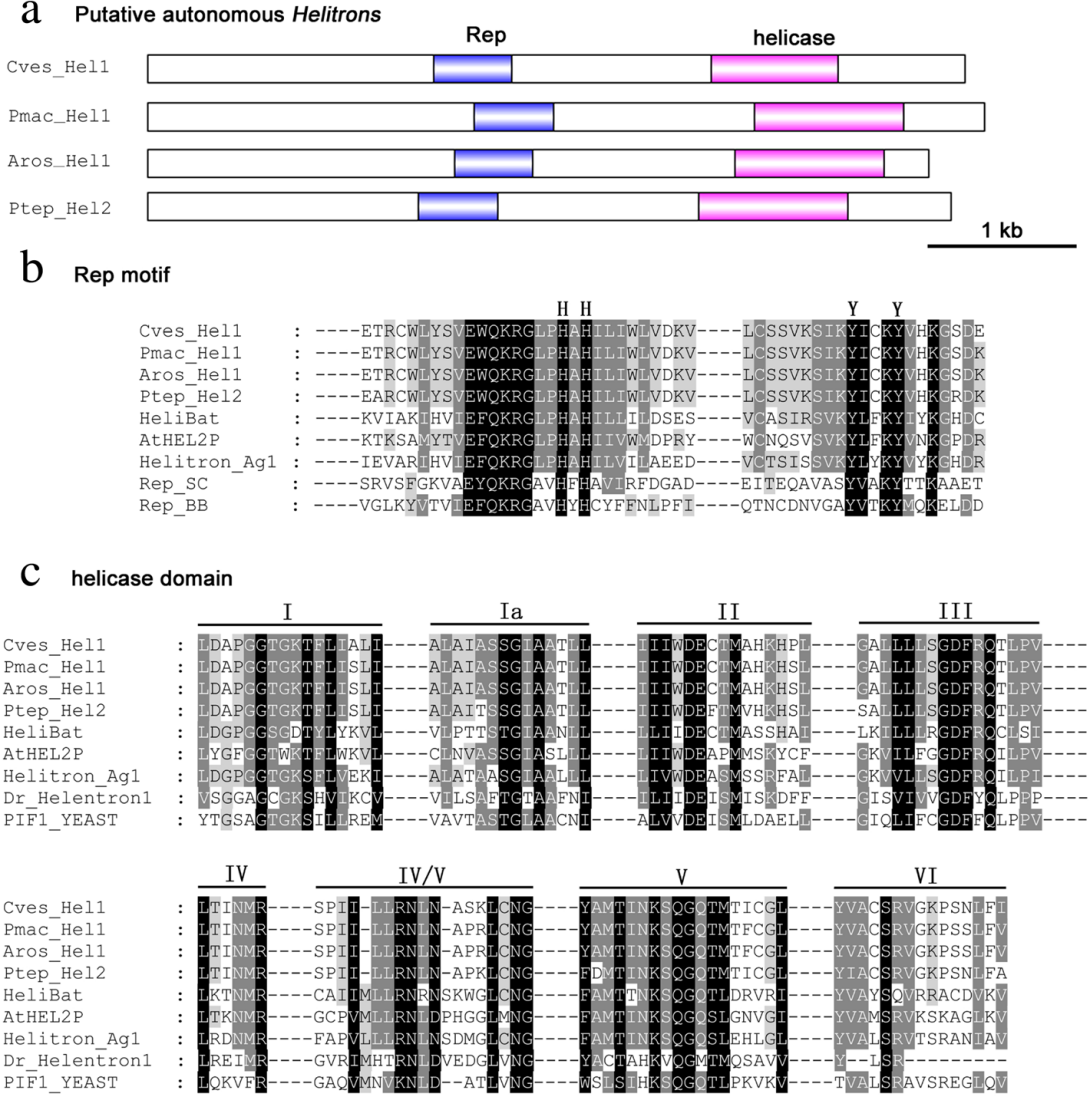
Gene structure (**a**), predicted Rep motif (**b**) and helicase domain (**c**) of reconstructed putative autonomous *Hel1* and *Hel2* Helitrons from *C. vestalis, P. machaon*, *A. rosae* and *P. tepidariorum*. In the alignment of the Rep motif, representative structure was from *Myotis lucifugus* (HeliBat1), *Arabidopsis thaliana* (AtHEL2P), *Anopheles gambiae* (Helitron_Ag1), S*treptomyces cyaneus* plasmid (Rep_SC) and *Bacillus borstelensis* plasmid (Re_BB). In the alignment of helicase domain, representative structure was from *Myotis lucifugus* (HeliBat1), *Arabidopsis thaliana* (AtHEL2P), *Anopheles gambiae* (Helitron_Ag1), *Danio rerio* (Dr_Helentron1) and *Saccharomyces cerevisiae* (PIF1_YEAST)

**Fig. 5:**
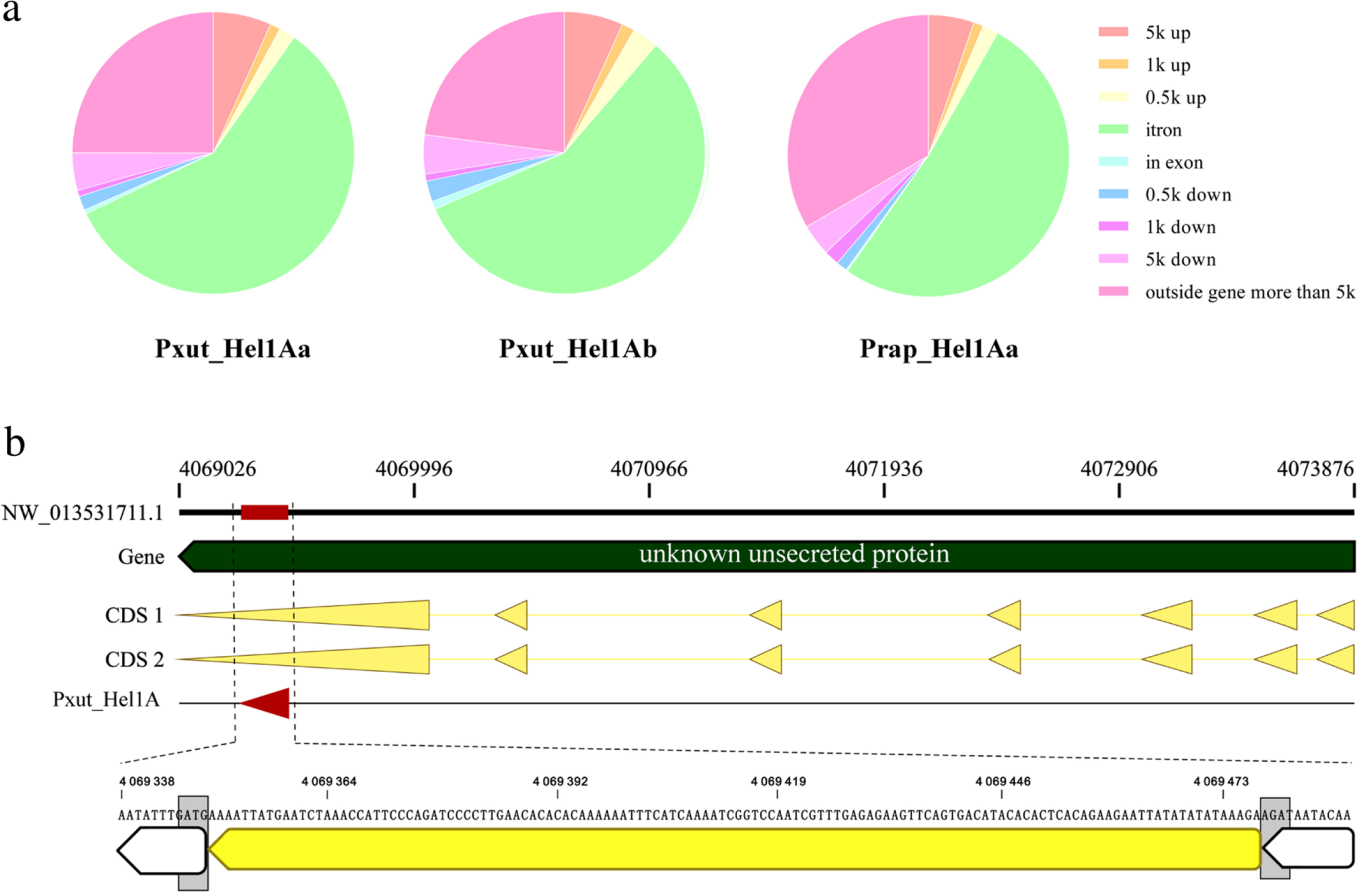
Gene association of *Hel1A Helitrons* in *P. xuthus* and *P. rapae*. **a** Overall proportions of *Pxut_Hel1Aa*, *Pxut_Hel1Ab* and *Prap_Hel1Aa* in the respective genome are represented as pie charts. **b** Integration of a *Pxut_Hel1A* element within the coding sequence (CDS) of a gene encoding an unclassified protein in *P. rapae*

### Characterization of reconstructed potential autonomous DNA *Helitrons*

A total of 7 *Helitrons* with degenerated remnants of *Helitron* coding sequences were initially detected in the genome of *N. clavipes*, *P. tepidariorum*, *Papilio machaon*, *Cotesia vestalis*, *Homalodisca vitripennis*, *A. rosae* and *Timema cristinae* (Additional file 3: Data S1)*.* The longest *Helitron* was found in *A. rosa*, with the length of 8065 bp, and at position 785–1860, there was an insertion of 1076 bp fragment putatively encoding C-terminal catalytic domain of Cre recombinase (INT_Cre_C). Due to the short sequencing length of WGS sequence, the characteristic 5′-TC nucleotide termini was not found in *P. machaon*, however, 2 copies of 247 bp tandem repetitive sequences (TRS) were detected at 5′-end of this contig (Additional file 1: Figure S6).

**Fig. 6:**
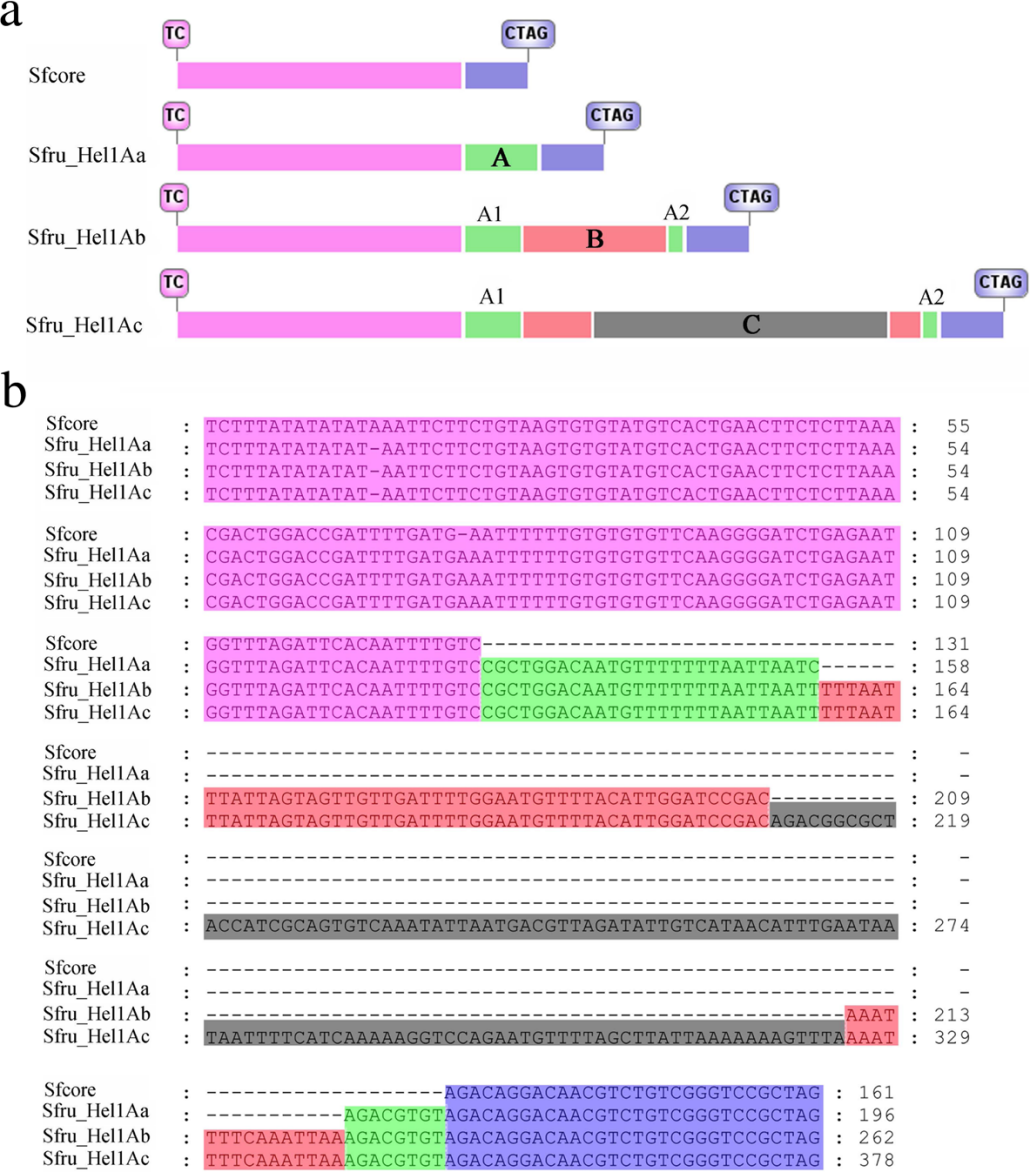
Stepwise transposition of *Sfru_Hel1A* in *S. frugiperda*. **a** Schematic structures of core sequence and exemplars in *S. frugiperda*. The 131-bp 5′-end and 30-bp 3′-end sequences are colored in pink and blue, respectively. The A, B and C regions are in green, red and black, respectively. Typical structural features of *Helitron* elements including characteristic 5′-TC and 3′-CTRY nucleotide termini were boxed. **b** Multiple alignment of core sequence and the consensus sequences of *Hel1* exemplars in *S. frugiperda*

Further blast searches were executed and 4 potential autonomous *Helitrons* with an uninterrupted ORF coding for Rep/helicase of 1495, 1467, 1495 and 1496 amino acids were reconstructed in *C. vestalis* (Cves_Hel1), *P. tepidariorum* (Ptep_Hel2)*, P. machaon* (Pmac_Hel1) and *A. rosae* (Aros_Hel1), respectively (Additional file 3: Data S2). Multiple alignment showed that the predicted Rep/helicase proteins are composed of a Rep domain containing “two-His” replication initiator motifs and two conserved tyrosine residues, and a helicase domain containing eight conserved motifs of the SF1 superfamily of DNA helicases [22] (Fig. 4). More than 86% amino acid identities were observed among these 4 Rep/helicase proteins. Specially, the amino acid identity of *Helitrons* in *P. machcaon* and *A. rosae* is more than 98% (Additional file 1: Figure S7).

**Fig. 7:**
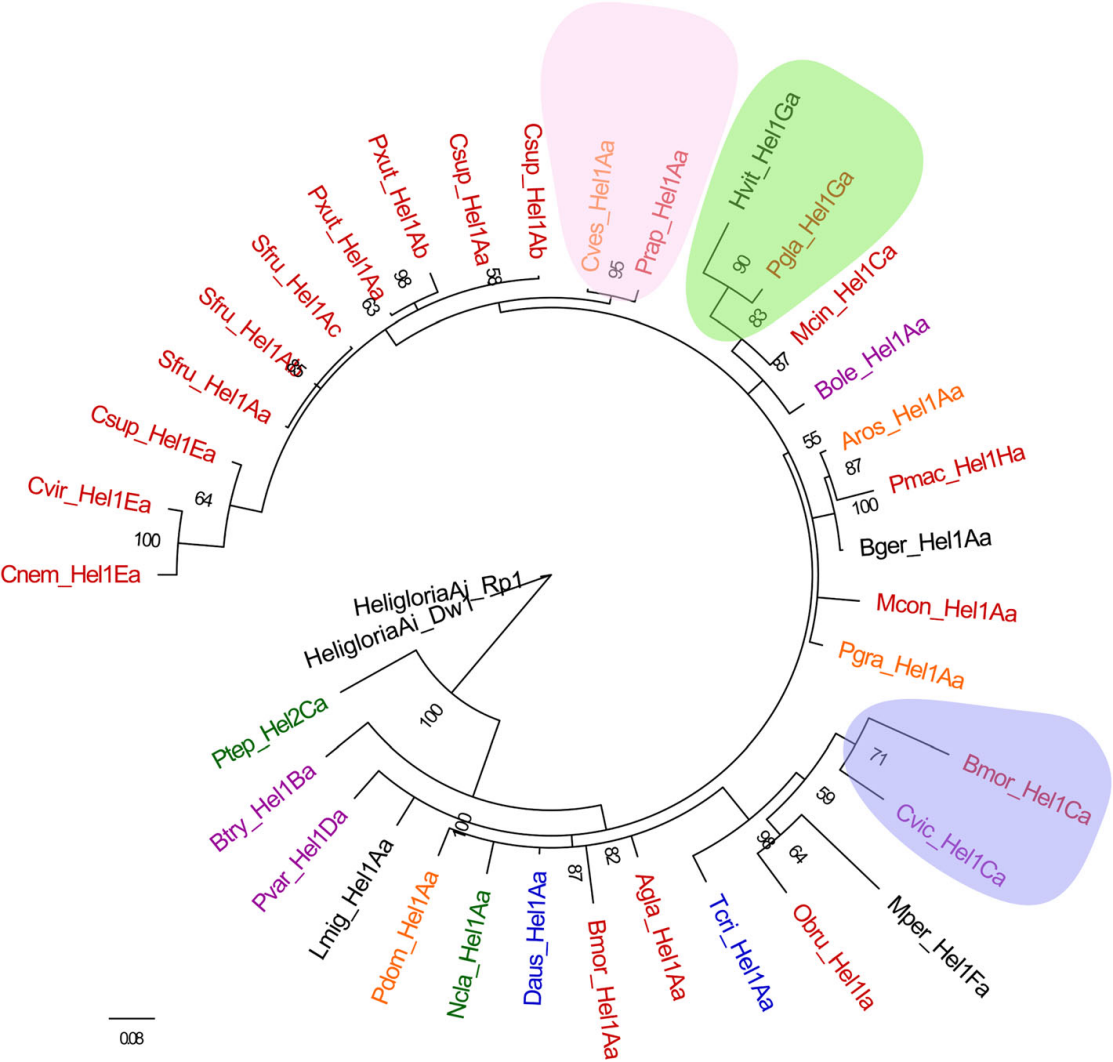
Phylogenetic relationships among 35 exemplars from 29 species. Taxa showing *Hel1* and *Hel2* are colored taxonomically, with Lepidopteran insects in red, Diptera insects in purple, Hymenoptera wasps in yellow, Phasmatodea species in blue, Araneae species in green. The opaque projection indicates the HT between the two species

### Contribution of *Hel1* to gene and genome evolution

We further analyzed the integration pattern relative to the annotated genes in two representative genomes, *P. xuthus* and *Pieris rapae*. Out of the 796, 722 and 1520 copies of *Pxut_Hel1Aa*, *Pxut_Hel1Ab* and *Prap_Hel1Aa*, 463 (58%) of *Pxut_Hel1Aa Helitrons*, 413 (57%) of *Pxut_Hel1Ab Helitrons*, and 740 (52%) of *Prap_Hel1Aa Helitrons* were found in introns. Only 4, 7 and 3 copies of *Pxut_Hel1Aa*, *Pxut_Hel1Ab* and *Prap_Hel1Aa* were found to insert into exons, respectively (Fig. 5a). Further analysis revealed the insertion of multiple copies of *Hel1* into introns of the same gene. For example, as many as 4 copies of *Prap_Hel1Aa* inserted into introns of LOC110995424 gene, and the fifth copy inserted into 3′-end of coding sequence (CDS) (Additional file 1: Figure S8). However, in most cases, only one copy was detected in intron regions of a specific gene. Notably, a 127 bp copy of *Pxut_Hel1A* (NW_013531711.1: 4069476–4,069,349) inserted into CDS of a gene encoding an unclassified protein (Fig. 5b). Thus, the *P. xuthus* and *P. rapae Hel1 Helitrons* mainly contribute to structural variation in introns, which might influence the regulation of gene expression.

### Sequence acquisition and new *Hel1* creation

Among all *Helitrons* identified in this study, *Hel1* in *Spodoptera frugiperda* attracted our attention. In addition to *Sfru_Hel1Aa*、*Sfru_Hel1Ab* and *Sfru_Hel1Ac*, a 161 bp copy (FJUZ01003913.1: 464817–464,657) was detected (Fig. 6). Sequence analysis showed that the consensus sequences of these three exemplars shared almost 99% identity with this short sequence excluding insertions, thus this short copy was designated as core sequence (Sfcore) (Fig. 6). Further analysis showed that compared with the core sequence, a 35 bp fragment named “A” region inserted into core sequence 130 bp downstream of the 5′-end in *Sfru_Hel1Aa*, and a 66 bp fragment named “B” region inserted into “A” region in *Sfru_Hel1Ab*, while a 108 bp fragment named “C” region inserted into “B” region in *Sfru_Hel1Ac* (Fig. 6a). Alignment of the consensus sequences showed that the insertion sites were consistent with the overlapping region (Fig. 6b). The search of putative source loci of these insertions revealed that “A” region consisted of “A1” and “A2” regions, among which “A1” region was derived from sequence NJHR01000652 (137291–137,357) and “A2” region from NJHR01000244 (961324–961,247) (Additional file 1: Figure S9). While sequence NJHR01000585 (153969–153,870) showed high identity with “B” region, we did not find source locus of “C” region, putative due to the incomplete genome sequencing. Additionally, 2-13 bp end junctions were identified in each source locus, supporting the filler DNA model [23] (Additional file 1: Figure S9). Furthermore, the average percentage divergence was 0.852, 0.164 and 0.016, respectively, indicating a clear evolutionary order (Table 1).

The sequence acquisition of *P. xuthus Hel1* is different from that of *S. frugiperda*. Two exemplars of *Hel1A* subfamily, *Pxut_Hel1Aa* and *Pxut_Hel1Ab*, were found in *P. xuthus*, with the length of 296 bp and 204 bp, respectively, and only 172 bp region was shared by these two exemplars (Additional file 1: Figure S10a). The average percentage divergence of *Pxut_Hel1Aa* and *Pxut_Hel1Ab* was 0.069 and 0.066, respectively (Table 1). It seems unlikely that *Pxut_Hel1Ab* was formed by the sequence acquisition of *Pxut_Hel1Aa*. Furthermore, we also found a core sequence (BBJE01004687.1: 58267–58,430) highly similar to that of *S. frugiperda* (Additional file 1: Figure S10b)*.* We speculated that *Pxut_Hel1Aa* and *Pxut_Hel1Ab* were independently derived from the core sequence by sequence acquisition during transposition.

In case of *C. suppressalis*, the 162 bp consensus sequence of *Csup_Hel1Aa* was over 96% identical to the above core sequences of *P. xuthus* and *S. frugiperda* (Additional file 1: Figure S10b). Compared with *Csup_Hel1Ab*, a 7 bp fragment (AGACGTG) was unique to *Csup_Hel1Aa* (Additional file 1: Figure S10b). Given similar average percentage divergence in these two exemplars, it seems that *Csup_Hel1Ab* was not derived from *Csup_Hel1Aa*. On the other hand, 5 core sequences with high similarity to *Csup_Hel1Ea* were also found in the genome of *C. suppressalis* (Additional file 1: Figure S10c). Considering that the average percentage divergence of *Csup_Hel1Ea* was 0.034, we inferred that *Csup_Hel1Ea* was evolutionarily earlier than *Csup_Hel1A*, and had different origin with *Csup_Hel1Aa*.

### Evolution and horizontal transfer of *Hel1*

Using *HeligloriaAi_DW1* and *HeligloriaAi_Rp1* as out group [19], the phylogenetic tree of the 35 *Helitron* consensus sequences showed that *Ptep_Hel2Ca* was evolutionarily different from other *Hel1* elements, and insects of the same order were not clustered together. The incongruence of *Hel1* elements and host phylogeny as well as the patchy distribution and high sequence similarity of *Hel1* elements among distantly related lineages suggest the recurrence of HT and that multiple mechanisms may underlie the horizontal spread of *Hel1*. Notably, Lepidopteran *Prap_Hel1Aa* and Hymenopteran *Cves_Hel1Aa*, Dipteran *Cvic_Hel1Ca* and Lepidopteran *Bmor_Hel1Ca*, Hemipteran *Hvit_Hel1Ga* and Lepidopteran *Pgla_Hel1Ga* were clustered into distinct clades, which diverged 325, 272 and 358 million years ago, respectively (http://www.timetree.org/) [24] (Fig. 7). Furthermore, several paralogous and orthologous empty sites were also detected in these insect genomes (Additional file 1: Figure S11). It is also noteworthy that the genetic distance between species of the same cluster was less than 0.1, indicating that these elements have spread horizontally among these species within a relatively narrow timeframe.

The clustering of *Prap_Hel1Aa* from *P. rapae* (Lepidoptera: Pieridae) and *Cves_Hel1Aa* from *C. vestalis* (Hymenoptera: Braconidae) into the same clade is of particular interest. While the calculated genetic distances of orthologous genes *calreticulin*, *Hsc70* and *opsin* between *P. rapae and C. vestalis* were 0.325, 0.229 and 0.312, respectively (Additional file 1: Figure S12a, b, c), sequence comparison showed that the consensus sequences of *Prap_Hel1Aa* and *Cves_Hel1Aa* shared over 98% identity excluding a 169 bp insertion in *Prap_Hel1Aa* (Additional file 1: Figure S12d)*.* Considering the average percentage divergence of *Prap_Hel1Aa* and *Cves_Hel1Aa* were 0.054 and 0.372, respectively, we speculated that *Prap_Hel1Aa* was derived from *C. vestalis* through HT, followed by the capture of 169 bp fragment and a rapid burst in transposition. This hypothesis was partly supported by the reconstructed phylogeny in which the *Cves_Hel1Aa* copies are generally nested within clades made of *Prap_Hel1Aa* copies, and the closely related *Csup_Hel1Aa* copies from *C. suppressalis* were phylogenetically separated from both *Prap_Hel1Aa* and *Cves_Hel1Aa* (Additional file 1: Figure S13). Additionally, the 169 bp insertion fragment was almost entirely absent in a short copy of *Prap_Hel1Aa* (Prap0202, LWME01000202.1: 138955–138,682), which was over 94% identical to eight copies of *Cves_Hel1Aa*, and specifically over 97% identity was observed at the 30 bp 3′-ends of Prap0202 and these *Cves_Hel1Aa* copies (Additional file 1: Figure S12e)*.* Interestingly, PCR amplification and sequencing revealed orthologous empty site of Prap0202 in a local population of *P. rapae*, suggesting *Prap_Hel1Aa* elements mobilized recently (Additional file 1: Figure S14). Furthermore, as many as 11 elements in *P. rapae* genome were found to be completely same as the consensus sequence of *Prap_Hel1Aa*, indicating recent invasion of the *P. rapae* genome by *Prap_Hel1Aa* elements (Additional file 1: Figure S15).

There are few reports on the occurrence of HT between Lepidoptera and Diptera [19]. In this study, we found that *Cvic_Hel1Ca* and *Bmor_Hel1Ca*, *Pgla_Hel1Ga* and *Hvit_Hel1Ga* were clustered into same clade, respectively, and the corresponding consensus sequences were highly similar (Additional file 1: Figure S16a and Figure S17), suggesting the occurrence of HT between these insects. Furthermore, orthologous empty sites were detected in both Lepidoptera and Diptera insects (Additional file 1: Figure S11). Notably, a putative *Hel1* sequence (GEND01024785.1: 446–129) was found in the transcriptome shotgun assembly (TSA) database of *Entomophthora muscae*, which shared 90% identity with two copies of *Cvic_Hel1Ca* and 74% identity with *Bmor_Hel1Ca* (Additional file 1: Figure S16b), suggesting a possible role of *E. muscae* in the HT between Lepidoptera and Diptera insects.

## Discussion

The classification of *Helitron* has always been ambiguous. The classical classification system was proposed based on genome-wide analysis of maize *Helitrons*, in which the sequences with the most similar 3′-ends (30 bp with at least 80% identity) were classified as members of the same family and sequences with the most similar 5′-ends (30 bp with at least 80% identity) were classified as members of the same subfamily [25]. This criteria has been followed by several other studies [19]. In addition, the unique internal sequence that was > 20% different at the nucleotide level from any other *Helitron* internal regions was defined “exemplars” [10]. However, based on genome-wide analysis of silkworm *Helitrons*, Han et al. (2013) suggested that sequences with identities > 80% in the 30 bp of both their 5′- and 3′-ends were classified as members of the same family, and full-length sequences with identity > 80% were classified in the same subfamily. Due to the lack of knowledge regarding *Helitron* cis- or trans- activation of *Helitron*, these classification criteria are exploratory. According to the end bypass model, which was proposed to explain the mechanism of gene capture of *Helitron*, transposition initiates at the 5′-end and gene capture occurs if the 3′-end signal is missed. A random cryptic sequence located downstream would then act as the termination signal and all intervening sequences would be captured [8, 26]. This model was supported by the fact that the *xanA* gene fragment was captured by a *Helitron* in *Aspergilus nidulans* genome [27]. Recent study showed that the modification or deletion of the hairpin loop or palindrome sequence had little effect on the transposon colony-forming activity of the reconstructed active bat *Helitron*, *Helraiser*. However, the deletion of 5′-end of *Heliraiser* resulted in complete loss of activity [9]. Given the importance role of the 5′-end of *Helitrons* in transposition, we think it seems more reasonable to classify the family with 5′-end of *Helitron*. Thus, we proposed a new classification standard, as described in methods. This new criteria was supported by our phylogenetic analysis of *P. xuthis* and *C. suppressalis Helitrons*, in which the copies of different subfamilies or exemplars of *Helitron* are well separated phylogenetically (Fig. 3).

The distinct copy and paste transposition process of *Helitrons* ensures them the capability of reaching high genomic copy numbers. For example, maize and silkworm *Helitrons* constitute 6.6% and 4.23% of the genome, respectively [11, 14]. In this study, as many as 5578 copies of *Cvir_Hel1Ea* were found in *C. virginiensis*, which account for 0.479% of genome. Besides their direct effect on genome size, evidence has accumulated in recent years that *Helitrons* can also impact the gene structure and expression as well as genome organization [28, 29]. For example, the insertion of two non-autonomous *Helitron* elements, *AtREP3* and *AtREP1*, into upstream of *ETT* and *ARF4* genes in *tebichi* (*teb*) mutant *Arabidopsis thaliana* resulted in the upregulation of these two genes [30]. In the tetraploid sour cherry, *Prunus cerasus*, the insertion of a small non-autonomous *Helitron* element into 38 bp downstream of the stop codon of *SFB* gene is proposed to interfere with the polyadenylation process, resulting in a loss of function of the *SFB* gene involved in gametophytic self-incompatibility [31]. In this study, we found that, similar to silkworm *Helitrons* [14], majority copies of *Pxut_Hel1A* and *Prap_Hel1Aa* insert into introns of host genome, suggesting that *Hel1* duplication and transposition led to structural variation in introns, which might influence the regulation of gene expression. Notably, a copy with 3′-end deletion of *Pxut_Hel1A* inserted into coding region of an unclassified gene (Gene accession: LOC110995424) in *P. xuthus* genome, while the impact of the insertion on gene function is unknown at present.

A predominant characteristic of *Helitrons* is their ability to capture and amplify host genome sequences. Among 1649 *Helitron-like* transposons identified in genome of maize inbred line B73, over 90% of maize *Helitrons* have captured gene fragments [32]. While end bypass and filler DNA models [8, 15] have been proposed to explain *Helitron* gene capture and transposition, the exact mechanisms is far from clear. It has been proposed that gene capture during *Helitron* transposition occurs in a stepwise or sequential way [33]. In this study, three exemplars of *Helitron*, *Sfru_Hel1Aa*, *Sfru_Hel1Ab* and *Sfru_Hel1Ac*, were identified in *S. frugiperda* together with a shorter core sequence sharing high identity with these three exemplars. Multiple sequence alignment showed that these three exemplar *Helitrons* have high sequence identity in shared sequences, but differ due to additional captured regions internal to the elements. The gene fragment trapped within *Helitrons* excluded the end bypass model. Alternatively, filler DNA model suggests that *Helitrons* acquire DNA from the host during the repair of double-strand breaks (DSBs) internal to the element, and predicts that short regions flanking the DSB in the acceptor transposon should be homologous to DNA sequences flanking the original host sequence captured by the transposon [6]. The identification of end junctions in the putative source loci suggested that *Hel1 Helitrons* acquire DNA from the host putatively by filler DNA insertion during the repair of DSBs. Notably, the average percentage divergence of these three exemplars were 0.852, 0.164 and 0.016, respectively, strongly supporting the occurrence of stepwise transposition and amplification putatively using the core sequence as the source element. However, while shorter core sequences were also identified in respective host genomes, exemplars of the *Pxut_Hel1A* and *Csup_Hel1* seems to capture host gene fragment during independent transposition events.

No less than 2836 horizontal transposon transfer (HTT) events have been recorded so far in multicellular eukaryotes [34], however, the mechanisms underlying HTT remain largely mysterious. The role of a host-parasite relationship has been proposed recently as a major mechanism of horizontal DNA transfer [21, 35, 36]. In this study, we provide evidence that *Prap_Hel1Aa* might derive from *Cves_Hel1Aa*. While *C. vestali* is larval parasitoid of the diamondback moth, *Plutella xylostella* (Lepidoptera: Plutellidae), we did not find any *Cves_Hel1*-like sequences in the genome database of *P. xylostella* (http://iae.fafu.edu.cn/DBM/), putatively due to the evolutionary dead-end of parasitized caterprillars. On the other hand, parasitoids are likely to oviposit within marginal (or even completely unsuitable) hosts in the laboratory or field, even if suitable hosts are present [37], and *C. vestalis* has been reared from several species belonging to different Lepidopteran families [38], thus we propose that *C. vestalis* might be a nonregular parasite of *P. rapae*, and this nonregular host-parasite interactions contribute to the HT of *Hel1* between these two species. The origin of *Cves_Hel1Aa* in *C. vestalis* seems to be a mystery. A number of core sequences were found in Lepidoptera genome including *S. frugiperda*, *P. xuthus* and *C. suppressalis*, thus as a vector for HT in Lepidoptera insects, *C. vestalis* is more likely to acquire and transfer *Cves_Hel1Aa* to *P. rape* from other Lepidoptera insects.

While our results indicate the role of nonregular host-parasite interactions in HT of *Prap_Hel1Aa* and *Cves_Hel1Aa*, the evidence of 2 additional cases of HTT (*Cvic_Hel1Ca* and *Bmor_Hel1Ca*, *Pgla_Hel1Ga* and *Hvit_Hel1Ga*) based on their patchy distribution and incongruence of *Hel1* and host phylogeny is somewhat intriguing due to the absence of host-parasite relationship among these species. It has been proposed that mechanisms of HT include insect-associated facultative symbionts [39–45]. In addition, the *Lep*1-like elements identified in the genome of *Nosema bombycis* suggested that the intracellular microsporidia parasite is also a potential vector for HT [21]. Recent studies have also suggested that both baculovirus and polydnaviruses might be important vectors of HTT [46–48]. While *Lep*1-like and *Hel-2 Helitrons* had been identified in *C. vestalis* and *Cotesia sesamiae* bracovirus and AcNPV, respectively [20, 21], we did not find *Hel1* in the genomes of bracovirus and NPV. However, the discovery of *Hel1*-like sequence in TSA database of *E. muscae* suggests that pathogen may also serve as a vector mediating HT of insect TEs. More widespread sequencing would be required to find exact vectors that would facilitate the HT of *Hel1 Helitrons* in these species.

## Conclusion

In the current report, we conducted a thorough search for a novel *Helitron* family by analyzing the sequenced genomes of 256 insects and 22 spiders. We modified the classical classification system for family and subfamily definition of *Helitrons*, and classified *Hel1* family into 9 subfamilies and 34 exemplars, among which three exemplars in *S. frugiperda* exhibited stepwise sequence acquisition, supporting the filler DNA model. We proposed that nonregular host-parasite interactions plays an important role in HT of *Helitrons*. Our data may have implications for understanding the evolution and HT mechanisms of *Helitrons*.

## Materials and methods

### Data resources

The publicly available 256 Insecta and 22 Arachnida WGS from National Center for Biotechnology Information (NCBI) (last accessed September 30, 2017) were used in this study. *P. rapae* and *P. xuthus* WGS were downloaded from NCBI. A list of the analyzed species and corresponding amount of sequence data is provided in Additional file 4: Data S3 online. As corresponding gene annotation files, we used the GFF files GCF_001856805.1 for *P. rapae* and GCF_000836235.1 for *P. xuthus*, respectively.

### Database searches and copy number estimation of *Helitrons*

Database searches were performed and comprise three steps. Firstly, the novel *Helitron* sequence located downstream of a *SINE* in *C. suppressalis* was used as a query in BLASTN searches against the NCBI *C. suppressalis* WGS database. Sequences of high homology as well as 200 bp upstream and downstream flanking regions were extracted and analyzed for hallmarks of *Helitrons* such as characteristic 5′-TC and 3′-CTRY nucleotide termini, and the consensus sequences of three *Helitron* exemplars of *Hel1* families in *C. suppressalis*, *Csup_Hel1Aa*, *Csup_Hel1Ab* and *Csup_Hel1Ea*, were determined. Secondly, a total of 255 insect WGS collections were searched using 161 bp common sequence of *Csup_Hel1Aa* and *Csup_Hel1Ab* (Additional file 1: Figure S18) as query to detect sequences with high identity with *Csup_Hel1Aa* and *Csup_Hel1Ab* in other insect species, and 9 subfamilies, *Hel1A*-*Hel1I* were identified. Finally, WGS collections of other invertebrates were searched using 161 bp common sequence of *Csup_Hel1Aa* and *Csup_Hel1Ab* as query to detect *Hel1*-like sequences in other species, and the second family *Hel2* was identified. In total, 2 families, 9 subfamilies, and 35 *Helitron* exemplars were identified, and consensus sequences for each *Helitron* exemplars were reconstructed based on a multiple alignment of at least 10 individual copies [36]. Specially, the copies of *Aros_Hel1Aa* and *Bmor_Hel1Aa* were less than 10, thus all copies were used for multiple alignment to determine consensus sequence. All consensus sequences are provided in Additional file 5: Data S4.

To estimate copy number and average percentage divergence of *Helitrons*, we used respective consensus sequences to search against related genomes where these *Helitron* elements were found using BLASTN. All contiguous fragments with at least 80% identity at the nucleotide level to the consensus over 100 bp were used to estimate copy number in all species [36, 49]. Given that 3′-ends deletion occurred in several copies of different subfamilies/exemplas in the same organism species, all those undistinguishable copies were counted as members of families. For example, two *Helitron* exemplars in *P. xuthus*, *Pxut_Hel1Aa* and *Pxut_Hel1Ab* shared high identity of 128 bp sequence at 5′-ends, thus all copies aligned only with part or full of this 128 bp region in the consensus sequence were estimate as members of family (Additional file 1: Figure S19). Furthermore, all fragments sharing at least 80% identity over at least 80% of the length of the consensus sequence were aligned and used for average percentage divergence calculation with Kimura-2 parameter model [50] in all species except *A. rosae, Blattella germanica, Locusta migratoria, P. tepidariorum, Pseudomyrmex gracilis, T. cristinae* and *Phortica variegata*, in which a high level of fragmentation was observed in multiple *Helitron* copies.

### Reconstruction of potential autonomous *Helitron*

The reconstruction of autonomous *Helitron* comprise three steps. Firstly, large DNA fragments ranging from 1000 bp to 10 kb that shared similar terminal sequences to the above families were retrieved from WGS databases, and their potential transposase were predicted using getorf in EMBOSS-6.3.1 package [51]. Secondly, these candidates with degenerated remnants of *Helitron* coding sequences were used as queries in BLAST searches against both WGS databases, TSA and non-redundant protein databases. Finally, the query sequence and hit sequences were aligned to reconstruct the uninterrupted coding sequences with complete *Rep/helicase* gene ORF of *Helitron* by removing frameshifts and insertions.

### Nomenclature

To distinguish *Helitron* elements from 29 species, we assume a set of concept names that consist of short Latin of single species, the type of TEs, the family, subfamily and exemplars of *Helitron*, just like *Csup_Hel1Aa*. Given that the 3′-end sequences of *Helitrons* were more variable than the 5′-end sequences [12], and the 5′-end sequence was strictly necessary for *Helitron* transposition [9], we modified Yang and Bennetzen′s method to reclassify *Helitron* TEs [25]. Generally, the sequences with the most similar 5′-ends (30 bp with at least 80% identity) were classified as members of the same family and sequences with the most similar 3′-ends (30 bp with at least 80% identity) were classified as members of the same subfamily. Due to the internal sequence divergence of copies in the same *Helitron* subfamily, the unique internal sequences with more than 80% identity were classified as members of exemplars.

### Gene association and genomic show cases

The site of the *Helitron* integration relative to annotated genes was analyzed with a custom Perl script [29]. All copies of *Prap_Hel1Aa*, *Pxut_Hel1Aa* and *Pxut_Hel1Ab*, were determined for their positions in the genome through BLAST analysis with respective genome database and the GFF annotation files. The *Helitrons* in coding and untranslated gene regions as well as the distances of intergenic copies to the closest neighboring gene were determined and the numbers were counted [29]. All the genic and genomic loci harboring *Helitrons* were refined and visualized with the respective annotations using Perl script. All the figures used CorelDRAW to beatify the fine tune.

### Sequence analysis and phylogeny

RNAstructure (http://rna.urmc.rochester.edu/RNAstructureWeb) was used to predict and analyze DNA secondary structure [52]. Multiple alignment of *Helitrons* were created by MUSCLE [53], and subsequently visualized with GENEDOC (www.psc.edu/biomed/genedoc) and TeXshade [54].

The phylogeny of *Helitron* elements was built using MrBayes 3.2 [55] after removing ambiguously aligned regions using BMGE [56] (Additional file 5: Data S5). Nucleotide substitution models were chosen using the AIC criterion in Modeltest [57] (HKY + G). The robustness of the nodes was evaluated for all phylogenies by performing a bootstrap analysis involving 1000 pseudo replicates of the original matrix [36].

Specifically, in the evolutionary analysis of subfamilies from *P. xuthus and C. suppressalis,* we conducted local BLAST analysis and got a CSV file based on location information to obtain all sequences that are larger than 80% coverage of and 80% identity to the consensus sequences. Finally, we extracted these sequences from each genome using TBtools [58]. A Neighbor-Joining (NJ) phylogenetic tree of these sequences in *C. suppressalis* and *P. xuthus* were constructed using MEGA 7.0.

### Detection of insertion polymorphism of *Prap_Hel1Aa*

In *P. rapae*, using one pair of primers flanking the insertion site (Forward primer: 5′-ACGAGAGATGGCTACAACAG-3′; Reverse primer: 5′- AACACACCCACACCCTAAAC -3′), the insertion polymorphism of one short copy of *Prap_Hel1Aa* (Prap0202, LWME01000202.1: 138955–138,682) was assessed by performing a PCR survey. The PCR products were cloned into the pMD18-T vector (TaKaRa, Dalian, China) and sequenced.

## Additional files

Additional file 1: **Figure S1.** Insertion of a novel *Helitron* element into downstream region of a *SINE* element in *C. suppressalis* genome. The *SINE* and *Helitron* sequences are highlighted in blue and pink, respectively. The nucleotides highlighted in purple are the target site duplication (TSD) of *SINE*. **Figure S2.** Characteristic of *Csup_Hel1A*. (a), Multiple alignment of 30-bp end sequences as well as the flanking host nucleotides at the 5′- and 3′-end from *Csup_Hel1A* elements. The alignment was graphically edited using TeXshade package. (b), Predicted secondary structure of 30-bp region at the 3′-end of *Csup_Hel1A* consensus sequence. **Figure S3.** The distribution of *Csup_Hel1A*-like Helitrons in insect and spider genomes. Taxa showing *Hel1A*-like *Helitrons* are colored taxonomically, with Lepidopteran insects in red, Diptera insects in purple, Hymenoptera wasps in yellow, Araneae species in green. **Figure S4.** Multiple alignment of 126 bp region at the 5′-end consensus sequence of *Hel1* Helitrons. **Figure S5.** Multiple alignment (a) and genetic distance analysis (b) of 30-bp region at the 5′-end of consensus sequences of *Ptep_Hel2Ca* and *Csup_Hel1Ab*. **Figure S6.** Structural analysis of degenerated remnants potential autonomous *Helitrons* found in *N. clavipes*, *P. tepidariorus, P. machaon, C. vestalis, H. vitripennis, A. rosae* and *T. cristinae*. **Figure S7.** Multiple alignment (a) and genetic distance analysis (b) of Rep/helicase protein sequences of reconstructed potential autonomous *Helitrons*. **Figure S8.** The typical integration pattern of *Hel1A* within genomes of *P. xuthus* and *P. rapae*. (a), A copy of *Pxut_Hel1Aa* inserted into the coding sequence (CDS) of a gene. (b), A copy of *Pxut_Hel1Ab* inserted into intron. (c), Several copies of *Prap_Hel1Aa* inserted into introns and exons of the same gene. **Figure S9**. Identification of source loci and end junctions of insertions in *Sfru_Hel1A*. The end junction sequences are shaded. **Figure S10.** Multiple alignment of *Pxut_Hel1Ab* consensus sequence and core sequence in *P. xuthus* (a), *Csup_Hel1Aa* and *Csup_Hel1Ab* consensus sequences in *C. suppressalis* as well as core sequence in genome of *P. xuthus* (BBJE) and *S. frugiperda* (FJUZ) (b) and *Csup_Hel1Ea* consensus sequence and four short core sequences in *C. suppressalis* (c). **Figure S11.** Paralogous or orthologous empty sites of *Prap_Hel1Aa* in *P. rapae*, *Cves_Hel1Aa* in *C. vestalis, Csup_Hel1Aa* in *C. suppressalis*, and *Mcin_Hel1Ca* in *Melitaea cinxia*. The 4-letter project ID of WGS accession number and corresponding species are listed as following: LWME for *P. rapae*, JZSA for *C. vestalis*, ANCD for *C. suppressalis*, APLT for *Melitaea cinxia,* JXPT for *Bactrocera oleae* and AZMT for *Microplitis demolitor.* **Figure S12.** Multiple alignment of orthologous gene of calreticulin from *P. rapae* (EU826537.1) and *C. vestalis* (KX384605.1) (a), heat shock protein 70 from *P. rapae* (KJ573767.1) and *C. vestalis* (JX088378.1) (b), opsin from *P. rapae* (AB177984.1) and *C. vestalis* (KY368220.1) (c), consensus sequences of *Prap_Hel1Aa* and *Cves_Hel1Aa* as well as a short copy of *Prap_Hel1Aa* (Prap0202, LWME01000202.1: 138955–138,682) (d) and Prap0202 and eight individual sequences of *Cves_Hel1Aa* including Cves_Hel1Aa.1(JZSA01006637.1: 22335–22,612), Cves_Hel1Aa.2 (JZSA01007293.1: 718–441), Cves_Hel1Aa.3 (JZSA01002845.1: 21212–21,486), Cves_Hel1Aa.4 (JZSA01005118.1: 18777–18,500), Cves_Hel1Aa.5 (JZSA01002791.1: 17198–17,475), Cves_Hel1Aa.6 (JZSA01001525.1: 718–441), Cves_Hel1Aa.7 (JZSA01000595.1: 12281–12,558) and Cves_Hel1Aa.8 (JZSA01006408.1: 3165–2888) (e). **Figure S13.** Phylogenetic analysis of all copies of *Hel1* Helitrons in *C. vestalis*, *P. rapae* and *C. suppressalis*. The phylogenetic tree was constructed by the neighbor-joining method using MEGA 7.0 software. **Figure S14.** Detection of orthologous empty site of a short copy of *Prap_Hel1Aa* (Prap0202, LWME01000202.1: 138955–138,682) in *P. rapae* larvae collected from Yangzhou, China. **Figure S15.** Multiple alignment of sequences completely same as the consensus sequence of *Prap_Hel1Aa* in *P. rapae.* **Figure S16.** Multiple alignment of *Emus_Hel1Ca* and the consensus sequences of *Bmor_Hel1Ca*, *Cvic_Hel1Ca* (a) as well as *Emus_Hel1Ca* and the individual sequences of *Bmor_Hel1Ca* (Bmor_Hel1Ca.1, AADK01000158.1: 43487–43,268) and *Cvic_Hel1Ca* (Cvic_Hel1Ca.1, JXOT01107662.1: 1253–1516; Cvic_Hel1Ca.2, JXOT01181287.1: 382–645) (b). **Figure S17.** Multiple alignment (a) and genetic distance ananlysis (b) of *Pgla_Hel1Ga* and *Hvit_Hel1Ga*. **Figure S18.** Multiple alignment of *Csup_Hel1Aa* and *Csup*_*Hel1Ab*. **Figure S19.** Multiple alignment of *Pxut_Hel1Aa* and *Pxut_Hel1Ab* from *P. xuthus*. (PDF 3085 kb)

Additional file 2: **Table S1.** List of copies of the *Helitron* based on searches of WGS database in *C. suppressalis.* **Table S2.** Estimates of evolutionary divergence between *Hel1* transposon of 28 speices . The number of base differences per site from between sequences are shown. The analysis involved 34 nucleotide sequences. Codon positions included were 1st + 2nd + 3rd + Noncoding. All ambiguous positions were removed for each sequence pair. There were a total of 131 positions in the final dataset. Evolutionary analyses were conducted in MEGA7.0. (XLSX 66 kb)

Additional file 3: **Data S1.** The *Hel1* elements with degenerated remnants of *Helitron* coding sequences identified in insect and spider genome databases. **Data S2.** The reconstructed potential autonomous *Hel1 Helitrons*. (PDF 67 kb)

Additional file 4: **Data S3.** The list of the analyzed species and corresponding amount of sequence data. (XLSX 2613 kb)

Additional file 5: **Data S4.** The consensus sequences of 35 *Helitron* exemplars. **Data S5.** The sequences used for evolutionary analysis. (PDF 101 kb)
