# Supplementary material for: Characterization of a novel Helitron family in insect genomes: insights into classification, evolution and horizontal transfer
Source: Mob DNA. 2019 May 31;10:25. doi: 10.1186/s13100-019-0165-4 (PMC6544945; doi:10.1186/s13100-019-0165-4)
Supplement: Supplementary file 1 — Figure S1. Insertion of a novel Helitron element into downstream region of a SINE element in C. suppressalis genome. The SINE and Helitron sequences are highlighted in blue and pink, respectively. The nucleotides highlighted in purple are the target site duplication (TSD) of SINE. Figure S2. Characteristic of Csup_Hel1A. (a), Multiple alignment of 30-bp end sequences as well as the flanking host nucleotides at the 5′- and 3′-end from Csup_Hel1A elements. The alignment was graphically edited using TeXshade package. (b), Predicted secondary structure of 30-bp region at the 3′-end of Csup_Hel1A consensus sequence. Figure S3. The distribution of Csup_Hel1A-like Helitrons in insect and spider genomes. Taxa showing Hel1A-like Helitrons are colored taxonomically, with Lepidopteran insects in red, Diptera insects in purple, Hymenoptera wasps in yellow, Araneae species in green. Figure S4. Multiple alignment of 126 bp region at the 5′-end consensus sequence of Hel1 Helitrons. Figure S5. Multiple alignment (a) and genetic distance analysis (b) of 30-bp region at the 5′-end of consensus sequences of Ptep_Hel2Ca and Csup_Hel1Ab. Figure S6. Structural analysis of degenerated remnants potential autonomous Helitrons found in N. clavipes, P. tepidariorus, P. machaon, C. vestalis, H. vitripennis, A. rosae and T. cristinae. Figure S7. Multiple alignment (a) and genetic distance analysis (b) of Rep/helicase protein sequences of reconstructed potential autonomous Helitrons. Figure S8. The typical integration pattern of Hel1A within genomes of P. xuthus and P. rapae. (a), A copy of Pxut_Hel1Aa inserted into the coding sequence (CDS) of a gene. (b), A copy of Pxut_Hel1Ab inserted into intron. (c), Several copies of Prap_Hel1Aa inserted into introns and exons of the same gene. Figure S9. Identification of source loci and end junctions of insertions in Sfru_Hel1A. The end junction sequences are shaded. Figure S10. Multiple alignment of Pxut_Hel1Ab consensus sequence and core sequence in P. [file 13100_2019_165_MOESM1_ESM.pdf]

>*Chilo suppressalis* strain Taihu contig113693 (ANCD01113693.1: 1081-2280)
ctttggtacatGAGGAGCTCGATGGCGCAAGTGGTTAGCGCGTTCGGCTGCGATCGTTGAAGT
TAATCAACTTTCGCAGTGGTCGGCTATTGGATGGGTGACCAAAAACTACTATCTCGAGC
TCCTCCGTGCTCCGGAAGGCACGTTAAGCCGTTGTTCCCGGTCTGCATCTGCAGTCGTT
AGCACCCACCTATCCGCACTGGGCCTGCAGGGTGGGTCACGGCCCATTCTCCCTATTCC
ATCCATAGGGAAGGCCTGTGCTCCAGCAGTGGGGACATTAATAGGATGATGATGacataatg
gcactaaaataatcgttctaaatattaattagttgaatcataacgaattatcgagctgcggtatgatagacgctagacgatactttgactggtgatttt
tggcacgcataaaagagcttgtgggaaacgttaaaattcgtatgtgcagctaaaggatactaattcaaatttcaaacgctaatttaagcgctgtta
agtaccaaagcttcagaagcattactgaaagagactatcactggccacgttctgtgttcgttcgccaccgtctagcccacaccctcctgcaccg
gaaactccgtgtgtgtaacatggacactattcacaaccctcttctacgtgataagataacacacagcacataagaagactttttgtctgagttatg
ccttatgcatccttcaaacaccggaggagcgagaagatatgaggcgctcctcactgcttatttcagcaagccctagggctgtctgaaatgaaca
gggccactgaaaaccagacaatactcctttgctaggatcgcttccttcttgtttttaatgaatcttttccacaactcgacaatttgttgatgcctgtatt
gcaagctattttagcccgaatttttgttttctactacaagaagttctgaacctatccatatatacctaaTCTTTATATATATAATTCTT
CTGTGAGTGTGTATGTCACTGAACTTCTCTTAAACGACTGGACCGATTTTGATGAAATT
TTTTGTGTGTGTTCAAGGGGATCTGAGAATGGTTTAGATTCACAATTTTGTCCGCTGGA
CAATGTTTTTTTAATTAATTTTTAATTTATTAGTAGTTGTTGATTTTGGAATGTTTTACATT
GGATCCGACAGACGGCGCTACCATCGCAGTGTCAAATTTTCAAATTAAAGACGTGTAG
ACAGGACAACGTCTGTCGGGTCCGCTAGtttatatacgtaagaatagagttat

Figure S1

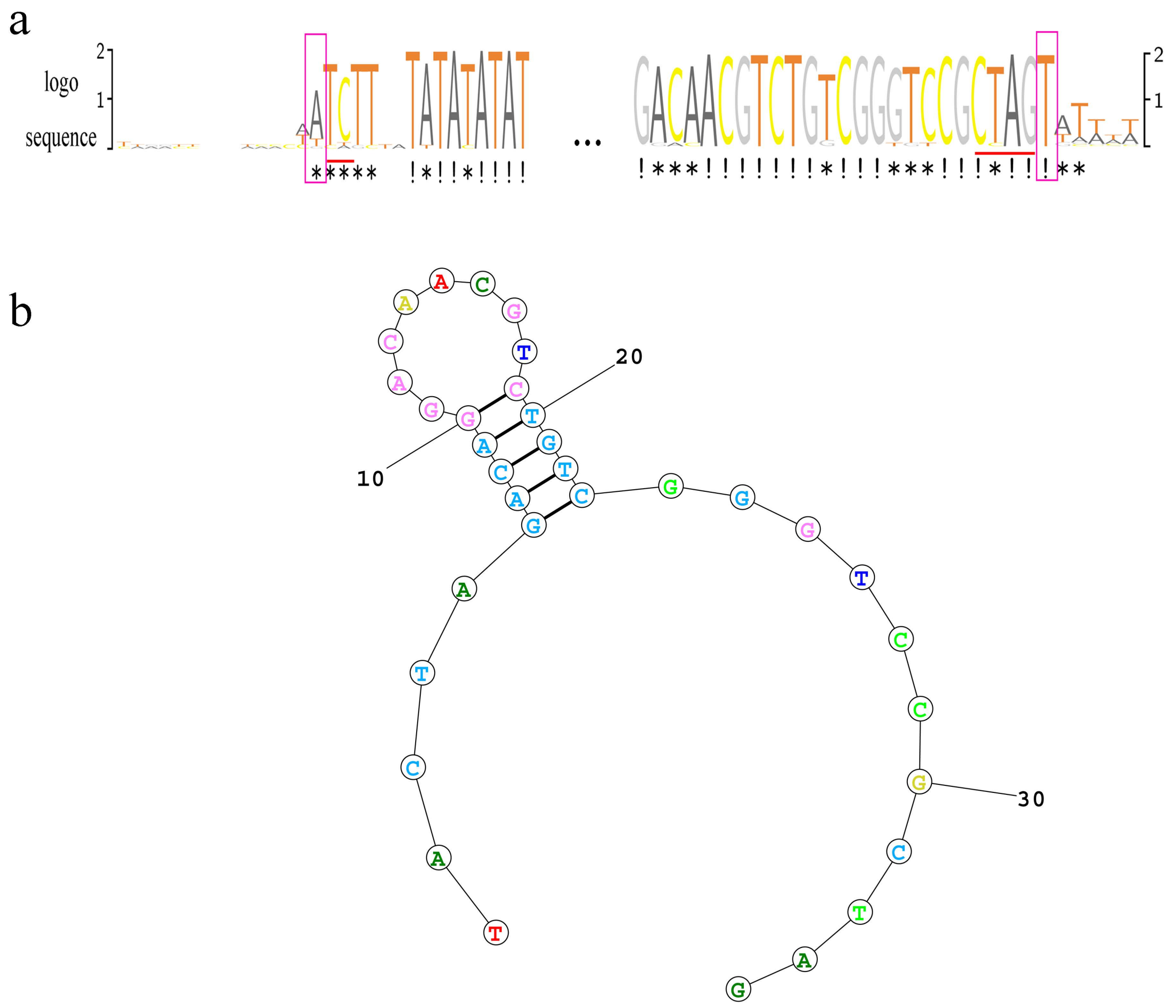


Figure S2

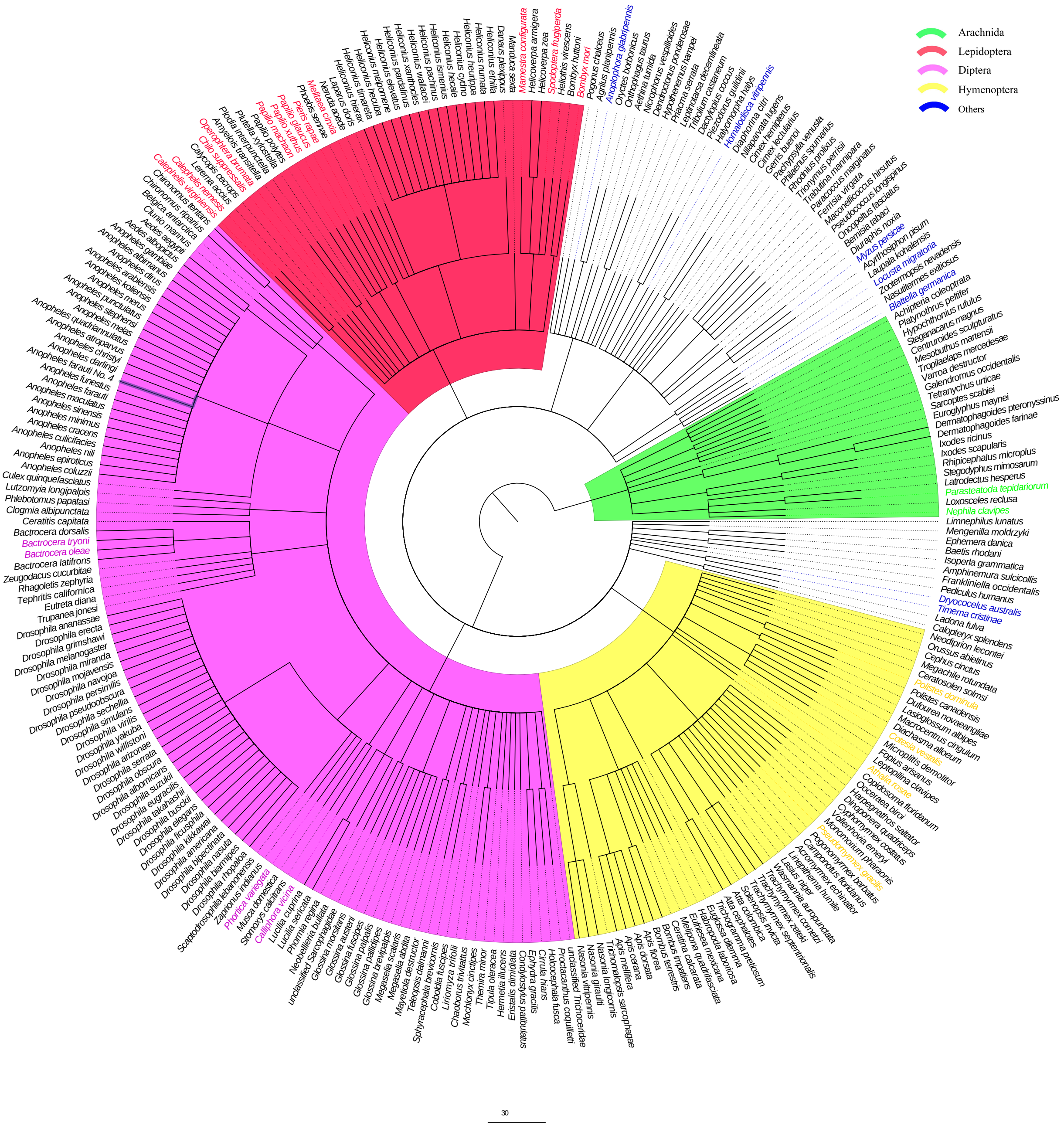


Figure S3

```
                       *        20         *        40         *        60
Agla_Hel1Aa : TCTTT--ATATATAT--AATTCTTCTGT-CGTGTGTATGTCACTGAACTCCTCCTAAACGGCTGG :  60
Aros_Hel1Aa : TCTTA--ATATATAT--ATTTCTTGTGTGCGTGTGTATGTGACTGAACTCCTCCTAAACGACTGG :  61
Bole_Hel1Aa : TCTTTATAAATATAT--ATTTCTTGTGTGCGTGTGTATGTCACTGAACTCCTCCTAGACGGCTGG :  63
Btry_Hel1Ba : TCTTT--ATATATAT--AAATCTTCTGACCGTGTGTTTGTAATTGAACTGCTCCTAAACGGCTGG :  61
Bger_Hel1Aa : TCTTA--ATATATAT--ATTTCTTGTGTGCGTGTGTATGTGACTGAACTCCTCCTAAACGACTGG :  61
Bmor_Hel1Ca : TCTTTATAT-TATAT--ATTTCTTCTGTGCGTGTGTTTGTCACTGAACTCCTCCTAAACGGCTGG :  62
Bmor_Hel1Aa : TCTT---ATTTATATATAATTCTTCTGTCCGTGTGTATGTTAGTGAGCACCTCCTAAACGGCTAG :  62
Cnem_Hel1Ea : TCTTT--ATATATAT--AATTCTTCTGTGAGTGTGTATGTCACTGAACTCCTTCTAGACGACTGG :  61
Cvir_Hel1Ea : TCTTT--ATATATAT--AATTCTTGTGTTCGTGTGTTTGTCACTGAACTCCTTCTAGACGACTGG :  61
Cvic_Hel1Ca : TCTTT--ATATATAT--AATTCTTCTGTACGTGTGTTAGTAACTGAACTCCTCCTTAACGGCTGG :  61
Csup_Hel1Aa : TCTTT--ATATATAT--AATTCTTCTGTGAGTGTGTATGTCACTGAACTTCTCTTAAACGACTGG :  61
Csup_Hel1Ab : TCTTT--ATATATAT--AATTCTTCTGTGAGTGTGTATGTCACTGAACTTCTCTTAAACGACTGG :  61
Csup_Hel1Ea : TCTTT--ATATATAT--AATTCTTCTGTGAGTGTGTATGTCACTGAACTTCTCTTAAACGACTGG :  61
Cves_Hel1Aa : TCTT---ATATATAT--ATTTCTTGTGTG-GTGTGTATGTGACTGAACTCCTCCTAAACGACTGG :  59
Daus_Hel1Aa : TCTTTATATATATAT--AATTCTTCTGTGCGTGTGTATGTCACTGAACTCCTCCTAAACGGCTGG :  63
Hvit_Hel1Ga : TCTTT--ATATATAT--ATTTCTTGTGTTCGTGTGTATGTGACTGAACTCCTCCTAAACGACTGG :  61
Lmig_Hel1Aa : TCTTA--ATATATAT--ATTTCTTCTGTCCGTGTGTTTGTCACTAAACTCCCCCTAAACGGCTGG :  61
Mcon_Hel1Aa : TCTTAATATATATATA-TTTTCTTGTGTACGTGTGTATGTCACTGAACTCCTCCTAAACGGCTGG :  64
Mcin_Hel1Ca : TCTTTATATATATAT--ATTTCTTGTGTGCGTGTGTATGTCACTGAACTCCTCCTAGACGGCTGG :  63
Mper_Hel1Fa : TCTTT--ATATATAT--AATTCTACTGTACGTGTGTATGTCACTGAACTCCTCCTAAACGGCTGG :  61
Ncla_Hel1Aa : TCTTT--ATATATGT--AATTCTTCTGTGCGTGTGTATGTAACTGAACTCCTCCTAAACGGCTGG :  61
Obru_Hel1Ia : TCTTA--ATATATAT--AATTCTTCTGTACGTGTGT-TGTCACTGAACTCCTCCTAAACGGCTGG :  60
Pgla_Hel1Ga : TCTTT--ATATATAT--ATTTCTTGTGTGCGTGTGTATGTCACTGAACTCCTCCTAGACGGCTGG :  61
Pmac_Hel1Ha : TCTTA--ATATATAT--ATTTCTTGTGTGCGTGTGTATGTGACTGAACTCCTCCTAAACGACTGG :  61
Pxut_Hel1Aa : TCTTT--ATATATAT--AATTCTTCTGTGAGTGTGTATGTCACTGAACTTCTCTCAAACGACTGG :  61
Pxut_Hel1Ab : TCTTT--ATATATAT--AATTCTTCTGTGAGTGTGTATGTCACTGAACTTCTCTCAAACGACTGG :  61
Pvar_Hel1Da : TCTTA--ATATATAT--ATTTCTTCTGTGCGTGTGTTTGTCACTGAACTCCTCCTAAACGGCTGG :  61
Prap_Hel1Aa : TCTTA--ATATATAT--ATTTCTTGTGTGCGTGTGTATGTGACTGAACTCCTCCTAAACGACTGG :  61
Pdom_Hel1Aa : TCTTTATATATATATATAATTCTTCTGTGCGTGTGTATGTCACTGAACTCCTCCTAAACGGCTGG :  65
Pgra_Hel1Aa : TCTTA--ATATATAT--ATTTCTTGTGTGCGTGTGTATGTCACTGAACTCCTCCTAAACGACTGG :  61
Sfru_Hel1Aa : TCTTT--ATATATATA-AATTCTTCTGTAAGTGTGTATGTCACTGAACTTCTCTTAAACGACTGG :  62
Sfru_Hel1Ab : TCTTT--ATATATAT--AATTCTTCTGTACGTGTGTATGTCACTGAACTTCTCTTAAACGAGTGG :  61
Sfru_Hel1Ac : TCTTTATATATATA---AATTCTTCTGTAAGTGTGTATGTCACTGAACTTCTCTTAAACGACTGG :  62
Tcri_Hel1Aa : TCTTA--ATATATAT--AATTCTTCTGTACGTGTGTATGTCACTGAACTCCTCCTAAACGGCTGG :  61
core sequence TCTT   AtaTATat  a tTCTt TGt  GTGTGTatGT AcTgAaCt Ctc taaACG cTgG

                       *        80         *       100         *       120    *
Agla_Hel1Aa : ACCGATTTTGATGAAATTTTTTGTGTGTGTTCAAGTGGATTCGAGAATGGTTTAGATTCACAATT : 125
Aros_Hel1Aa : ACCAATTTTGATGAAATTTTTTGTGTGTGTTCGTGGAGATTCGAGAATGGTTTAGATTTACAGTT : 126
Bole_Hel1Aa : ACCGATTTTGATGAAATTTTTTGTGTGAGTTCAAGGGGATTCGAGGATGGTTTAGATTCACAATT : 128
Btry_Hel1Ba : ACCGATTTTGATGAAATTTTTTGTGTGTGTTTAAGGAGATTCGAGGATGGTTTAGATTCACAATT : 126
Bger_Hel1Aa : ACCGATTTTGATGAAATTTTTTGTGTGTGTTCGTGGAGATTCGAGAATGGTTTAGATTTACAGTT : 126
Bmor_Hel1Ca : ACCGAT-TTAATGA--TTTTTTGTATCTTT----GGTGGCCCCTGGATGGTTTAGATTCACAAAT : 120
Bmor_Hel1Aa : ACTGATTTTGATGAAATTTTGTGTGTGTATTCCAACGGGTTCGAGAATGGTTTAGATTCACAATT : 127
Cnem_Hel1Ea : ACCGATGTCGATGAAACTTTTTGTATGTGTTCTAGGGAATTCGAGAATGGTTTAGATTCACAATT : 126
Cvir_Hel1Ea : ACCGATGTTGATGAAACTTTTTGTGTGTGTTCAAGGGAATTCGAGAATGGTTTAGATTCACAATT : 126
Cvic_Hel1Ca : GCCGATTTCGATGAAATTTGTTGTGTGTGTTTGAGTGGGTCCCTGGATGGTTTAGATTCACAATT : 126
Csup_Hel1Aa : ACCGATTTTGATGAAATTTTTTGTGTGTGTTCAAGGGGATCTGAGAATGGTTTAGATTCACAA-- : 124
Csup_Hel1Ab : ACCGATTTTGATGAAATTTTTTGTGTGTGTTCAAGGGGATCTGAGAATGGTTTAGATTCACAATT : 126
Csup_Hel1Ea : ACCGATTTTGATGAAATTTTTTGTGTGTGTTCAAGGGGATCTGAGAATGGTTTAGATTCACAATT : 126
Cves_Hel1Aa : ACCGATTTTGATGAAATTTTTTGTGTGTGTTCAAGGGGATCTGGGAATGGTTTAGATTCACAATT : 124
Daus_Hel1Aa : ACCGATTTTGATGAAATTTTTTGTGTGTGTTCAAGTGGATTCGAGAATGGTTTAGATTCACAATT : 128
Hvit_Hel1Ga : ACCGATTTTGATGAAATTTTGTGTGTGTTTTCAATGGAATTCGAGAATGGTTTAGATTCACAATT : 126
Lmig_Hel1Aa : AC-GATTTTCATGAAATTTTTTGTGTGTGTTTAAGGGGATTCGAGAATGGTTTAGATTCACAATT : 125
Mcon_Hel1Aa : ACCGATGTTGATGAAATTTTTTGTGTGTGTTCAAGGGGATTCGAGAATGGTTTAGATTCACAATT : 129
Mcin_Hel1Ca : ACGGATTTTGATGAAATTTTTTGTGTGAGTTCAAGGGGATTCGAGGATGGTTTAGATTCACAATT : 128
Mper_Hel1Fa : ACCGATTTGAATGAATTTTTTTGTATGCGTTTGGGTGACGCCCTGGATGGTTTAGATTCACAATT : 126
Ncla_Hel1Aa : ACCGATTTTGATGAAAGTTTTTGTCTG--TTCCAGTGGACTCGAGAATGGTTTAGATTTATAATT : 124
Obru_Hel1Ia : ACCGATTTTGATGTTTTTTTTTGTGTGT-TTTGAGTGGGTCCCTGGATGGTTTAGATTCACAACT : 124
Pgla_Hel1Ga : ACCGATTTTGATGAAATTTTTTGTGTGAGTTCAAGGGGATTCGAGGATGGTTTAGATTCACAAT- : 125
Pmac_Hel1Ha : ACCAATTTTGATGAAATTTTTTGTGTGTGTTCGTGGAGATTCGAGAATGGTTTAGATTTACATTT : 126
Pxut_Hel1Aa : ACCGATTTTGATGAAATTTTTTGTGTGTGTTCAAGGGGATCTGGGAATGGTTTAGATTCACAATT : 126
Pxut_Hel1Ab : ACCGATTTTGATGAAATTTTTTGTGTGTGTTCAAGGGGATCTGGGAATGGTTTAGATTCACAATT : 126
Pvar_Hel1Da : ACCGATTTTGATGAAATTTTTTGTGTATGTTCAAGGGGATTCGAGAATGGTTTAGATTCACAATT : 126
Prap_Hel1Aa : ACCGATTTAGACGAAATTTTTTGTGTGTGTTCAAGGGGATCTGGGAATGGTTTAGATTCACAATT : 126
Pdom_Hel1Aa : ACCGATTTTAATGAAATTTTTTGTGTGTCTTCAGGTGGATTCGATAATGGTTTAGCTTTACAATT : 130
Pgra_Hel1Aa : ACCGATTTTGATGAAATTTTTTGTGTGTGTTCAAGGGGATTTGAGAATGGTTTAGATTCACAATT : 126
Sfru_Hel1Aa : ACCGATTTTGATG-AATTTTTTGTGTGTGTTCAAGGGGATCTGAGAATGGTTTAGATTCACAATT : 126
Sfru_Hel1Ab : ACCGATTTTGATGAACTTTTTTGTGTGTGTTCAAGGGGATCTGAGAATGGTTTAGATTCACAATT : 126
Sfru_Hel1Ac : ACCGATTTTGATGA-ATTTTTTGTGTGTGTTCAAGGGGATCTGAGAATGGTTTAGATTCACAATT : 126
Tcri_Hel1Aa : ACCGATTTTGATGAAATTTTTTGTGTGTGTTTGAGTGGGTCCATGGATGGTTTTGATTCACAATT : 126
core sequence aCcgATtTtgAtGaaatTTttTGTgTgtgTtc ag ggat  g g ATGGTTTaGaTTcAcAatt
```

Figure S4

**a**

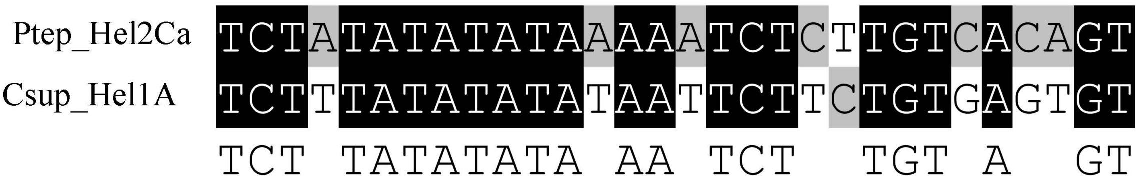


**b**

| | Ptep_Hel2Ca | Csup_Hel1A |
|---|---|---|
| Ptep_Hel2Ca | | |
| Csup_Hel1A | 0.266667 | |

Figure S5

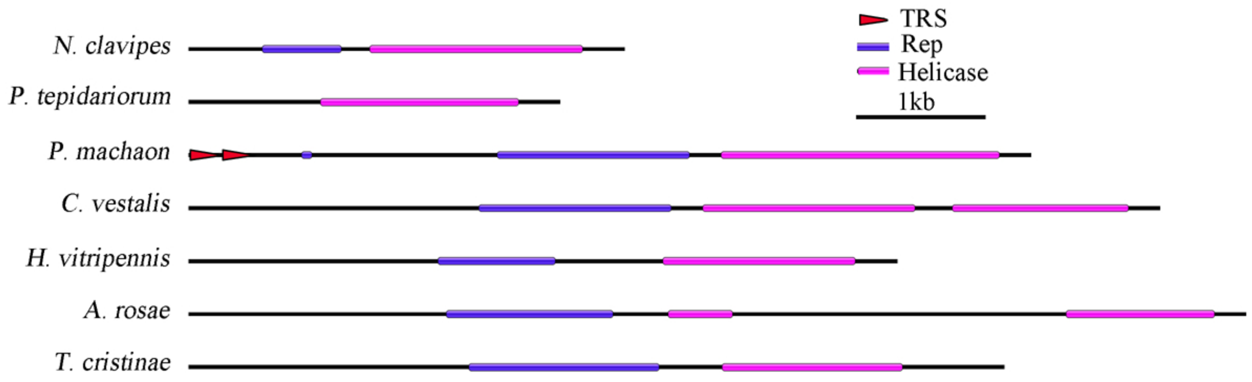

TRS
Rep
Helicase
1kb
N. clavipes
P. tepidariorum
P. machaon
C. vestalis
H. vitripennis
A. rosae
T. cristinae


Figure S6

**a**

```
                           *        20         *        40         *        60         *        80         *       100
C._vestalis     : MPRLRGRARNIGRRTQHAQLVHDHLLNRTVEEHSMDNVNLRDRAACTRANENLEQRAQLLRANTLRQREARQRATNAHRERNQQRVQDNRALARALLNRL :  100
P._machaon      : MPRLRGRARNIGRRTQHAQLVHDHRLNRTVEEHSMDNVNLRDRAACTRANENLEQRAQRLRANTLRQREARQRATNAHRERNQQRVQDNRALARASLNRL :  100
A._rosae        : MPRLRGRARNIGRRTQHAQLVHDHRLNRTVEEHSMDNVNLRDRAACTRANENLEQRAQRLRANTLRQREARQRATNADRERNQQRVQDNQALARASLNRR :  100
P._tepidariorum : MSRLRGRARNIGRRTRNAQLVHDRRLNRTVEEHLTDNVNSRNQVASSRENENLEQRNQRLRANALRQRETRQRATNENRERNQRRMQDNRALIRASLNRL :  100

                           *       120         *       140         *       160         *       180         *       200
C._vestalis     : AFEYDPGIDYSSHAFITIGSMDKECQHCHAFKYKGESAGLCCASRKISLSPLKPPPEPLKTLLAGITSQSKLFLRKIRKLNSCFQMTSFAATKIIHNGDG :  200
P._machaon      : AFEYDPEIDYSSHALITIGSMDKECQHCHAFKYKGESAGLCCASGKISLPPLKPPPEPLKTLLAGITSQSKLFLRKIRKLNSCFQMTSFAATKIIHNEDG :  200
A._rosae        : ACEYDPEIDYSSHALITIGSMDKECQHCHAYKYKGESAGLCCASGKISLPPLKPPPEPLKTLLAGITSQSKLFLRKIRKLNSCFQMLSSDTTKIIHNEDG :  200
P._tepidariorum : MFEYDPEIDYSSHALILIGSMDNECQYCHALKYKGESAGLCCASGKISLPPLNPSSEPFKTLLAGTASQSKLFLRKIRKSNSCFQMTSFGATKIVHNEGG :  200

                           *       220         *       240         *       260         *       280         *       300
C._vestalis     : RNFESTFKIQGQVYHQIGSLLPMPDADPKFLQIYFMGNEEQQSHTRCVYNHIEQMEEREIVDILERFLQNHNQLVQLFKTLSNRLQNDNYVIVIKADKVP :  300
P._machaon      : RNFESTFKIQGQVYHQIGSLLPMPDADPKFLQIYFMGNEEQQSHTRCVYNHIEQMEEREIVDILERFLQNHNQLVQLFKTLSNRLQNDNYVIVIKADKVP :  300
A._rosae        : RNFESTFKIQGQVYHQIGSLLPMPDADPKFLQIYFMGNEEQQSHKRCVYNHIEQMEEREIVDILKRFLKNHNQLVQLFKTLSNRLQNDNYVIVIKADKVP :  300
P._tepidariorum : RNFESTFKNQGQVYHQIGSLLPMPNVDPKFLQIYFMGDKEQQTHTRCIYNHIEQMEEREIVDILETFLQNHNQLIRLFKTLSNRLQNDNYAIVIKADKVS :  300

                           *       320         *       340         *       360         *       380         *       400
C._vestalis     : YGEHAGTYNVPTINEVAVVMAGDPCERRDIRIQRRDNTMQIIQDNHRSYDALQYPLIFWEAEDGYHLNIKQRNPTTGEELTKKVSAMNFYAYRLMIRANE :  400
P._machaon      : YGEHAGTYNVPTINEVAVVMAGDPCERRDIRIQRRDNTMQIIQDNHRSYDALQYPLIFWEGEDGYHLNIKQRNPTTGEELTKKVSAMNFYAYRLMIRANE :  400
A._rosae        : YGEHAGTYNVPTINEVAVVMAGDPCERRDIRIQRRDNTMQIIQDNHRSYDALQYPLIFWEGEDGYHLNIKQRNPTTGEELTKKVSAMNFYAYRLMIRANE :  400
P._tepidariorum : --SNAGTYNVPTVNEVAVVMAGDPCERRDIRIQRRDNTIQIIQDNHCSYDALQYPLIFWEGEDGYHLNIKQRNPTTGEELIKKVSAMNFYAYRLMIRENE :  398

                           *       420         *       440         *       460         *       480         *       500
C._vestalis     : DNNILRCRQLFHQYIVDMYVKIESERLRYIKFNQAKLRAEEYIHLRDAVIGNVDATNVINNIGTTYILPSSYIGSPRHMQEYIQDAMTYVRAYGRPDLFI :  500
P._machaon      : DNNILRCRQLFHQYIVDMYVKIESERLRYIKFNQAKLRAEEYIHLRDAVIGNVDATNVINNIGTTYILPSSYIGSPRHMQEYIQDAMTYVRAYGRPDLFI :  500
A._rosae        : DNNILRCRQLFHQYIVDMYVKIESERLRYIKFNQAKLRAEEYIHLRDAVIGNVDATNVINNIGTTYILPSSYIGSPRHMQEYIQDAMTYVRAYGRPDLFI :  500
P._tepidariorum : DNNILRSRQLFHQFIADMYVKIESERLRYINFNQAKLRSEEYIHLRDAIIGNVDATNDINNIGTAYILPSSYIGSPRHMQEYIQDAMTYVRAYGRPDLFI :  498

                           *       520         *       540         *       560         *       580         *       600
C._vestalis     : TFTCNPNWDEIKNLLLSGQTSMHRHDITARVFKQKLKSLMNLITHYSVFCETRCWLYSVEWQKRGLPHAHILIWLVDKVRPEEIDKIISAEIPDPNVDQE :  600
P._machaon      : TFTCNPNWDEIKNLLLSGQTSMHRHDITARVFKQKLKSLMNLITHYSVFGETRCWLYSVEWQKRGLPHAHILIWLVDKVRPEEIDKIISAEIPDPNVDQE :  600
A._rosae        : TFTCNPNWDEIKNLLLSGQTSMHRHDITARVFKQKLKSLMNLITHYSVFGETRCWLYSVEWQKRGLPHAHILIWLVDKVRPEEIDKIISAEIPDPNVDQE :  600
P._tepidariorum : TFTCNPNWAEIKNLLLSGQTSMHRHDIIARVLKQKLKSLMKLITHHSVFGEARCWLYSVEWQKRGLPHAHILIWLVDKVRPEEIDKIISAEIPNPNADQE :  598

                           *       620         *       640         *       660         *       680         *       700
C._vestalis     : LFNIVTTNMIHGPCGTLNMMSPCMNNGKCKKRFPKPCQTDTITNIDGYPSYRRRDVDNGGQSYELRLSNGVRVDIDNRWVVPYSPLLCKTYKAHINVELC :  700
P._machaon      : LFNIVTTNMIHGPCGTLNMMSPCMNDGKCTKRFPKPCQTDTITNIDGYPSYRRRDVDNGGQSYELRLSNGVRVDIDNRWVVPYSPLLCKTYKAHINVELC :  700
A._rosae        : LFNIVTTNMIHGPCGTLNMMSPCMNDGKCTKRFPKPCQTDTITNIDGYPSYRRRDVDNGGQSYELRLSNGVRVDIDNRWVVPYSPLLCKTYKAHINVELC :  700
P._tepidariorum : LFDIVTTNMIHGPCGSLNMMSPCMDNGKCTKRFLKPCQTDTISNIDGYPSYRRRDVDNGGQSYELRLSNGVRVDIDSRWVVPYSPLLCKTYKAHMNVELC :  698

                           *       720         *       740         *       760         *       780         *       800
C._vestalis     : SSVKSIKYICKYVHKGSDEAIFAVQNVNDNDEITRYQMGRYISSNEAVWRIFTFPLHERDPAVIHLAVHLENGQRVYFTEQTALQQALTAPKTTLTEFFN :  800
P._machaon      : SSVKSIKYICKYVHKGSDKAIFAVQNVNDNDEITRYQMGRYISSNEAVWRIFTFPIHERDPAVIHLAVHLENGQRVYFTEQTALQQALTAPKTTLTEFFN :  800
A._rosae        : SSVKSIKYICKYVHKGSDKAIFAVQNVNDNDEITRYQMGRYISSNEAVWRIFTFPIHERDPAVIHLAVHLENGQRVYFPEQTALQQALTAPKTTLTEFFN :  800
P._tepidariorum : SSVKSIKYICKYVHKGRDKAIFAVQSVNKNDEITHHQMGRYISSNEAIWRIFTFPIHERDPAVVHLAVHLENGQRVYFTEQNALQQALTAPKTTLTEFFN :  798

                           *       820         *       840         *       860         *       880         *       900
C._vestalis     : LGNRQDVVGQFAKTLMYTDVPKFFTWNEQSKNWEPRKRGIPIPGFADIFMTNTLGRLYTVHPKQRKCFFLRLLLVNVPGPTSFQYLRKVNGTLYDSFFDA :  900
P._machaon      : LCNRQDVVGQFAKTLMYTDVPKFFTWNKQSKNWEPRKRGIPVTGFADIFMTNTLGRLYTVHPKQRECFFLRLLLVNVLGPTSFQYLRKVNGILYDTFFDA :  900
A._rosae        : LCNRQDVVGQFAKTLMYTDVPKFFTWNKQSKNWEPRKRGIPVPGFADIFMTNTLGRLYTVHPKQRECFFLRLLLVNVLGPTSFQYLRKVNGILYDTFFDA :  900
P._tepidariorum : LCNRQDIVGEFTKALMYTDVPKFFTWNKQSKSWEPRKRGTPVPGFADIVMTNTLGRLYTVLPKQRECFFLRLLLVNVPGPTSFQYLRKVNGALYGTLFDA :  898

                           *       920         *       940         *       960         *       980         *      1000
C._vestalis     : CRELHLLDDDNHWDLTLADAALSSSPQQIRQLFSIILTTCFPSEASALWNKYKDSMSEDILHRIRITNQNPDIQFSAEIYNEALIMIEDICILISNMPLI : 1000
P._machaon      : CRELHLLEDDNHWDLTLADAALSSSPQQIRQLFSIILTTCFPSEASALWNKYKDSMSEDILHRIRITNQNPDIQFSAEIYNEALIMIEDICILISNMPLI : 1000
A._rosae        : CRELHLLEDDNHWDLTLADAALSSSPQQIRQLFSIILTTCFPSEASALWNKYKDSMSEDILHRIRITNQNPDSQFSAEIYNEALIMIEDICILISNMPLI : 1000
P._tepidariorum : CRELHLLEDDNHWDLTLADAALSSSPQQIRQLFSIILTTCFPSEASALWNKYKDSMSEDILYRIRITNQNLCLEFSAEIYNEALIMIEDICFLISNMPLM :  998

                           *      1020         *      1040         *      1060         *      1080         *      1100
C._vestalis     : HFGMPAPNRPAVDIINSDVQREHQFDKTSLATFVANNEQLLTAEQRNVYDQINVSIVPQQGGFFFLDAPGGTGKTFLIALILARIRSQNHMALAIASSGI : 1100
P._machaon      : HFGMPAPNRPAVDIINSDVQREHQFDKTSLATFVANNEQLLTAEQRNVYDQINVSIAAQQGGFFFLDAPGGTGKTFLISLILARIRSQNHIALAIASSGI : 1100
A._rosae        : HFGMPAPNRPAVDIIDSDVQREHQFDKTSLATFVANNEQLLTAEQRNVYDRINVSIAAQKGGFFFLDAPGGTGKTFLISLILARIRSQNHIALAIASSGI : 1100
P._tepidariorum : NFGMPAPSSR-----YHQQRREHQFDMTYLATFVANNERLLTAEQRN--DQINVSIAAQQGGFFSLDAPGGTGKTFLISLILALIRSQNHIALAIASSGI : 1091

                           *      1120         *      1140         *      1160         *      1180         *      1200
C._vestalis     : AATLLDGGRTAHSALKLP-LNVHTNPEAMCNMHKHSGMAEVLRKCKIIIWDECTMAHKHPLEALDRSLKDIKNNARLFGGALLLLSGDFRQTLPVIPRAT : 1199
P._machaon      : AATLLDGGRTAHSALKLP-LNVHTNPEAMCNIKKHSGMAEVLRKCKIIIWDECTMAHKHSLEALDRSLKDIKDNTRLFGGALLLLSGDFRQTLPVIPRAT : 1199
A._rosae        : AATLLDGGRTAHSALKLPLLNVHTNPEAMCNIKKHSGMAEVLRKCKIIIWDECTMAHKHSLEALDRSLKDIKDNTRLFGGALLLLSGDFRQTLPVIPRAT : 1200
P._tepidariorum : AATLLDGGRTAHSALKLP-LNIHTNPEAMCNIKKHSGMAEVLRKCKIIIWDECTMAHKHSLEALDRSLKDIKDNTRLFGGALLLLSGDFRQTLPVIPRAT : 1190

                           *      1220         *      1240         *      1260         *      1280         *      1300
C._vestalis     : YADEINACLKESYLWPSVTKLCLTINMRVRLQNDPLASGFSEKLLDIGNGKIQLYENTQYIRLPENFRNMVATKDELITSIFPDLRHNYTNHEWLRERAI : 1299
P._machaon      : YADEVNACLKESYLWRSVSKLSLTINMRVQLQNDPLASGFSEQLLDIGNGKIQLYEDTQFIKFPENFCNMVATKDELINSIFPDLRYNYTNHEWLRERAI : 1299
A._rosae        : YADEVNACLKESYLWRSVSKLSLTINMRVQLQNDPLASGFSEQLLDIGNGKIQLYENTQFIKFPENFCNMVATKDELINSIFPDLRYNYTHHEWLRERAI : 1300
P._tepidariorum : YADEINACLKESYLWRSVSKLCLTLNMRVQLQNDPLASRFSEQLLDIGNGKIQLYEDTQYIRLPQNFCNMVPTKEELIKSIFPDLPHNYTNHAWLRERAI : 1290

                           *      1320         *      1340         *      1360         *      1380         *      1400
C._vestalis     : LAATNLDVDAINFKIQQSLPGDEITFKSIDTVVDPDEVVNYSEEFLNSLDLPGMPPHNLRLKTGSPIILLRNLNASKLCNGTRLVVKKIMGNILEATILG : 1399
P._machaon      : LAAKNLDVDAINFKIQQSLPGNEITFKSIDTVVDPDEVVNYPVEFLNSLDLPGMPPHNLRLKIGSPIILLRNLNAPRLCNGTRLVIKKIMGNIIEANILS : 1399
A._rosae        : LAAKNLDVDAINFKIQQSLPGNEITFKSIDTVVNPDEVVNYPVEFLNSLDLPGMPPHNLRLKIGSPIILLRNLNAPRLCNGTRLVIKKIMGNIIEANILS : 1400
P._tepidariorum : LAAKNLDVDAINFKVQQSLPGSEITFKSIDTVVNPDEVVNYPAEFLNSLDLPGMPPHNLRLKIDSPIILLRNLNAPKLCNGTRLVIKKIMGNILEATILS : 1390

                           *      1420         *      1440         *      1460         *      1480         *
C._vestalis     : GKFQGEVVLLPRIPMIPSDSPIPFKRFQFPIRLAYAMTINKSQGQTMTICGLDLENPCFSHGQLYVACSRVGKPSNLFIYTPQGLTKNIVHPMALR : 1495
P._machaon      : GKFQGEIVLLPRIPMIPSDSPIPFKRLQFPIRLAYAMTINKSQGQTMTFCGLDLENPCFSHGQLYVACSRVGKPSSLFVYTPHGLTKNIVHPMALR : 1495
A._rosae        : GKFQGEIVLLPRIPMIPSDSPITFKRLQFPIRLAYAMTINKSQGQTMTFCGLDLENPCFSHGQLYVACSRVGKPSSLFVYTSHGLTKNIVHPMALR : 1496
P._tepidariorum : GKFKGEVVLIPRIPMIPSDSPIPFKRLQFPILLAFDMTINKSQGQTMTICGLDLENPCLSHGQSYIACSRVGKPSNLFAYAPEGLTKNIVHLMALR : 1486
```

**b**

| | *C. vestalis* | *P. machaon* | *A. rosae* | *P. tepidariorum* |
|---|---|---|---|---|
| *C. vestalis* | | | | |
| *P. machaon* | 0.041472 | | | |
| *A. rosae* | 0.054181 | 0.015385 | | |
| *P. tepidariorum* | 0.124495 | 0.106999 | 0.11642 | |

Figure S7

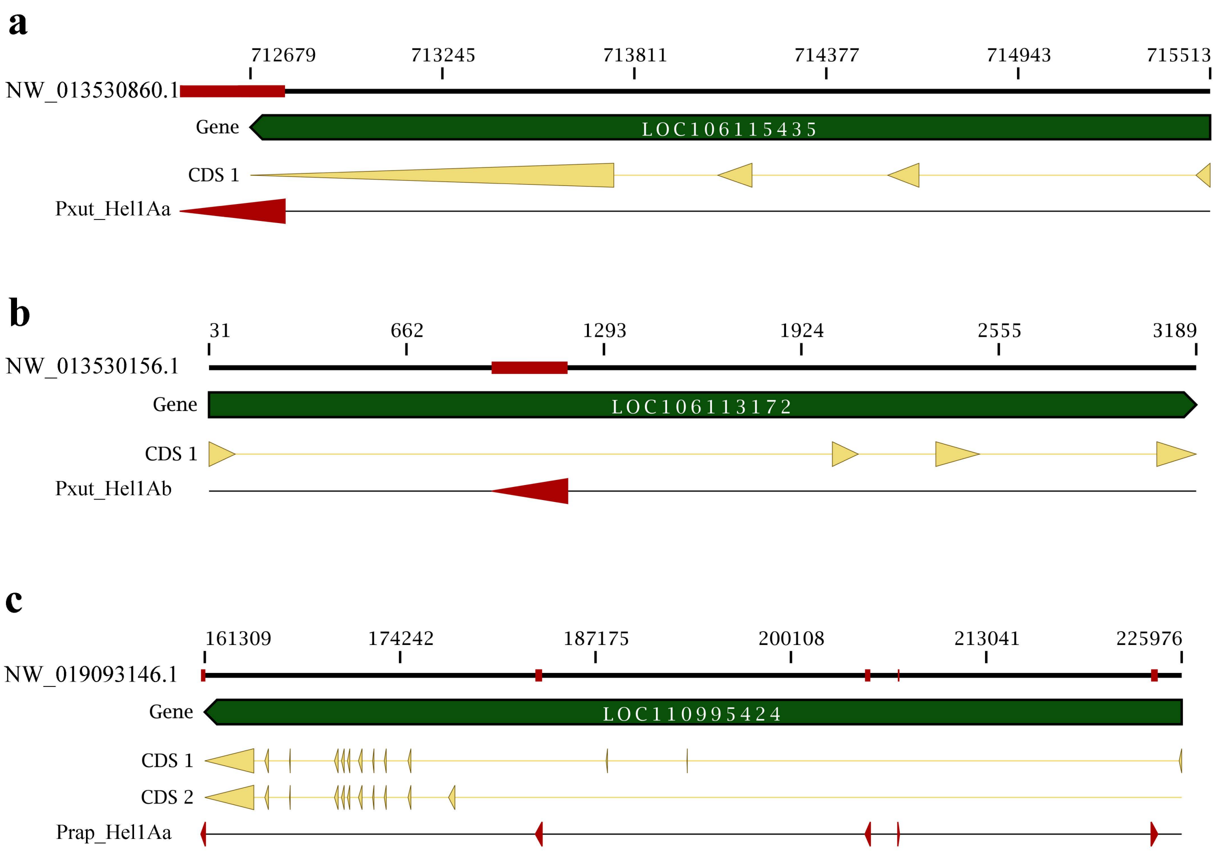
a
712679
713245
713811
714377
714943
715513
NW_013530860.1
Gene
LOC106115435
CDS 1
Pxut_Hel1Aa
b
31
662
1293
1924
2555
3189
NW_013530156.1
Gene
LOC106113172
CDS 1
Pxut_Hel1Ab
c
161309
174242
187175
200108
213041
225976
NW_019093146.1
Gene
LOC110995424
CDS 1
CDS 2
Prap_Hel1Aa

Figure S8

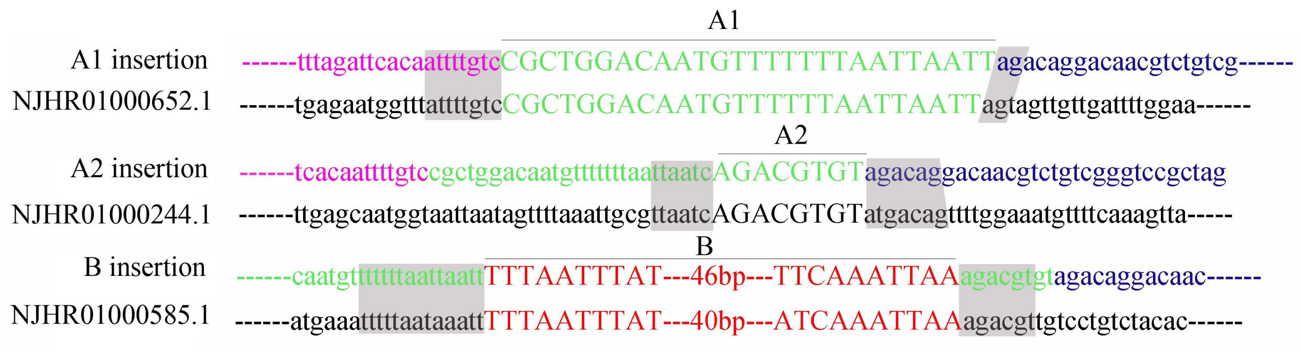
A1
A1 insertion
------tttagattcacaattttgtcCGCTGGACAATGTTTTTTTAATTAATTagacaggacaacgtctgtcg------
NJHR01000652.1
------tgagaatggtttattttgtcCGCTGGACAATGTTTTTTTAATTAATTagtagttgttgattttggaa------
A2
A2 insertion
------tcacaattttgtccgctggacaatgtttttttaattaatcAGACGTGTagacaggacaacgtctgtcgggtccgctag
NJHR01000244.1
------ttgagcaatggtaattaatagttttaaattgcgttaatcAGACGTGTatgacagttttggaaatgttttcaaagtta-----
B
B insertion
------caatgtttttttaattaattTTTAATTTAT---46bp---TTCAAATTAAagacgtgtagacaggacaac------
NJHR01000585.1
------atgaaatttttaataaattTTTAATTTAT---40bp---ATCAAATTAAagacgttgtcctgtctacac------

Figure S9

a

```
                                    *        20         *        40         *        60
BBJE01004687.1:58267-58430 : TCTTTATATATATAATTCTTCTGTGAGTGTGTATGTCACTGAACTTCTCTCAAACGACTGGACCG :  65
Pxut_Hel1Aa                : TCTTTATATATATAATTCTTCTGTGAGTGTGTATGTCACTGAACTTCTCTCAAACGACTGGACCG :  65
Pxut_Hel1Ab                : TCTTTATATATATAATTCTTCTGTGAGTGTGTATGTCACTGAACTTCTCTCAAACGACTGGACCG :  65
                             TCTTTATATATATAATTCTTCTGTGAGTGTGTATGTCACTGAACTTCTCTCAAACGACTGGACCG

                                  *       80         *       100         *       120         *
BBJE01004687.1:58267-58430 : ATTTTGATGAATTTTTTTGTGTGTGTTCAAGGGGATCTGGGAATGGTTTAGATTCACAATTTT-- : 128
Pxut_Hel1Aa                : ATTTTGATGAAATTTTTTGTGTGTGTTCAAGGGGATCTGGGAATGGTTTAGATTCACAATTTTGT : 130
Pxut_Hel1Ab                : ATTTTGATGAAATTTTTTGTGTGTGTTCAAGGGGATCTGGGAATGGTTTAGATTCACAATTTT-- : 128
                             ATTTTGATGAAaTTTTTTGTGTGTGTTCAAGGGGATCTGGGAATGGTTTAGATTCACAATTTT

                                     140         *       160         *       180         *
BBJE01004687.1:58267-58430 : ----------------------------------------------------------------- :   -
Pxut_Hel1Aa                : CCGCTGGACAATGTTTTTTTAATTAATTTTTAATTTATTAGTAGTTGTTGATTTTGGAATGTTTT : 195
Pxut_Hel1Ab                : --------------------CATCAAATATTAACTTCATATTAATTT------------------ : 155
                                                 at aa t ttaa tt  ta ta tt

                                 200         *       220         *       240         *     260
BBJE01004687.1:58267-58430 : -----------------------------------------------------ACGTGTAGACAG : 140
Pxut_Hel1Aa                : ACATTGGATCCGACAGACGGCGCTACCATCGCAGTGTCAAATTTTAATTAAAGACGTGTAGACAG : 260
Pxut_Hel1Ab                : ----------------------------------------ATTCAAATTAAAGACGTGTAGACAG : 180
                                                                     att  aattaaagACGTGTAGACAG

                                     *       280
BBJE01004687.1:58267-58430 : GACAACGTCTGTCGGGTCCGCTAG : 164
Pxut_Hel1Aa                : GACAACGTCTGTCGGGTCCGCTAG : 284
Pxut_Hel1Ab                : GACAACGTCTGTCGGGTCCGCTAG : 204
                             GACAACGTCTGTCGGGTCCGCTAG
```

b

```
                                         *        20         *        40         *        60
BBJE01004687.1:58267-58430   : TCTTTATATATAT-AATTCTTCTGTGAGTGTGTATGTCACTGAACTTCTCTCAAACGACTGGACC :  64
FJUZ01003913.1:464817-464657 : TCTTTATATATATAAATTCTTCTGTAAGTGTGTATGTCACTGAACTTCTCTTAAACGACTGGACC :  65
Csup_Hel1Aa                  : TCTTTATATATAT-AATTCTTCTGTGAGTGTGTATGTCACTGAACTTCTCTTAAACGACTGGACC :  64
Csup_Hel1Ab                  : TCTTTATATATAT-AATTCTTCTGTGAGTGTGTATGTCACTGAACTTCTCTTAAACGACTGGACC :  64
                               TCTTTATATATAT AATTCTTCTGTgAGTGTGTATGTCACTGAACTTCTCTtAAACGACTGGACC

                                     *       80         *       100         *       120         *
BBJE01004687.1:58267-58430   : GATTTTGATGAATTTTTTTGTGTGTGTTCAAGGGGATCTGGGAATGGTTTAGATTCACAATTTTA : 129
FJUZ01003913.1:464817-464657 : GATTTTGATGAA-TTTTTTGTGTGTGTTCAAGGGGATCTGAGAATGGTTTAGATTCACAATTTTG : 129
Csup_Hel1Aa                  : GATTTTGATGAAATTTTTTGTGTGTGTTCAAGGGGATCTGAGAATGGTTTAGATTCACAAAGACG : 129
Csup_Hel1Ab                  : GATTTTGATGAAATTTTTTGTGTGTGTTCAAGGGGATCTGAGAATGGTTTAGATTCACAATTTTG : 129
                               GATTTTGATGAA TTTTTTGTGTGTGTTCAAGGGGATCTGaGAATGGTTTAGATTCACAAttttg

                                       140         *       160         *       180         *
BBJE01004687.1:58267-58430   : CGTG------------------------------------------------------------- : 133
FJUZ01003913.1:464817-464657 : T---------------------------------------------------------------- : 130
Csup_Hel1Aa                  : TG--------------------------------------------------------------- : 131
Csup_Hel1Ab                  : TCCGCTGGACAATGTTTTTTTAATTAATTTTTAATTTATTAGTAGTTGTTGATTTTGGAATGTTT : 194
                               t

                                   200         *       220         *       240         *
BBJE01004687.1:58267-58430   : --------------------------------TAGACAGGACAACGTCTGTCGGGTCCGCTAG : 164
FJUZ01003913.1:464817-464657 : --------------------------------CAGACAGGACAACGTCTGTCGGGTCCGCTAG : 161
Csup_Hel1Aa                  : --------------------------------TAGACAGGACAACGTCTGTCGGGTCCGCTAG : 162
Csup_Hel1Ab                  : TACATTGGATCCGACAGACGGCGCTACCATACTAGACAGGACAACGTCTGTCGGGTCCGCTAG : 257
                                                               tAGACAGGACAACGTCTGTCGGGTCCGCTAG
```

c

```
                                         *        20         *        40         *        60         *
ANCD01086587.1:1160-1321 : TCTTTATATATACAATTCTTCTGTGAGTGTGTATGTCACTGAACTTCTCTTAAACGACTGGACCGATTTT :  70
ANCD01202355.1:2392-2551 : --TTTATATATGTAATTCTTCTGTGAGTGTGTATGTCACTGAACTTCTCTTAAACGACTGGACCGATTTT :  68
ANCD01057513.1:339-497   : TCTTTATATATATAATTCTTCTGTGAGTGTGTATGTCACTGAACTTCTCTTAAACGACTGGACCGATTTT :  70
ANCD01022843.1:2146-2308 : TCTTTATATATATAATTCTTCTGTGAGTGTGTATGTCACTGAACTTCTCTTAAACGACTGGACCGATTTT :  70
ANCD01076528.1:2386-2547 : -CTTTATATATATAATTCTTCTGTGAGTGTGTATGTCACTGAACTTCTCTTAAACGACTGGACCGATTTT :  69
Csup_Hel1Ea              : TCTTTATATATATAATTCTTCTGTGAGTGTGTATGTCACTGAACTTCTCTTAAACGACTGGACCGATTTT :  70
                            cTTTATATATatAATTCTTCTGTGAGTGTGTATGTCACTGAACTTCTCTTAAACGACTGGACCGATTTT

                                  80         *       100         *       120         *       140
ANCD01086587.1:1160-1321 : GATGAAATTTTTTGTGTGTGTTCAAGGGGATCTGAGAATGGTTTAGATTCACAA---------------- : 124
ANCD01202355.1:2392-2551 : GATGAAATTTTTTGTGTGTGTTCAAGGGGATCTGAGAATGGTTTAGATTCACAA---------------- : 122
ANCD01057513.1:339-497   : GATGAAATTTTTTGTGTGTGTTCAAGGGGATCTGAGAATGGTTTAGATTCA------------------- : 121
ANCD01022843.1:2146-2308 : GATGAAATTTTTTGTGTGTGTTCAAGGGGATCTGAGAATGGTTTAGATTCACAAT--------------- : 125
ANCD01076528.1:2386-2547 : GATGAAATTTTTTGTGTGTGTTCAAGGGGATCTGAGAATGGTTTAGATTCACAAT--------------- : 124
Csup_Hel1Ea              : GATGAAATTTTTTGTGTGTGTTCAAGGGGATCTGAGAATGGTTTAGATTCACAATTTTGTCCGCTGGACA : 140
                           GATGAAATTTTTTGTGTGTGTTCAAGGGGATCTGAGAATGGTTTAGATTCAcaa

                                   *       160         *       180         *
ANCD01086587.1:1160-1321 : -----------------TTTTTTTATGGCGGTACGAAGTTCGCTGGGACAGCTAG : 162
ANCD01202355.1:2392-2551 : -----------------TTTTTTTAGGGCGGTACGAAGTTCGCTGGGTCAGCTAG : 160
ANCD01057513.1:339-497   : -----------------TTTTTTTATGGCGGTACGAAGTTCGCTGGGACAGCTAG : 159
ANCD01022843.1:2146-2308 : -----------------TTTTTTTATGGCGGTACGAAGTTCGCTGCGACAGCTAG : 163
ANCD01076528.1:2386-2547 : -----------------TTTTTTTATGGCGGAACGAAGTTCGCTGGGACAGCTAG : 162
Csup_Hel1Ea              : ATGTTTTTTTAATTAATTTTTTTTATGGCGGTACGAAGTTCGCTGGGTCAGCTAG : 195
                                            TTTTTTTAtGGCGGtACGAAGTTCGCTGgG CAGCTAG
```

Figure S10

**Prap_Hel1Aa**

| | |
|---|---|
| LWME01000121.1 184392-184897 | ACGGGTAAATTAACAAATTAATGAAAATAA/**Prap_Hel1Aa**/TATACATATATAAATATATATTTATTCATT |
| JZSA01005098.1 999-1058 | AAGGATAAATTAACAAATTAATGAAAATAA-------------GATACATATATAAATATATATTAATTCATT |
| ANCD01212189.1 161-102 | AAGGGTTAATTAACAAGTCAATGAAAATGA-------------TATACATATATAAATATATATTTATTCATT |

| | |
|---|---|
| LWME01000121.1 184392-184897 | ACGGGTAAATTAACAAATTAATGAAAATAA/**Prap_Hel1Aa**/TATACATATATAAATATATATTTATTCATT |
| JZSA01001834.1 1308-1249 | AAGGGTAAATTAACAAATTAATGAAAATAA-------------TATACATGTATAAATATATATTTATTCATT |

**Cves_Hel1Aa**

| | |
|---|---|
| LWME01000373.1 638382-638441 | CTAAAGGCCATCCCTGCAACTTCCCGCTAA-------------TTCCATTCCTGGGCGCTTAAAATTGTACTT |
| LWME01000063.1 1885-1826 | CTAAAGGCTATCCCTGCAACTTCCCGCTAA-------------TTCCATTCCTGGGCGCTTAAAATTGTACTT |
| JZSA01007367.1 22473-22810 | CTAAAAGCCATCACTGCAACTTCCTGCTAA/**Cves_Hel1Aa**/TTCCATATCTAAGCGCTTAAAATTGAACTT |
| JZSA01006456.1 1015-956 | CTAAAGGCCATCCGTGCAACTTCCCGCTAA-------------TTCCATTCCTGGGCGCTTAAAATTGTACTT |
| JZSA01003430.1 2650226443 | CTAAAGGCCAGCCCTGCAACTTCCCGCTAA-------------TTCCATTCCTGGGCGCTTAAAATTGTACTT |
| JZSA01002828.1 55690-55749 | CTAAAGGCCATCCGTGCAACTTCCCGCTAA-------------TTCCATTCCTGGGCGCTTAAAATTGTACTT |
| JZSA01000944.1 4265-4324 | CTAAAGGCCATCCGTGCAACTTCCCGCTAA-------------TTCCATTCCTGGGCGCTTAAAATTGTACTT |
| JZSA01007432.1 13875-13817 | CTAAAGGCCATCCCT-CAACTTCCCGCTAA-------------TTCCATTCCTGGGCGCTTAAAATTGTACTT |

| | |
|---|---|
| JZSA01007367.1 22473-22810 | CTAAAAGCCATCACTGCAACTTCCTGCTAA/**Cves_Hel1Aa**/TTCCATATCTAAGCGCTTAAAATTGAACTT |
| AZMT02018341.1 4720-4778 | CTGAAGGCCATCCCTGTAACTTCCCGCTAA-------------TTCCTTACTTAGGTACTT-AAATTGTACTT |

**Csup_Hel1Aa**

| | |
|---|---|
| ANCD01041172.1 2033-1818 | CCATCCCGGACAAAGAATGTGATGCGTAAT/**Csup_Hel1Aa**/TATATATATATTAAGAT-AATAATA----- |
| LWME01000611.1 316533-316590 | CCATTGAGGACAAACATTGTGACACGAAAT-------------T-TATATATATTAAGATTAAGA-TAAGTAA |

**Mcin_Hel1Ca**

| | |
|---|---|
| APLT01004244.1 41865-42274 | AAATGCATATAGATGTATACACGGTACATA/**Mcon_Hel1Ca**/TACCAAAATAAC-TTTTTTACAATTTTTGT |
| JXPT01029385.1 2455-2396 | AAATGCATATCGATGAACTCACGGTACATA-------------TACCAAAATAACATTTTTTACAATTTTTGT |

Figure S11

a

```
                         *        20         *        40         *        60         *        80         *       100
Cv_calreticulin : ATGCGA--GCTCTCGTT----GCTGTC------CTGGCAATTGCCGTAATTGCCACCGTGTCTGC--TGAAGTTTCTTCGAAGAAAGATTTCAGACGA :   86
Pr calreticulin : ATGAAATCGTTTTATTAGGAGTTGTCAGCTTGCTGGCAATT-----------TATTCTGTCAACTGTGAAGTGTTTTACGAAGAAAGGTTCCTAGATGA :   89
                  ATG  A  G T T  TT    G TGTC      CTGGCAATT           A   TGTC  C  TGAAGT TT T CGAAGAAAG TT    AGA GA

                         *       120         *       140         *       160         *       180         *       200
Cv_calreticulin : TTCATGGGAAAGAACTGGGTATACTCAGAGCATCCCGGCAAAGAATTCGGAAAGTTCAAGCGGACTGCTGGAAAATTCTACCACGAAGAATTACAAGAC :  186
Pr calreticulin : CTCATGGGAATCGAAGTGGGTGTACAGTGAACACCCTGGTAAAGAATTCGGCAAATTCAAGTTAACTGCTGGAAAGTTCTACAATGACCCCGAAGAAGAT :  189
                   TCATGGGAA  GAA TGGGT TAC    GA CA CC GG AAAGAATTCGG AA TTCAAG   ACTGCTGGAAA TTCTAC A GA       A AAGA

                         *       220         *       240         *       260         *       280         *       300
Cv_calreticulin : ACAGGTATTCAAACAACTGAAGATGCCAGGTTCTACGCTCTCAGCAGAAAGTTCAAGCCATTCACGAATAAGGACAAGCCATTGGTTGTCCAATTCACCG :  286
Pr calreticulin : AAAGGCCTTCAAACTTCTGAGGATGCCAGATTCTATGCATTGTCCACAAAGTTTAAGCCCTTCTCAAATGAAGGCAAGCCCTTAGTAATCCAGTTCTCAG :  289
                  A AGG  TTCAAAC  CTGA GATGCCAG TTCTA GC  T   CA AAAGTT AAGCC TTC C AAT A G CAAGCC TT GT  TCCA TTC C G

                         *       320         *       340         *       360         *       380         *       400
Cv_calreticulin : TCAAGCATGAACAAATGATTGATTGCGGTGGTGGCTACGTGAAAGTGTTCGACTGTTCATTGGACCAGAAGGGCATGCACGGAGAGACACCATACCTTCT :  386
Pr calreticulin : TAAAACATGAGCAAGACATTGATTGTGGAGGTGGTTACGCGAAGGTGTTTGACTGCAAGTTGGAGTCAAAGGACATGCATGGTGAGACACCATATGAAAT :  389
                  T AA CATGA CAA   ATTGATTG GG GGTGG TACG GAA GTGTT GACTG     TTGGA     AAGG CATGCA GG GAGACACCATA     T

                         *       420         *       440         *       460         *       480         *       500
Cv_calreticulin : TATGTTTGGACCTGACATTTGTGGACCTGGAACCAAGAAAGTTCACGTAATCTTTAACTACAAGGGAAAGAATCTGTTGATCAAGAAAGACATTCGTTGC :  486
Pr calreticulin : CATGTTTGGGCCTGACATCTGCGGTCCCGGTACCAAAAAGGTGCATGTTATCTTCAGCTACAAGGGCAAGAACCACCTCATCAAGAAAGACATCCGTTGC :  489
                   ATGTTTGG CCTGACAT TG GG CC GG ACCAA AA GT CA GT ATCTT A CTACAAGGG AAGAA C   T ATCAAGAAAGACAT CGTTGC

                         *       520         *       540         *       560         *       580         *       600
Cv_calreticulin : AAAGACGACGTATACACTCACTTGTACACTTTGATTGTCAAACCAGACAACACTTACGAGGTTCTTATCGACAATGAAAAGGTAGAGTCTGGAGAGCTTG :  586
Pr calreticulin : AAGGATGATGTATACACTCACTTGTACACTCTTATTGTGAACCCTGACAACACCTATGAAGTTCTCATTGATAATGAGAAGGTGGAATCTGGTAGCCTTG :  589
                  AA GA GA GTATACACTCACTTGTACACT T ATTGT AA CC GACAACAC TA GA GTTCT AT GA AATGA AAGGT GA TCTGG    CTTG

                         *       620         *       640         *       660         *       680         *       700
Cv_calreticulin : AAGCCGACTGGGACTTTTTGCCACCCAAGAAAATCAAGGACCCGAATGAGAAGAAACCCGAAGACTGGGATGACCGTGCCACCATTCCCGATCCAGAAGA :  686
Pr calreticulin : AAGATGACTGGGACTTCTTACCTGCTAAGAAGATCAAGGACCCATCAGCCAAAAAGCCCGAAGACTGGGATGAGAGAGCAACCATTCCTGACCCAGATGA :  689
                  AAG  GACTGGGACTT TT CC  C AAGAA ATCAAGGACCC    G  AA AA CCCGAAGACTGGGATGA  G GC ACCATTCC GA CCAGA GA

                         *       720         *       740         *       760         *       780         *       800
Cv_calreticulin : CACCAAACCCGAAGACTGGGACAAGCCTGAGACTATCCCCGGCCCCGAAGCCACCAAACCAGAAGATTGGGACGATGAAATGGACGGAGAATGGGAACCA :  786
Pr calreticulin : CAAAAAGCCAGAAGACTGGGAAAAGCCAGAACACATTCCAGACCCTGATGCCACCAAACCTGAAGATTGGGACGATGAAATGGATGGAGAATGGGAACCA :  789
                  CA  AA CC GAAGACTGGGA AAGCC GA    AT CC G CCC GA GCCACCAAACC GAAGATTGGGACGATGAAATGGA GGAGAATGGGAACCA

                         *       820         *       840         *       860         *       880         *       900
Cv_calreticulin : CCAATGATCGACAACCCAGACTTCAAAGGTGAATGGAAGCCAAAGCAAATTGATAACCCAGCTTACAAGGGACCATGGCTCCATCCTGAAATTGACAACC :  886
Pr calreticulin : CCAATGATTGACAACCCTGAATACAAGGGAGTCTGGGCTCCTAAGCAGATTGACAACCCCGCATACAAAGGTCCCTGGGTACACCCAGAAATTGATAACC :  889
                  CCAATGAT GACAACCC GA T CAA GG G  TGG   CC AAGCA ATTGA AACCC GC TACAA GG CC TGG T CA CC GAAATTGA AACC

                         *       920         *       940         *       960         *       980         *      1000
Cv_calreticulin : CTGAGTACGTTAAGGATGAAGAACTCTACAAGAAAGACGAAGTCTGCGCCATTGGTTTCGATCTTTGGCAAGTCCAGTCTGGAACCATCTTTGACAACGT :  986
Pr calreticulin : CAGAATACACAGCAGACCCAACTCTGTACAAGCGAGATGAACTTTGCGCAGTCGGTCTGGACTTATGGCAGGTCAAATCTGGAACCATTTTCGACAACTT :  989
                  C GA TAC      GA   A   CT TACAAG  AGA GAA T TGCGC  T GGT T GA  T TGGCA GTC A TCTGGAACCAT TT GACAAC T

                         *      1020         *      1040         *      1060         *      1080         *      1100
Cv_calreticulin : TCTCATTACTGATGAGCCAGAAGCCGCCAGCAAATTCGCCGAAGATGTTTGGAAACCAAACTTTGAAGGTGAAAAGAAAATGAAAGAAGCTCAAGATGGG : 1086
Pr calreticulin : CCTGTTCACTGATGACCCAGCTGCAGCAAAGGAGAGAGGAGAAGAGATTAAGAA---GAAGATTGAGGGAGAGAAAAAAATGAAGAATGAGCAGGATGAA : 1086
                   CT  T ACTGATGA CCAG  GC GC A   A    G  GAAGA  TT  GAA     AA  TTGA GG GA AA AAAATGAA  A G  CA GATG

                         *      1120         *      1140         *      1160         *      1180         *      1200
Cv_calreticulin : GCTGAAAGAAAATCAATGGAGGCGGAGGAACC--AGAAGCCCCAGAAGAGGATGATGATGAAGATGATGATGATGCGGATGACGAAGACAACACCGTACC : 1184
Pr calreticulin : GCGGAGAG---------GAAGAAGGAAAAATCTGAGAAGCC-----------TGAGGATGAAGATGATGAGGATCTTGATGATGAAGCCGCTGAGGGAGC : 1166
```

b

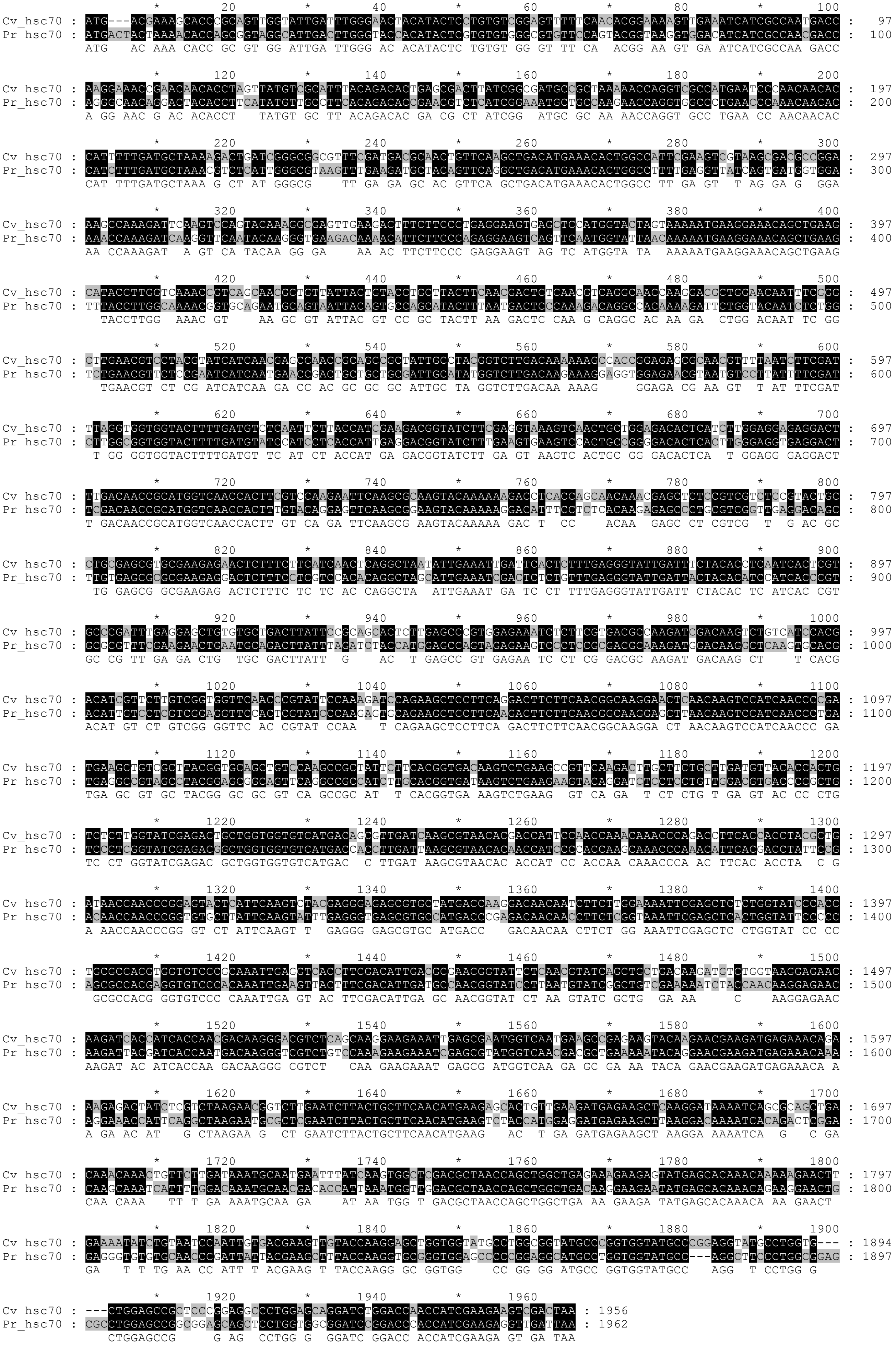

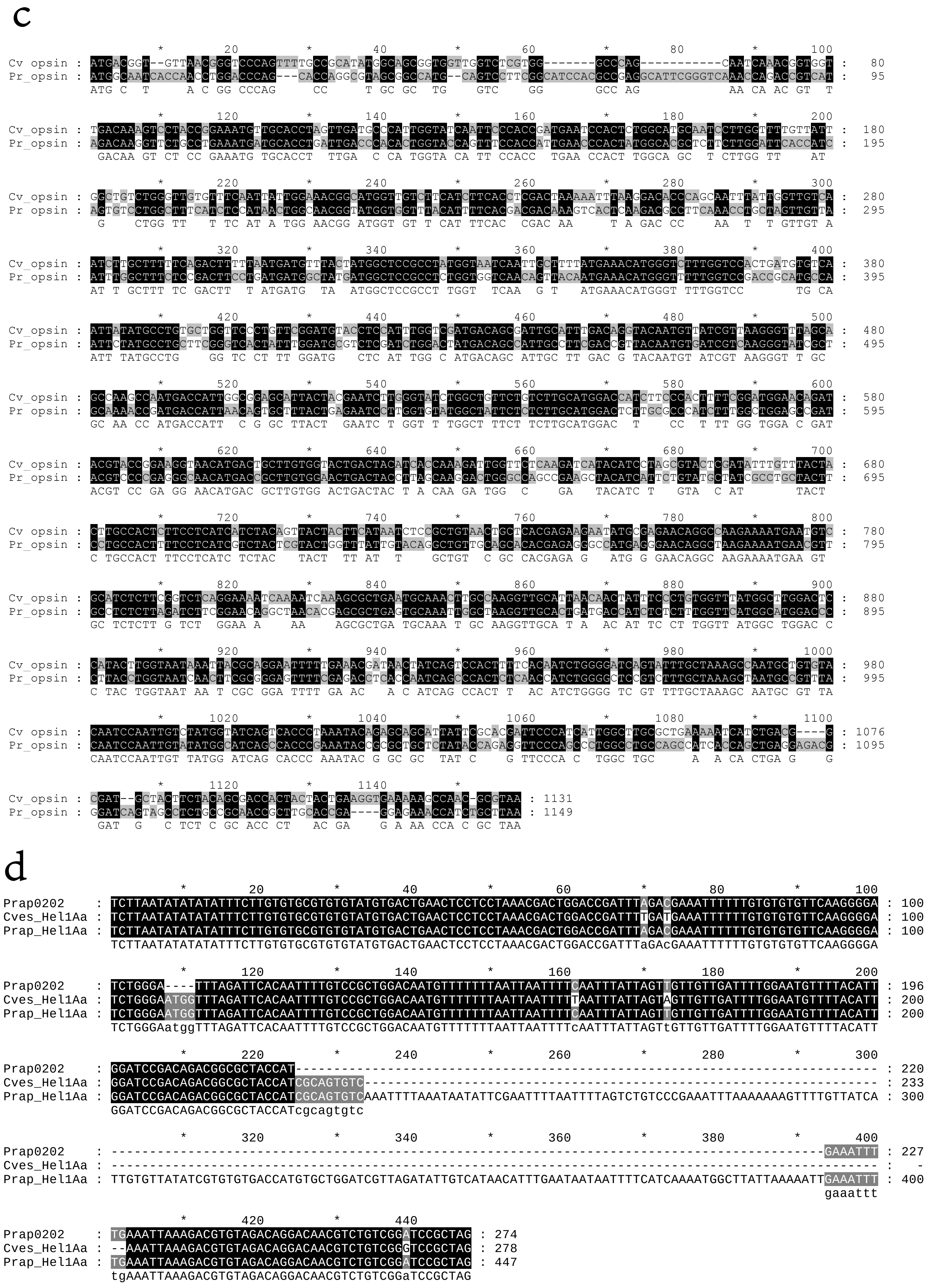
c
Cv_opsin
Pr_opsin
d
Prap0202
Cves_Hel1Aa
Prap_Hel1Aa

e

```
                         *        20         *        40         *        60         *        80         *       100
Cves_Hel1Aa.1 : TCTTAATATATATATTTCTTGTGTGCGTGTGTATGTGACTGAACTCCTCCTAAACGACTGGACCGATTTTGATGAAATTTTTTGTGTGTGTTCAAGGGGA : 100
Cves_Hel1Aa.2 : TCTTAATATATATATTTCTTGTGTGCGTGTGTATGTGACTGAACTCCTCCTAAACGACTGGACCGATTTTGATGAAATTTTTTGTGTGTGTTCAAGGGGA : 100
Cves_Hel1Aa.3 : ----AATATATATATTTCTTGTGTGCGTGTGTATGTGACTGAACTCCTCCTAAACGACTGGACCGATTTTGATGAAATTTTTTGTGTGTGTTCAAGGGGA :  96
Cves_Hel1Aa.4 : TCTTAATATATATATTTCTTGTGTGCGTGTGTATGTGACTGAACTCCTCCTAAACGACTGGACCGATTTTGATGAAATTTTTTGTGTGTGTTCAAGGGGA : 100
Cves_Hel1Aa.5 : -CTTAATATATATATTTCTTGTGTGCGTGTGTATGTGACTGAACTCCTCCTAAACGACTGGACCGATTTTGATGAAATTTTTTGTGTGTGTTCAAGGGGA :  99
Cves_Hel1Aa.6 : TCTTAATATATATATTTCTTGTGTGCGTGTGTATGTGACTGAACTCCTCCTAAACGACTGGACCGATTTTGATGAAATTTTTTGTGTGTGTTCAAGGGGA : 100
Cves_Hel1Aa.7 : -CTTAATATATATATTTCTTGTGTGCGTGTGTATGTGACTGAACTCCTCCTAAACGACTGGACCGATTTTGATGAAATTTTTTGTGTGTGTTCAAGGGGA :  99
Cves_Hel1Aa.8 : TCTTAATATATATATTTCTTGTGTGTGTGTGTATGTGACTGAACTCCTCCTAAACGACTGGACCAATTTTGATGAAATTTTTTGTGTGTGTTCAAGGGGA : 100
Prap0202      : TCTTAATATATATATTTCTTGTGTGCGTGTGTATGTGACTGAACTCCTCCTAAACGACTGGACCGATTTAGACGAAATTTTTTGTGTGTGTTCAAGGGGA : 100
                 cttAATATATATATTTCTTGTGTGcGTGTGTATGTGACTGAACTCCTCCTAAACGACTGGACCgATTTtGAtGAAATTTTTTGTGTGTGTTCAAGGGGA

                         *       120         *       140         *       160         *       180         *       200
Cves_Hel1Aa.1 : TCTGGGAATGGTTTAGATTCACAATTTTGTCCGCTGGACAATGTTTTTTTAATTAATTTTTAATTTATTAGTAGTTGTTGATTTTGGAATGTTTTACATT : 200
Cves_Hel1Aa.2 : TCTGGGAATGGTTTAGATTCACAATTTTGTCCGCTGGACAATGTTTTTTTAATTAATTTTTAATTTATTAGTAGTTGTTGATTTTGGAATGTTTTACATT : 200
Cves_Hel1Aa.3 : TCTGGGAATGGTTTAGATTCACAATTTTGTCCGCTGGACAATGTTTTTTTGATTAATTTTTAATTTATTAGTAGTTGTTGATTTTGGAATGTTTTACATT : 196
Cves_Hel1Aa.4 : TCTGGGAATGGTTTAGATTCACAATTTTGTCCGCTGGACAGTGTTTTTTTAATTAATTTTTAATTTATTAGTAGTTGTTGATTTTGGAATGTTTTACATT : 200
Cves_Hel1Aa.5 : TCTGGGAATGGTTTAGATTCACAATTTTGTCCGCTGGACAATGTTTTTTTAATTAATTTTTAATTTATTAGTAGTTGTTGATTTTGGAATGTTTTACATT : 199
Cves_Hel1Aa.6 : TCTGGGAATGGTTTAGATTCACAATTTTGTCCGCTGGACAATGTTTTTTTAATTAATTTTTAATTTATTAGTAGTTGTTGATTTTGGAATGTTTTACATT : 200
Cves_Hel1Aa.7 : TCTGGGAATGGTTTAGATTCACAATTTTGTCCGCTGGACAATGTTTTTTTAATTAATTTTTAATTTATTAGTAGTTGTTGATTTTGGAATGTTTTACATT : 199
Cves_Hel1Aa.8 : TCTGGGAATGGTTTAGATTCACAATTTTGTCCGCTGGACAATGTTTTTTTAATTAATTTTCAATTTATTAGTAGTTGTTGATTTTGGAATGTTTTACATT : 200
Prap0202      : TCTGGGA----TTTAGATTCACAATTTTGTCCGCTGGACAATGTTTTTTTAATTAATTTTCAATTTATTAGTTGTTGTTGATTTTGGAATGTTTTACATT : 196
                TCTGGGAatggTTTAGATTCACAATTTTGTCCGCTGGACAaTGTTTTTTTaATTAATTTTtAATTTATTAGTaGTTGTTGATTTTGGAATGTTTTACATT

                         *       220         *       240         *       260         *
Cves_Hel1Aa.1 : GGATCCGACAGACGGCGCTACCATCGCAGTGTCAAATTAAAGACGTGTAGACAGGACAACGTCTGTCGGGTACGCTAG : 278
Cves_Hel1Aa.2 : GGATCCGACAGACGGCGCTACCATCGCAGTGTCAAATTAAAGACGTGTAGACAGGACAACGTCTGTCGGGTACGCTAG : 278
Cves_Hel1Aa.3 : GGATCCGACAGACGGCGCTACCATCGCAGTGTCAAATTAAAGACGTGTAGACAGGACAACGTCTGTCGGGTCCGCTAG : 274
Cves_Hel1Aa.4 : GGATCCGACAGACGGCGCTACCATCGCAGTGTCAAATTAAAGACGTGTAGACAGGACAACGTCTGTCGGGTCCGCTA- : 277
Cves_Hel1Aa.5 : GGATCCGACAGACGGCGCTACCATCGCAGTGTCAAATTAAAGACGTGTAGACAGGACAACGTCTGTCGGGTACGCTAG : 277
Cves_Hel1Aa.6 : GGATCCGACAGACGGCGCTACCATCGCAGTGTCAAATTAAAGACGTGTAGACAGGACAACGTCTGTCGGGTACGCTA- : 277
Cves_Hel1Aa.7 : GGATCCGACAGACGGCGCTACCATCGCAGTGTCAAATTAAAGACGTGTAGACAGGACAACGTCTGTCGGGTACGCTAG : 277
Cves_Hel1Aa.8 : GGATCCGACAGACGGCGCTACCATCGCAGTGTCAAATTAAAGACGTGTAGACAGGACAACGTCTGTCGGGTCCGCTA- : 277
Prap0202      : GGATCCGACAGACGGCGCTACCATGAAATTTTGAAATTAAAGACGTGTAGACAGGACAACGTCTGTCGGATCCGCTAG : 274
                GGATCCGACAGACGGCGCTACCATcgcAgTgTcAAATTAAAGACGTGTAGACAGGACAACGTCTGTCGGgT CGCTA
```

Figure S12

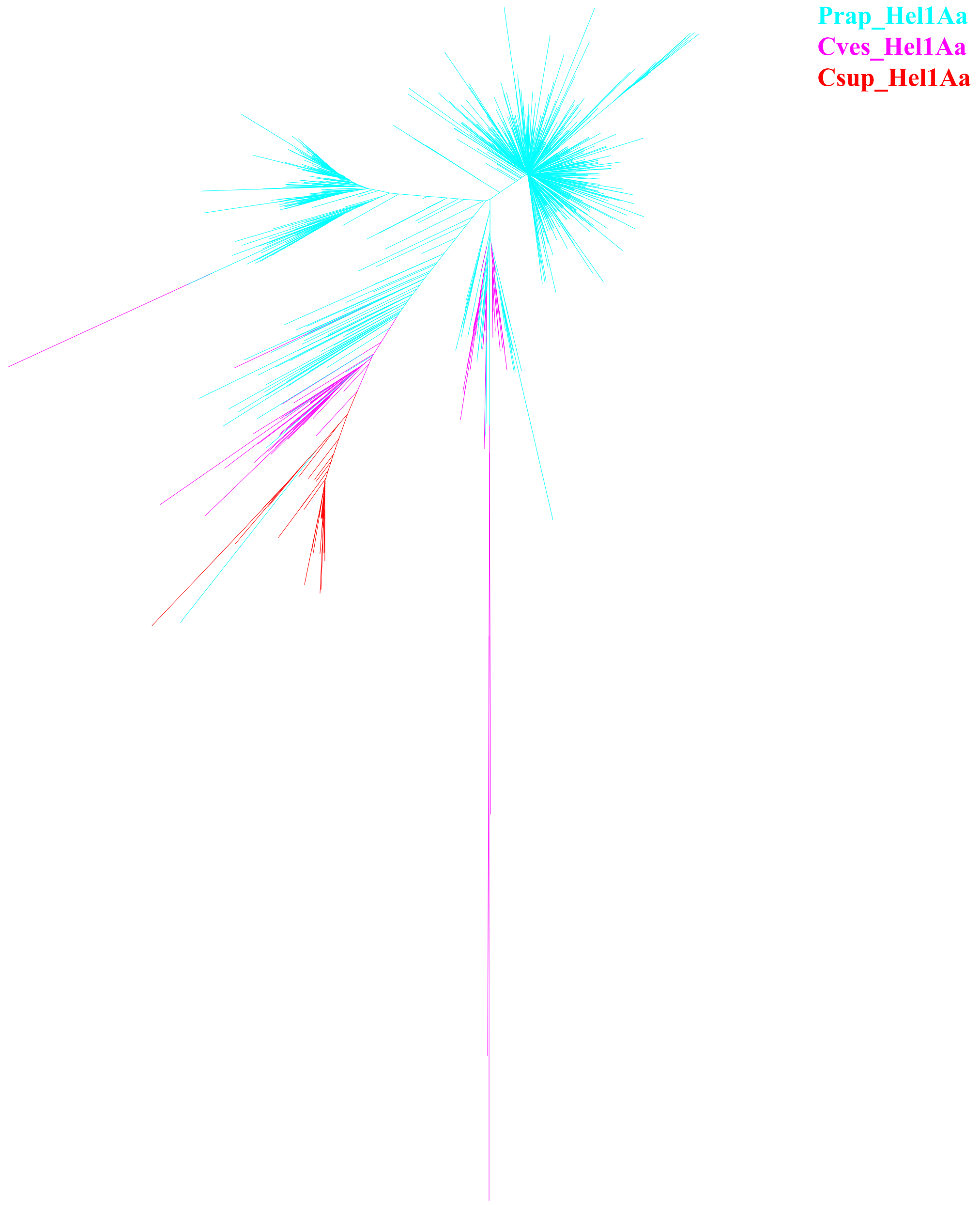


Figure S13

```
                                        *        20         *        40         *        60         *        80         *       100
sequenced                     : GCAAGGCGGCTTCGAGCGAATGTTAAATGTACATAGAAAGAAACTCCATTGGTGCACAGTCGGGGATCGAACCTCAGAGATGTCCTGTGTGATGTAGTGT :  100
LWME01000202.1:139357-138278 : GCAAGGCGGCTTCGAGCGAATGTTAAATGTACATAGAAAGAAACTCCATTGGTGCACAGTCGGGGATCGAACCTCAGAGATGTCCTGTGTGATGTAGTGT :  100
                               GCAAGGCGGCTTCGAGCGAATGTTAAATGTACATAGAAAGAAACTCCATTGGTGCACAGTCGGGGATCGAACCTCAGAGATGTCCTGTGTGATGTAGTGT

                                        *       120         *       140         *       160         *       180         *       200
sequenced                     : GATGTGTGATGTAGACCTGAGAGAGTCGCTCAAGCCACTTGGCCAATAATGCTCACAGACATGCCTCAGTTACACACACAAATACACACCCACACCCTAA :  200
LWME01000202.1:139357-138278 : GATGTGTGATGTAGACCTGAGAGAGTCGCTCAAGCCACTTGGCCAATAATGCTCACAGACATGCCTCAGTTACACACACAAATACACACCCACACCCTAA :  200
                               GATGTGTGATGTAGACCTGAGAGAGTCGCTCAAGCCACTTGGCCAATAATGCTCACAGACATGCCTCAGTTACACACACAAATACACACCCACACCCTAA

                                        *       220         *       240         *       260         *       280         *       300
sequenced                     : ACGTATTCTCTGTATATGTTTAGAAATTACTGTGCAATTTTTAAGTTAAGTTCACTGTACGGTTTTTCTCAATAAATACAATGGCATGTACAATAAATAA :  300
LWME01000202.1:139357-138278 : ACGCATTCTCTGTATATGTTTAGAAATTACTTTGCAATTTTTAAGTTAAGTTCACTGTACGGTTTTTCTCAATAAATACAATGGCATGTACAATAAATAA :  300
                               ACG ATTCTCTGTATATGTTTAGAAATTACT TGCAATTTTTAAGTTAAGTTCACTGTACGGTTTTTCTCAATAAATACAATGGCATGTACAATAAATAA

                                        *       320         *       340         *       360         *       380         *       400
sequenced                     : TAGATTGCATTATTATTACTACATACTTAATTTATTTCTTTAAAAATACATACAAAAATTTACAAACATTATTTATCGTTGTATAAACTTTATTGCAAAT :  400
LWME01000202.1:139357-138278 : TGGCTTGCATTATTATTACTACATACTTAATTTATTTC-TTAAAAATACATACAAAAATTTACAAACATTATTTGTCGTTGTATAAACTTTATTGCAAAT :  399
                               T G TTGCATTATTATTACTACATACTTAATTTATTTC TTAAAAATACATACAAAAATTTACAAACATTATTT TCGTTGTATAAACTTTATTGCAAAT

                                        *       420         *       440         *       460         *       480         *       500
sequenced                     : ATA------------------------------------------------------------------------------------------------- :  403
LWME01000202.1:139357-138278 : ACATCTTAATATATATATATTTCTTGTGTGCGTGTGTATGTGACTGAACTCCTCCTAAACGACTGGACCGATTTAGACGAAATTTTTTGTGTGTGTTCAAGG :  499
                               A A

                                        *       520         *       540         *       560         *       580         *       600
sequenced                     : ---------------------------------------------------------------------------------------------------- :    -
LWME01000202.1:139357-138278 : GGATCTGGGATTTAGATTCACAATTTTGTCCGCTGGACAATGTTTTTTTAATTAATTTTCAATTTATTAGTTGTTGTTGATTTTGGAATGTTTTACATTG :  599

                                        *       620         *       640         *       660         *       680         *       700
sequenced                     : -----------------------------------------------------------------------------TGAATATATAAATTTACAAATGA :  426
LWME01000202.1:139357-138278 : GATCCGACAGACGGCGCTACCATGAAATTTTGAAATTAAAGACGTGTAGACAGGACAACGTCTGTCGGATCCGCTAGTGAATATATAAATTTACAAATGA :  699
                                                                                                            TGAATATATAAATTTACAAATGA

                                        *       720         *       740         *       760         *       780         *       800
sequenced                     : TAACGTTTCTGAGAACACAATTATAAGTATAGAAAAGATATTTCTAAGTAAATCACAGGCTGTAAAAGAATCACGTACCTCCCATAACCTTTAAAATACA :  526
LWME01000202.1:139357-138278 : TAACGTTTCTGAGAACAGAATTATAAGTATCGAAAAGATATTTCTAAGTAAATCACAGGCTGTAAAAGAATCACGTACCTCCCATAACCTTTAAAATACA :  799
                               TAACGTTTCTGAGAACA AATTATAAGTAT GAAAAGATATTTCTAAGTAAATCACAGGCTGTAAAAGAATCACGTACCTCCCATAACCTTTAAAATACA

                                        *       820         *       840         *       860         *       880         *       900
sequenced                     : ATCCTAATTACCCACACTAAGGGTTCTTTATGGCGATCATAATTTTATTCAGATTGAAATATAATAAAAGGACAAATTGAAGACACCATAAACGTTTTTA :  626
LWME01000202.1:139357-138278 : ATCCTAATTACCCACACGAAGGGTTCTTTATGGCGATCATAATTTTATTCAGATTGAAATATAATAAAAGGACAAATTGAAGACACCATAAACGTTTTTA :  899
                               ATCCTAATTACCCACAC AAGGGTTCTTTATGGCGATCATAATTTTATTCAGATTGAAATATAATAAAAGGACAAATTGAAGACACCATAAACGTTTTTA

                                        *       920         *       940         *       960         *       980         *      1000
sequenced                     : TAGTTGTATAAATCTTTACGAATAAAATATTGGTTTAAGTTTTTAATTTGAGATTTAATCGTTTGTTTTATAGTTTATGAAAATTGAAAAAGTTGTTTTA :  726
LWME01000202.1:139357-138278 : TAGTTGTATAAATCTTTACGAATAAAATATTGGTTTAAGTTTTTAATTTGAGATTTAATCGTTTGTTTTATAGTTTATGAAAATTGAAAAAGTTGTTTTA :  999
                               TAGTTGTATAAATCTTTACGAATAAAATATTGGTTTAAGTTTTTAATTTGAGATTTAATCGTTTGTTTTATAGTTTATGAAAATTGAAAAAGTTGTTTTA

                                        *      1020         *      1040         *      1060         *      1080
sequenced                     : TGGACGAACAAATCTTCGTCCGTTAGACAT-AAACGCAAGTTCCATAACCTCGAGCATGGCGCAGACATTACTGCATTCTT :  806
LWME01000202.1:139357-138278 : TGGACGAACAAATCTTCGTCCGTTAGACATAAAACGCAAGTTCCATAACCTCGAGCATGGCGCAGACATTACTGCATTCTT : 1080
                               TGGACGAACAAATCTTCGTCCGTTAGACAT AAACGCAAGTTCCATAACCTCGAGCATGGCGCAGACATTACTGCATTCTT
```

Figure S14

```
                                           *        20         *        40         *        60         *        80         *       100
Prap_Hel1Aa                    : --------------------TCTTAATATATATATTTCTTGTGTGCGTGTGTATGTGACTGAACTCCTCCTAAACGACTGGACCGATTTAGACGAAATTT :  80
LWME01005467.1_645_1132        : GTAAGTCCGATTTATAATCATCTTAATATATATATTTCTTGTGTGCGTGTGTATGTGACTGAACTCCTCCTAAACGACTGGACCGATTTAGACGAAATTT : 100
LWME01001026.1_73386_72899     : AAGCCGGGGCGGGTCGCTAGTCTTAATATATATATTTCTTGTGTGCGTGTGTATGTGACTGAACTCCTCCTAAACGACTGGACCGATTTAGACGAAATTT : 100
LWME01000775.1_125775_126262   : ATTCAAATTAGGTTATAATATCTTAATATATATATTTCTTGTGTGCGTGTGTATGTGACTGAACTCCTCCTAAACGACTGGACCGATTTAGACGAAATTT : 100
LWME01000397.1_220083_219596   : GGAAGTTACAATTATGAATATCTTAATATATATATTTCTTGTGTGCGTGTGTATGTGACTGAACTCCTCCTAAACGACTGGACCGATTTAGACGAAATTT : 100
LWME01000318.1_189577_189090   : GTTAAATTAAAAATAGTTTATCTTAATATATATATTTCTTGTGTGCGTGTGTATGTGACTGAACTCCTCCTAAACGACTGGACCGATTTAGACGAAATTT : 100
LWME01000220.1_194836_195323   : TTACAGCAAAATGATACATATCTTAATATATATATTTCTTGTGTGCGTGTGTATGTGACTGAACTCCTCCTAAACGACTGGACCGATTTAGACGAAATTT : 100
LWME01000197.1_94272_93785     : AGCCAAGACTGTTCTGTACATCTTAATATATATATTTCTTGTGTGCGTGTGTATGTGACTGAACTCCTCCTAAACGACTGGACCGATTTAGACGAAATTT : 100
LWME01000160.1_219201_219688   : TACATGTCCTTTTATACATATCTTAATATATATATTTCTTGTGTGCGTGTGTATGTGACTGAACTCCTCCTAAACGACTGGACCGATTTAGACGAAATTT : 100
LWME01000129.1_2104289_2103802 : CGAGATGGAATTAGTCTATATCTTAATATATATATTTCTTGTGTGCGTGTGTATGTGACTGAACTCCTCCTAAACGACTGGACCGATTTAGACGAAATTT : 100
LWME01000101.1_7839_8326       : AATTGGCGTTAAAAATTATATCTTAATATATATATTTCTTGTGTGCGTGTGTATGTGACTGAACTCCTCCTAAACGACTGGACCGATTTAGACGAAATTT : 100
LWME01000079.1_22151_22638     : ATTAAAATAGAAAACTTAAATCTTAATATATATATTTCTTGTGTGCGTGTGTATGTGACTGAACTCCTCCTAAACGACTGGACCGATTTAGACGAAATTT : 100
                                                     TCTTAATATATATATTTCTTGTGTGCGTGTGTATGTGACTGAACTCCTCCTAAACGACTGGACCGATTTAGACGAAATTT

                                           *       120         *       140         *       160         *       180         *       200
Prap_Hel1Aa                    : TTTGTGTGTGTTCAAGGGGATCTGGGAATGGTTTAGATTCACAATTTTGTCCGCTGGACAATGTTTTTTTAATTAATTTTCAATTTATTAGTTGTTGTTG : 180
LWME01005467.1_645_1132        : TTTGTGTGTGTTCAAGGGGATCTGGGAATGGTTTAGATTCACAATTTTGTCCGCTGGACAATGTTTTTTTAATTAATTTTCAATTTATTAGTTGTTGTTG : 200
LWME01001026.1_73386_72899     : TTTGTGTGTGTTCAAGGGGATCTGGGAATGGTTTAGATTCACAATTTTGTCCGCTGGACAATGTTTTTTTAATTAATTTTCAATTTATTAGTTGTTGTTG : 200
LWME01000775.1_125775_126262   : TTTGTGTGTGTTCAAGGGGATCTGGGAATGGTTTAGATTCACAATTTTGTCCGCTGGACAATGTTTTTTTAATTAATTTTCAATTTATTAGTTGTTGTTG : 200
LWME01000397.1_220083_219596   : TTTGTGTGTGTTCAAGGGGATCTGGGAATGGTTTAGATTCACAATTTTGTCCGCTGGACAATGTTTTTTTAATTAATTTTCAATTTATTAGTTGTTGTTG : 200
LWME01000318.1_189577_189090   : TTTGTGTGTGTTCAAGGGGATCTGGGAATGGTTTAGATTCACAATTTTGTCCGCTGGACAATGTTTTTTTAATTAATTTTCAATTTATTAGTTGTTGTTG : 200
LWME01000220.1_194836_195323   : TTTGTGTGTGTTCAAGGGGATCTGGGAATGGTTTAGATTCACAATTTTGTCCGCTGGACAATGTTTTTTTAATTAATTTTCAATTTATTAGTTGTTGTTG : 200
LWME01000197.1_94272_93785     : TTTGTGTGTGTTCAAGGGGATCTGGGAATGGTTTAGATTCACAATTTTGTCCGCTGGACAATGTTTTTTTAATTAATTTTCAATTTATTAGTTGTTGTTG : 200
LWME01000160.1_219201_219688   : TTTGTGTGTGTTCAAGGGGATCTGGGAATGGTTTAGATTCACAATTTTGTCCGCTGGACAATGTTTTTTTAATTAATTTTCAATTTATTAGTTGTTGTTG : 200
LWME01000129.1_2104289_2103802 : TTTGTGTGTGTTCAAGGGGATCTGGGAATGGTTTAGATTCACAATTTTGTCCGCTGGACAATGTTTTTTTAATTAATTTTCAATTTATTAGTTGTTGTTG : 200
LWME01000101.1_7839_8326       : TTTGTGTGTGTTCAAGGGGATCTGGGAATGGTTTAGATTCACAATTTTGTCCGCTGGACAATGTTTTTTTAATTAATTTTCAATTTATTAGTTGTTGTTG : 200
LWME01000079.1_22151_22638     : TTTGTGTGTGTTCAAGGGGATCTGGGAATGGTTTAGATTCACAATTTTGTCCGCTGGACAATGTTTTTTTAATTAATTTTCAATTTATTAGTTGTTGTTG : 200
                                 TTTGTGTGTGTTCAAGGGGATCTGGGAATGGTTTAGATTCACAATTTTGTCCGCTGGACAATGTTTTTTTAATTAATTTTCAATTTATTAGTTGTTGTTG

                                           *       220         *       240         *       260         *       280         *       300
Prap_Hel1Aa                    : ATTTTGGAATGTTTTACATTGGATCCGACAGACGGCGCTACCATCGCAGTGTCAAATTTTAAATAATATTCGAATTTTAATTTTAGTCTGTCCCGAAATT : 280
LWME01005467.1_645_1132        : ATTTTGGAATGTTTTACATTGGATCCGACAGACGGCGCTACCATCGCAGTGTCAAATTTTAAATAATATTCGAATTTTAATTTTAGTCTGTCCCGAAATT : 300
LWME01001026.1_73386_72899     : ATTTTGGAATGTTTTACATTGGATCCGACAGACGGCGCTACCATCGCAGTGTCAAATTTTAAATAATATTCGAATTTTAATTTTAGTCTGTCCCGAAATT : 300
LWME01000775.1_125775_126262   : ATTTTGGAATGTTTTACATTGGATCCGACAGACGGCGCTACCATCGCAGTGTCAAATTTTAAATAATATTCGAATTTTAATTTTAGTCTGTCCCGAAATT : 300
LWME01000397.1_220083_219596   : ATTTTGGAATGTTTTACATTGGATCCGACAGACGGCGCTACCATCGCAGTGTCAAATTTTAAATAATATTCGAATTTTAATTTTAGTCTGTCCCGAAATT : 300
LWME01000318.1_189577_189090   : ATTTTGGAATGTTTTACATTGGATCCGACAGACGGCGCTACCATCGCAGTGTCAAATTTTAAATAATATTCGAATTTTAATTTTAGTCTGTCCCGAAATT : 300
LWME01000220.1_194836_195323   : ATTTTGGAATGTTTTACATTGGATCCGACAGACGGCGCTACCATCGCAGTGTCAAATTTTAAATAATATTCGAATTTTAATTTTAGTCTGTCCCGAAATT : 300
LWME01000197.1_94272_93785     : ATTTTGGAATGTTTTACATTGGATCCGACAGACGGCGCTACCATCGCAGTGTCAAATTTTAAATAATATTCGAATTTTAATTTTAGTCTGTCCCGAAATT : 300
LWME01000160.1_219201_219688   : ATTTTGGAATGTTTTACATTGGATCCGACAGACGGCGCTACCATCGCAGTGTCAAATTTTAAATAATATTCGAATTTTAATTTTAGTCTGTCCCGAAATT : 300
LWME01000129.1_2104289_2103802 : ATTTTGGAATGTTTTACATTGGATCCGACAGACGGCGCTACCATCGCAGTGTCAAATTTTAAATAATATTCGAATTTTAATTTTAGTCTGTCCCGAAATT : 300
LWME01000101.1_7839_8326       : ATTTTGGAATGTTTTACATTGGATCCGACAGACGGCGCTACCATCGCAGTGTCAAATTTTAAATAATATTCGAATTTTAATTTTAGTCTGTCCCGAAATT : 300
LWME01000079.1_22151_22638     : ATTTTGGAATGTTTTACATTGGATCCGACAGACGGCGCTACCATCGCAGTGTCAAATTTTAAATAATATTCGAATTTTAATTTTAGTCTGTCCCGAAATT : 300
                                 ATTTTGGAATGTTTTACATTGGATCCGACAGACGGCGCTACCATCGCAGTGTCAAATTTTAAATAATATTCGAATTTTAATTTTAGTCTGTCCCGAAATT

                                           *       320         *       340         *       360         *       380         *       400
Prap_Hel1Aa                    : TAAAAAAAGTTTTGTTATCATTGTGTTATATCGTGTGTGACCATGTGCTGGATCGTTAGATATTGTCATAACATTTGAATAATAATTTTCATCAAAATGG : 380
LWME01005467.1_645_1132        : TAAAAAAAGTTTTGTTATCATTGTGTTATATCGTGTGTGACCATGTGCTGGATCGTTAGATATTGTCATAACATTTGAATAATAATTTTCATCAAAATGG : 400
LWME01001026.1_73386_72899     : TAAAAAAAGTTTTGTTATCATTGTGTTATATCGTGTGTGACCATGTGCTGGATCGTTAGATATTGTCATAACATTTGAATAATAATTTTCATCAAAATGG : 400
LWME01000775.1_125775_126262   : TAAAAAAAGTTTTGTTATCATTGTGTTATATCGTGTGTGACCATGTGCTGGATCGTTAGATATTGTCATAACATTTGAATAATAATTTTCATCAAAATGG : 400
LWME01000397.1_220083_219596   : TAAAAAAAGTTTTGTTATCATTGTGTTATATCGTGTGTGACCATGTGCTGGATCGTTAGATATTGTCATAACATTTGAATAATAATTTTCATCAAAATGG : 400
LWME01000318.1_189577_189090   : TAAAAAAAGTTTTGTTATCATTGTGTTATATCGTGTGTGACCATGTGCTGGATCGTTAGATATTGTCATAACATTTGAATAATAATTTTCATCAAAATGG : 400
LWME01000220.1_194836_195323   : TAAAAAAAGTTTTGTTATCATTGTGTTATATCGTGTGTGACCATGTGCTGGATCGTTAGATATTGTCATAACATTTGAATAATAATTTTCATCAAAATGG : 400
LWME01000197.1_94272_93785     : TAAAAAAAGTTTTGTTATCATTGTGTTATATCGTGTGTGACCATGTGCTGGATCGTTAGATATTGTCATAACATTTGAATAATAATTTTCATCAAAATGG : 400
LWME01000160.1_219201_219688   : TAAAAAAAGTTTTGTTATCATTGTGTTATATCGTGTGTGACCATGTGCTGGATCGTTAGATATTGTCATAACATTTGAATAATAATTTTCATCAAAATGG : 400
LWME01000129.1_2104289_2103802 : TAAAAAAAGTTTTGTTATCATTGTGTTATATCGTGTGTGACCATGTGCTGGATCGTTAGATATTGTCATAACATTTGAATAATAATTTTCATCAAAATGG : 400
LWME01000101.1_7839_8326       : TAAAAAAAGTTTTGTTATCATTGTGTTATATCGTGTGTGACCATGTGCTGGATCGTTAGATATTGTCATAACATTTGAATAATAATTTTCATCAAAATGG : 400
LWME01000079.1_22151_22638     : TAAAAAAAGTTTTGTTATCATTGTGTTATATCGTGTGTGACCATGTGCTGGATCGTTAGATATTGTCATAACATTTGAATAATAATTTTCATCAAAATGG : 400
                                 TAAAAAAAGTTTTGTTATCATTGTGTTATATCGTGTGTGACCATGTGCTGGATCGTTAGATATTGTCATAACATTTGAATAATAATTTTCATCAAAATGG

                                           *       420         *       440         *       460         *       480
Prap_Hel1Aa                    : CTTATTAAAAATTGAAATTTTGAAATTAAAGACGTGTAGACAGGACAACGTCTGTCGGATCCGCTAG-------------------- : 447
LWME01005467.1_645_1132        : CTTATTAAAAATTGAAATTTTGAAATTAAAGACGTGTAGACAGGACAACGTCTGTCGGATCCGCTAGTACTACATATAAATATCAAT : 487
LWME01001026.1_73386_72899     : CTTATTAAAAATTGAAATTTTGAAATTAAAGACGTGTAGACAGGACAACGTCTGTCGGATCCGCTAGTATATTATAAATATTGAGAG : 487
LWME01000775.1_125775_126262   : CTTATTAAAAATTGAAATTTTGAAATTAAAGACGTGTAGACAGGACAACGTCTGTCGGATCCGCTAGTTATAATATAAATATATATA : 487
LWME01000397.1_220083_219596   : CTTATTAAAAATTGAAATTTTGAAATTAAAGACGTGTAGACAGGACAACGTCTGTCGGATCCGCTAGTAAGTATATATAAAGGGAGG : 487
LWME01000318.1_189577_189090   : CTTATTAAAAATTGAAATTTTGAAATTAAAGACGTGTAGACAGGACAACGTCTGTCGGATCCGCTAGTACGATATAAATGTTTGTTT : 487
LWME01000220.1_194836_195323   : CTTATTAAAAATTGAAATTTTGAAATTAAAGACGTGTAGACAGGACAACGTCTGTCGGATCCGCTAGTAACGGTGTAAATTGAAAAC : 487
LWME01000197.1_94272_93785     : CTTATTAAAAATTGAAATTTTGAAATTAAAGACGTGTAGACAGGACAACGTCTGTCGGATCCGCTAGTGTTTATGTATTTTGCTCAC : 487
LWME01000160.1_219201_219688   : CTTATTAAAAATTGAAATTTTGAAATTAAAGACGTGTAGACAGGACAACGTCTGTCGGATCCGCTAGTTATTATTTATATATTTCCT : 487
LWME01000129.1_2104289_2103802 : CTTATTAAAAATTGAAATTTTGAAATTAAAGACGTGTAGACAGGACAACGTCTGTCGGATCCGCTAGTATATATACAAATATTTTAG : 487
LWME01000101.1_7839_8326       : CTTATTAAAAATTGAAATTTTGAAATTAAAGACGTGTAGACAGGACAACGTCTGTCGGATCCGCTAGTTTTATTATATATAAGAGAA : 487
LWME01000079.1_22151_22638     : CTTATTAAAAATTGAAATTTTGAAATTAAAGACGTGTAGACAGGACAACGTCTGTCGGATCCGCTAGTTATTTATAAATATTAAGTC : 487
                                 CTTATTAAAAATTGAAATTTTGAAATTAAAGACGTGTAGACAGGACAACGTCTGTCGGATCCGCTAG
```

Figure S15

**a**

```
                         *        20         *        40         *        60         *        80
Bmor_Hel1Ca : TCTTTATATTATATATTTCTTCTGTGCGTGTGTTTGTCACTGAACTCCTCCTAAACGGCTGGACCGATTT-AATG--ATTT :  78
Cvic_Hel1Ca : TCTTTATA-TATATAATTCTTCTGTACGTGTGTTAGTAACTGAACTCCTCCTTAACGGCTGGGCCGATTTCGATGAAATTT :  80
Emus_Hel1Ca : TCTTTATA-TATATAATTCTTCTGAAAGTGTGTTAGTAACTGAACTCCTCCTTAACGGCTGGGCCGATTTCGATGAAATTT :  80
              TCTTTATA TATATAaTTCTTCTGtacGTGTGTTaGTaACTGAACTCCTCCTtAACGGCTGGgCCGATTTcgATGaaATTT

                        *       100         *       120         *       140         *       160
Bmor_Hel1Ca : TTTGTATCTTT----GGTGGCCCCTGGATGGTTTAGATTCACAAATCAGCCCGGCAGA-----------TGGCGCTGC--- : 141
Cvic_Hel1Ca : GTTGTGTGTGTTTGAGTGGGTCCCTGGATGGTTTAGATTCACAATTGACCTATATAGG-ACAATGGGCATGGCACCTCCCA : 160
Emus_Hel1Ca : TTTGTGTGTATTAGAGTTGGTCCCTGGATAGTTTAGATTTACAATTGACCTATATAGGTACAATGGGCGTGGCACCTCCCA : 161
              tTTGTgTgT Tt gaGttGGtCCCTGGATgGTTTAGATTcACAAtTgAcCtatatAGg acaatgggc TGGCaCctCcca

                       *       180         *       200         *       220         *       240
Bmor_Hel1Ca : -----AGTCGGTATCTAGGTTATTTTATCTTT---------------------CCTAGAAATAATTTATAT---------- : 186
Cvic_Hel1Ca : TACAAAGTAAAAATTTA--TTATTGCATATCTGGAGTACTATTATAGTGGGAGTCTTCAAAC--TTTGTGTGAGCTAT--- : 234
Emus_Hel1Ca : TACGAAGTAAAAATTTA--TWATTGCATATCTGAAGCACTATTACAGCGGGAGTCTTCAAAC--TTTGTGTGAGCTGTTTT : 238
              tac aAGTaaaaATtTA  TtATTgcATaTcTg ag actatta ag gggagtCTtcAAAc  TTTgTgTgagct t

                      *       260         *       280         *       300         *       320
Bmor_Hel1Ca : --------------------------------------------------GGCAAAACAACGTTTGCCGGGCCAGCTAG : 215
Cvic_Hel1Ca : --------------------------------------------------GGCAGAACAACGTTTGCCGGGACAGCTAG : 263
Emus_Hel1Ca : GATAATAATATGCTATTTGTTGAAAAGTCATTAGCCTTAAATACAATTTAGGCAGGACAACGTCTGCCGGGTCAGCTAG : 318
                                                                GGCAgaACAACGTtTGCCGGG CAGCTAG
```

**b**

```
                           *        20         *        40         *        60         *        80         *       100
Cvic_Hel1Ca.1 : -CTTTATATATATAATTCTTCTGTACGTGTGTTAGTAACTGAACTCCTCCTTAACGGCTGGACCGATTTCGATGAAATTGTTTGTGTGTGTTTGAGTGGG :  99
Cvic_Hel1Ca.2 : -CTTTATATATATAATTCTTCTGTACGTGTGTTAGTAACTGAACTCCTCCTTAACGGCTGGGCCGATTTCGATGAAATTTGTTGTGTATGTTTGAGTGGG :  99
Emus_Hel1Ca   : TCTTTATATATATAATTCTTCTGAAAGTGTGTTAGTAACTGAACTCCTCCTTAACGGCTGGGCCGATTTCGATGAAATTTTTTGTGTGTATTAGAGTTGG : 100
Bmor_Hel1Ca.1 : TCTTGATATATATATTTCTTCTGTGCGTGTGTTTGTCACTGAACTCCTCCTAAACGGCTGGACCGATTTCAATGAAATTTTCTGTGTATCTTCGAGTGGA : 100
                 CTTtATATATATAaTTCTTCTGtacGTGTGTTaGTaACTGAACTCCTCCTtAACGGCTGG CCGATTTCgATGAAATTtttTGTGT T TT GAGTgGg

                           *       120         *       140         *       160         *       180         *       200
Cvic_Hel1Ca.1 : TCCCTGGATGGTTTAGATTCACAATTGACATATATAGGTACAATGGGCATGGCAATTCCCATACAAAGTAAAAATTTATTATTGCATATCTGGAGTACTA : 199
Cvic_Hel1Ca.2 : TCCCTGGATAGTTTAGATTCACAATTGACCTATATAGGTACAATGGGGATGGTAACTCCCATACAAAGTAAAAATTTATTATTGCATATCTGGAGTACTA : 199
Emus_Hel1Ca   : TCCCTGGATAGTTTAGATTTACAATTGACCTATATAGGTACAATGGGCGTGGCACCTCCCATACGAAGTAAAAATTTATWATTGCATATCTGAAGCACTA : 200
Bmor_Hel1Ca.1 : TTCGAGAATGGTTTAGATTTACAAATCAGCCCGGCAGAT------GGCGTTGCAGTCGGCGTCTAGGTT----ATTTATCTTTCC--------------- : 175
                TcCctGgAT GTTTAGATT ACAAtTgAcctatatAGgTacaatgGGc TgGcA  tccCaTacaaagTaaaaATTTAT aTTgCatatctg ag acta

                           *       220         *       240         *       260         *       280         *       300
Cvic_Hel1Ca.1 : TTATAGTGGGAGTCTTCAAACTTTGTGTGGGCTAT------------------------------------------------------AACAGAACAAC : 245
Cvic_Hel1Ca.2 : TTATAGTGGGAGTCTTCAAACTTTGTGTGAGCTAT------------------------------------------------------GGCAGAATAAC : 245
Emus_Hel1Ca   : TTACAGCGGGAGTCTTCAAACTTTGTGTGAGCTGTTTTGATAATAATATGCTATTTGTTGAAAAGTCATTAGCCTTAAATACAATTTAGGCAGGACAAC : 300
Bmor_Hel1Ca.1 : ------CAGAAAT------AATTTATAT-------------------------------------------------------------GACAAAACAAC : 202
                tta ag gGgAgTcttcaaAcTTTgTgTg gct t                                                    g CAgaAcAAC

                           *
Cvic_Hel1Ca.1 : GTTTGCCGGGATAGCAAG : 263
Cvic_Hel1Ca.2 : GTTTGCCGGGACAGCTAG : 263
Emus_Hel1Ca   : GTCTGCCGGGTCAGCTAG : 318
Bmor_Hel1Ca.1 : GTTTGCCGGGCCAGATA- : 219
                GTtTGCCGGG cAGctAg
```

Figure S16

**a**

```
                       *        20         *        40         *        60         *        80         *       100
Pgla_Hel1Ga : TCTT--TATATATATATTTCTTGTGTGCGTGTGTATGTCACTGAACTCCTCCTAGACGGCTGGACCGATTTTGATGAAATTTTTTGTGTGAGTTCAAGGG :  98
Hvit_Hel1Ga : TCTTTATATATATATATTTCTTGTGTTCGTGTGTATGTGACTGAACTCCTCCTAAACGACTGGACCGATTTTGATGAAATTTTGTGTGTGTTTTCAATGG : 100
              TCTT  TATATATATATTTCTTGTGT CGTGTGTATGT ACTGAACTCCTCCTA ACG CTGGACCGATTTTGATGAAATTTT TGTGTG  TTCAA GG

                       *       120         *       140         *       160         *       180         *       200
Pgla_Hel1Ga : GATTCGAGGATGGTTTAGATTCACAATAT----------------------------------------------------------------------- : 127
Hvit_Hel1Ga : AATTCGAGAATGGTTTAGATTCACAATTTGGTCCAATTAAATTGTTTTTAATTAATTTTTATTTATAAATTGTTGTTGATTTTGGAGTGTTTTACTTGA : 200
               ATTCGAG ATGGTTTAGATTCACAAT T

                       *       220         *       240         *       260         *       280         *       300
Pgla_Hel1Ga : ---------------------------------------------------------------------------------------------------- :   -
Hvit_Hel1Ga : TCCGGCAGACTGCGCTACGACACGTAATACAAAGTGAACTGATACTTAACAGCTGTTAATACTTTCAGCTGAAGTTCATCAAAGAGGCAGAAACATTTGT : 300

                       *       320         *       340         *       360         *       380         *       400
Pgla_Hel1Ga : ---------------------------------------------------------------------------------------------------- :   -
Hvit_Hel1Ga : TTACATTTAAAATTTAGAAAATTATTATTGTAAAGTGCTTGATCAGTAGTGTAATGCAGATTGCATAGAAGAAGTTATTGCCTAATTTTAAATTTAGATT : 400

                       *       420         *       440         *       460         *       480         *       500
Pgla_Hel1Ga : ---------------------------------------------------------------------------------------------ATATAAG : 134
Hvit_Hel1Ga : GCACCAATGATCAATAAACTATCTAATGCAATGAAAATACTATTAAACGCTGTTATAAAAACCACCTCATTGTACAAAGCCGTGAATGTTTGCACATAAG : 500
                                                                                                            A ATAAG

                       *       520         *       540         *       560         *       580         *       600
Pgla_Hel1Ga : G--------------------------------------------------------------------------------------------------- : 135
Hvit_Hel1Ga : GTAATCGAATTTGGTTAGATCGAAGGTAAATGAAAAGAGCCGAAAGAAGACGCTTTATGATCGAAATTGGTTTTCGTTGATACATTTTCTTGATCGGAGC : 600
              G

                       *       620         *       640         *       660         *       680         *       700
Pgla_Hel1Ga : ---------------------------------------------------------------------------------------------------- :   -
Hvit_Hel1Ga : ACAAGGCAAACTCCACAATAATACTGGAAATATCTTAGACAGAAAATTAATTTTAACAGACCGAATTTGATTCTTTATAACCTAATTAGTTTATCGAGAT : 700

                       *       720         *       740         *       760         *       780         *       800
Pgla_Hel1Ga : ---------------------------------------------------------------------------------------------------- :   -
Hvit_Hel1Ga : TCATCCATATAAATTAATTGTACTGAATAACTTATCAACACCTAGCAAAAACCACGAAAGATAGAGGCAGGAAATATGGTACATACCTTCATAATGCGCC : 800

                       *       820         *       840         *       860         *       880         *       900
Pgla_Hel1Ga : ---------------------------------------------------------------------------------------------------- :   -
Hvit_Hel1Ga : CAAGAGGCTCACGAAGGAACGACTATCAATTATCTCAGGAATGTTTAGAATGTGATAGAAAAAGAATACGTGAATATAGTGTACTGTTTTTCAACAGGAA : 900

                       *       920         *
Pgla_Hel1Ga : AGTGCGTGCGAAGCCGCGGGCAACTGCTAG : 165
Hvit_Hel1Ga : ATTGCGTGCGAAGCCGCGGGCAACTGCTAG : 930
              A TGCGTGCGAAGCCGCGGGCAACTGCTAG
```

**b**

| | Pgla_Hel1Ga | Hvit_Hel1Ga |
|---|---|---|
| Pgla_Hel1Ga | | |
| Hvit_Hel1Ga | 0.079 | |

Figure S17

```
                       *        20         *        40         *        60         *        80
Csup_Hel1Aa : TCTTTATATATATAATTCTTCTGTGAGTGTGTATGTCACTGAACTTCTCTTAAACGACTGGACCGATTTTGATGAAATTT :  80
Csup_Hel1Ab : TCTTTATATATATAATTCTTCTGTGAGTGTGTATGTCACTGAACTTCTCTTAAACGACTGGACCGATTTTGATGAAATTT :  80
              TCTTTATATATATAATTCTTCTGTGAGTGTGTATGTCACTGAACTTCTCTTAAACGACTGGACCGATTTTGATGAAATTT

                       *       100         *       120         *       140         *       160
Csup_Hel1Aa : TTTGTGTGTGTTCAAGGGGATCTGAGAATGGTTTAGATTCACAA------------------------------------ : 124
Csup_Hel1Ab : TTTGTGTGTGTTCAAGGGGATCTGAGAATGGTTTAGATTCACAATTTTGTCCGCTGGACAATGTTTTTTTAATTAATTTT : 160
              TTTGTGTGTGTTCAAGGGGATCTGAGAATGGTTTAGATTCACAA

                       *       180         *       200         *       220         *       240
Csup_Hel1Aa : -------------------------------------------------AGACGT----------GTAGACAGGACAACG : 145
Csup_Hel1Ab : TAATTTATTAGTAGTTGTTGATTTTGGAATGTTTTACATTGGATCCGACAGACGGCGCTACCATACTAGACAGGACAACG : 240
                                                               AGACG            TAGACAGGACAACG

                       *
Csup_Hel1Aa : TCTGTCGGGTCCGCTAG : 162
Csup_Hel1Ab : TCTGTCGGGTCCGCTAG : 257
              TCTGTCGGGTCCGCTAG
```

Figure S18

```
                        *        20         *        40         *        60
Pxut_Hel1Aa : TCTTTATATATATAATTCTTCTGTGAGTGTGTATGTCACTGAACTTCTCTCAAACGACTGGACCG :  65
Pxut_Hel1Ab : TCTTTATATATATAATTCTTCTGTGAGTGTGTATGTCACTGAACTTCTCTCAAACGACTGGACCG :  65
              TCTTTATATATATAATTCTTCTGTGAGTGTGTATGTCACTGAACTTCTCTCAAACGACTGGACCG

                   *        80         *       100         *       120         *
Pxut_Hel1Aa : ATTTTGATGAAATTTTTTGTGTGTGTTCAAGGGGATCTGGGAATGGTTTAGATTCACAATTTTGT : 130
Pxut_Hel1Ab : ATTTTGATGAAATTTTTTGTGTGTGTTCAAGGGGATCTGGGAATGGTTTAGATTCACAATTTT-- : 128
              ATTTTGATGAAATTTTTTGTGTGTGTTCAAGGGGATCTGGGAATGGTTTAGATTCACAATTTT

                     140         *       160         *       180         *
Pxut_Hel1Aa : CCGCTGGACAATGTTTTTTTAATTAATTTTTAATTTATTAGTAGTTGTTGATTTTGGAATGTTTT : 195
Pxut_Hel1Ab : --------------------CATCAAATATTAACTTCATATTAATT------------------- : 154
                                   AT AA T TTAA TT  TA TA TT

                   200         *       220         *       240         *      260
Pxut_Hel1Aa : ACATTGGATCCGACAGACGGCGCTACCATCGCAGTGTCAAATTTTAAATAATATTCAAATTAAAG : 260
Pxut_Hel1Ab : ---------------------------------------------------TATTCAAATTAAAG : 168
                                                                   TATTCAAATTAAAG

                        *       280         *
Pxut_Hel1Aa : ACGTGTAGACAGGACAACGTCTGTCGGGTCCGCTAG : 296
Pxut_Hel1Ab : ACGTGTAGACAGGACAACGTCTGTCGGGTCCGCTAG : 204
              ACGTGTAGACAGGACAACGTCTGTCGGGTCCGCTAG
```

Figure S19
