# Supplementary material for: Characterization of a novel Helitron family in insect genomes: insights into classification, evolution and horizontal transfer
Source: Mob DNA. 2019 May 31;10:25. doi: 10.1186/s13100-019-0165-4 (PMC6544945; doi:10.1186/s13100-019-0165-4)
Supplement: Supplementary file 3 — Data S1. The Hel1 elements with degenerated remnants of Helitron coding sequences identified in insect and spider genome databases. Data S2. The reconstructed potential autonomous Hel1 Helitrons. (PDF 67 kb) [file 13100_2019_165_MOESM3_ESM.pdf]

Data S1. The *Hel1* elements with degenerated remnants of *Helitron* coding sequences identified in insect and spider genome databases.

>*Nephila clavipes* (MWRG01000558.1: 165496-162173)

tcttcatatatataattcttctgtgcgtgtgtatgcgactgaactcctaaatggctgggccgattttta
tgacattttgtgtgtgtgttctagtgaattcgaaagtggtttatagattcacaatttggtccgatatcg
caattagttaaaatatattaattaaaaacttaaaaatgcatatttgctttacccctgtgtactatttag
tctgctaatgcgcggaaagaaaagaaagacatattttatatataattcttctgtgcgtgtgtatgtga
ctgaactcctcctaaacggctggacagattttgatgacatttttgtgtgggttccagtggattcgaga
atggtttagattcacaatttggtccaatggaaaatgtttacttaattaatttttttatttataactttt
ttttatatcttggaatgttttacattggattcggcagactgcgttacgatcgcagatcgtattcaaaaa
tttttattataattccagtttgtcccgacagttggtgctgcaatcaaattcaggaaaatattttgttac
ttcgtgtagacattgataatcgctggatggttccatattcgccattgctgtgcaaaatctacagagaac
acataaacgttgagttatgcagttaggagaagtccatcaagtacatttgtacgtacgttcataaaggga
gtgataaagctatatttgccgttaaaaatgtaaacgacaatgatgaaataacacgttatcacaaaaaag
ccgataaatttgtagtaacgaagcaaattggcgcatttttagttttcctatacatgaaagggatactgc
tgttatacatttggcaatgcactttgaaaatggagcacgtttatttcacagagcagattgctctacaac
aagctttatcagctccaaaaacaacccttaccgaatttttcaacctttgtaatcgacaagatgctattg
gtcaactcgcaaggacattaatgtatactgatgttcctaaatttttttacaaggattaagcaatcaaaaa
ggagccacgaaaacgaggctttcaagtcccaggattcgccggtatatttatggcagatactttaggtcg
attatatacagttaatcctaagaaacgcgaatgtttttttcagcgtttgcttttgtttaacgttcctgg
accaacatctttacaagatttacgaaaagccgatgcgtgtcgtgagttacatttattggaagatgataa
ccattgggatctcacatttgcagaggcagtactgagttaatctccacagcaaactcgtcaattattttc
aataacattaacaaagtgttttccgtctcaggcatctgctatgtgggaacaaatacaaaaacttaataa
atgagaatattttgcatcggattagaattactgatcaaaatctcaatggagaattttggccgaaatat
ataacgaggcattaataatgattgaggatatttgtatttttatttcaaacatggcactcatctatttg
gcatgccagtaccgaaccacccatcagtggatatcatcaacagcgatgttcaacgtgaacaccagtttg
ataggacgtttttggctactttttttgctgatgatgagcaattgcttactacttttcaacgaaatgtat
acattcaaatcaaagtatctactgcagcacgacaaggtggattctttttctgaatgcaccaggtggca
ctggtaaaacatttcttaccccactgatacttgcacgcatttgatcacaaaatcacattgcattggcaa
ttgcttcatcgggcattgcagcaactttacttgatggtggacgcactgcgcattcagcacttaaattac
ctttaatgtccatacaaatccctaagcaatgtgtaatataaagaagctttcaggcatggctgaagtttt
gaaaaaatgcaaaattattatctgggatgaatgtacaatggcccacaagcattcgcttgaagctctcga
caggcccatgaaagatataaaaaataataccaggtttttcggtggtgctctactgtcgatgtctggtga
tttcagacgaacattcccgtcattccccgtgcaaaatatgcggataaaataaacgcattcttgaaagaa
tcttatctctagcgaagtgatagtaaattatgccttaccattaatgtacgtgttcaacttcaaaaatga
ttcattagcgtcgaggttctctgaacaattgttagacattggcaatggtagaattccattgtgtatgaa
gttacaaaatatatttaacttccagaaagtttttgcaacattgtggctaccaaagatgaattaataaaa
agtatctttccagatttacgacataattatattgatcatacatggctaagtgagcgagctattttagca
gcaaaaaatttagatgttgatgtcatcaatttcaaaatacatcagtcattgcctgttaattaaattaca
ttcaaatcgattgacactgttgatgatcccgatgaagtaagtagccaactatcctgcagagtttttaaa
ttcactggatttaccgggaatgccaccacgtaatttgcgattgaaaattggctcacctataatttttat
tcgaaatttgaacacaccgaaattgtgtaacagcacgagattattcataaaaaaaatcattggcaacat
tcttgaagccactattttaaggggaaaatttcaaggttgaagttgtacttctaccacggatcccaatga
ttccttcaatttcacctataacattcaaacgcttacaatttccaattcgcttggcattttctatgacta

ttaataagtcacaaagtcaaacagtgacaaattgtggcttaactttagaaaatcctgttcttctcacgg
caaattatgtgttgagtgttccagagttgtaaaaccatcgaatatatttgtttatactcctcaaggatt
aaccaaaaatgttgtgtatacaatggcattacgataaattaagtttttgtacaatttaataaatatacc
tgttataaaatagtttgagttttaaattttaacatttaattaaaacaagtttaagtttttattcatata
acctctgaatttaacttcgctcaaagtatacacatactcacacgctatcgattgatcgaatcgtgataa
ccgtatatgtaatgacgttagatatattgtaataactatttagtaaatttttttatcaaaacggtccag
attgttaaagcttattcataaaaatttggaatttttgaatttaagatgtatgtataggacaacttccgt
cgggtccgatag

>*Parasteatoda tepidariorum* (AOMJ01259424.1: 19170-22002)

tctttatatatatatttcctctgtgcgtgtgtttgtcactgaactcctcctaagcggctggaccgcttt
aaataaaatttctgtgtaccttcagaaggattcgagaatggtttaaattcacaattaggtccaatatc
aatttttttagttaatttttattgagtgggaaaaattctacttcttaaaagttatacttcttatgcga
cttccatcaagaaagcgttaaacgtttaaagctacattttcattggctgggaaatcacgtggtaggatc
cagttttccctcattcatttccatattgttttggttctcactagcataaaaataataaaagtcataaaa
cttttgtttttctgtaaatatttttttagtttgttttgatacacatataatttaatagggagcaggaaa
aagagtcgttgaatagaatttgtaatctcgtgttattattgtgtttgaaaagttttgaagtgtgcaaat
ggacagcgtgtttatttcaccgaacagaatgcactacaacaagctttaacagctccaaaaacaactctt
actgaatttttcaacctttgtaatcgacaagatattgttggtgaatttacaaaggcattaatgtatact
gatgttcctaaatttttttacatggaataaacaatcaaaaagttgggaaccacggaaacgaggcactcca
gtcccaggatttgccgacatagttatgacaaatactttaggacgattatatacagttcttcctaagcaa
cgcgaatgctttttttctgcgtttgttattggttaacgaaaagtcaacggtgctttatacggcactttat
ttgatgcgtgtcgtgagttacatttattggaggatgataaccattgggacctcacacttgcagacgcag
cactgagttcatctccacagcaaattcgtcaattattttcaataatattgacaacgtgttttccgtctg
aagcatctgctctgtggaataaatataaagactcaatgagtgaggatatttgtatcggattagaatta
ctaatcaaaatctctgtcttgaattctccgcagaaatatataacgaggcattaataatgattgaggata
tttgttttcttatttcgaacatgccgctcatgaattttggtatgccagcgccaagcagcagatatcatc
aacagcgacgtgaacaccagttcgatatgacttatctggctacttttgttgctaacaatgaacgattgc
ttactgctgagcaacgaaatgatcaaattaacgtgtcaattgcagcacaacaaggtggatttttttctc
tggacgcaccaggtggcactggtaaaacattccttatctcactaatacttgcgctcattcgatctcaaa
atcatattgcattggcaattacttcatcaggcattgcagcaaccttactttatggaggaagaacagcac
atccagcacttaaattacctttgaatattcataccaataccgaatcaatgtgtaacataaagaagcatt
caggcatggctgaagttttgagaaaatgtaaaattattatctgggatgaatttacaatggtccacaagc
attcgcttgaagctctcgacaggtccctaaaagatatcaaagataatactcggctttttggtagtgctc
tactgctgcttgtcagatcatgtttgaaagagtcttatctgtggcgaagtgtcagtaaattatgcctta
ctcttaatatgcgtgttcaacttcaaaatgatccattagcgtcaagattctctgaacaactgttagaca
ttggcaatggtaaaattccattctatgaagatacacaatatattcgactcccacagaattttgcaaca
tggtgcctaccaaagaggagttaataaaaagtatctttccagatttgccacataattatactaatcatg
catggctacgtaaacgagctattttggccgcaaaaaatttagatgttgacgcaatcaattttaaagtac
aacaatcattgcctgggtagtgaatcctgatgaagttgtcaattatcctgccgagtttttaaattcact
ggatttacctggaatgccaccccataatttgcgattgaagattgattcacctataattttgcttagaaa
tttgaatgctccaaaattgtgtaatggcacgcgactggtcataaaaaaaatcatgggtaacattttgga
agcaactattttaagtggaaaatttaaaggtgaagttgtacttataccgcggatcccaatgattccttc
agattcgcctataccattcaaacgtttgcaatttccaatcctcttagcatttgatatgactattaataa
gtcacaaggtcaaacaatgacaatttgtggcttagatttagaaaacccgtgcctttctcacggtcaatc

atatattgcgtgttccagagttggaaaaccctcgaatttgtttgcgtatgctcctgaaggattaaccaa
aaatattgtacatctaatggcattacgataacttaagttttttttaattgaaaaaatatatctgttata
aaatagtttaagttttcacttttttacctttaattaaaataagcttaagttttgatttaaaacaacctc
tacatttaacttcgctgatagtatacacatactcacacgctatcgattggtcgaattgtgataagatat
atgtaatgacgttagataatataataacttttttaataaaaatttgcataaaaacgatccagattgttac
agcttattcaaaaaaatttgaaattttccaattaaagacgtgtgtacaggacatcgtctgtcgggtccg
ctag

>*Papilio machaon* (LADJ01066876.1: 42602-36179)

tttgaaaaaaatttttgttattcaattcatgtgctgattaaaatattaaatgtaattattttttaaagag
ttttgaagtgtgtataaataattttgtgaattcagatgtttttttaattaatttttatttgtaaggagt
ttttgattttggaatgttttacatcagattcgacggatggcgctatcatcgcattcaatattattcaat
ttcaattttagtttgtcccgacagatggtgctacgattaatttgaaaaaaatttttgttattcaattcat
gtgctgattaaaatattaaatgtaattatttttttaaagagttttgaagtgtgtataaataattttgtga
attcagatgtttttttaattgatttttatttgtaaggagtttttgattttggaatgttttacatcagat
tcgatggatggcgctatcatcgcattcaatattattctattttaattttagtttgtcccgacagatagt
gctacgatcaatttgaaaaagtatttcgttattttgtgttataattgtgttattcaagtgaagttgcta
attaaaatatttaatgtaattagttttcgaagagttctgaagtgtatataaataacttatacattttgt
aaatttagtgcggaatcgaagtgagtataaccttcaagtttatttgtgtagttagaggattgaatttt
ttaattatttttattgacaattaattcatttttggagatattaaataaaaaaactcacaaaatgcgatt
acatgttctttatctaaaatttgtcataataaatcttttgagttaatactcataattatatttatttac
aggtgaagaactgaccaagaaagttagtgccatgaatttctacgcatatcggttaatgattcatgctaa
tgaagataataatattctccgctgtagacagctatttcatcagtgtaattaaaaaaaaataatttagaa
ataaaatctgtcataaaaaattcattatcaataagtaactcatattcattgtatattattttgcaatat
tttatttttacagtagaagaacattataactcatattcattgtatattattttgcaatattttattttt
gtagaaaaatgccacgtcttcgtggaagagcaagaaatattggtcgacgtactcagcacgcgcaattag
ttcatgatcatcgattgaatagaacagtagaagaacattctatggataatgtaaatttaagagatcgag
ctgcatgtacacgtgcaaatgaaaatttagagcaacgtgctcaacgtcttcgtgctaatacattaagac
aacgagaggcacgtcaacgagcgaccaacgcacatagagaacgtaaccaacagcgagtacaagataatc
gagcattggcacgagcatcacttaatcgtctcgcgtttgaatatgatcctgaaatagattattcatcac
atgcattgatcacgattggtagtatggacaaagagtgtcaacattgtcatgctttcaagtacaaaggtg
aatcagctggtttatgttgcgcatctggaaaaatatcactgccaccgctaaaaccaccaccagaacctt
taaaaacgcttttagctggaatcacatctcaatcgaaattatttttacggaaaattcgtaaattaaatt
catgcttccaaatgacatcatttgcagcaacaaaaattattcataatgaagatggccgtaattttgaat
ccacattcaaaattcaaggccaagtatatcaccaaattggttcgttacttccaatgcctgatgccgatc
caaaatttttacaaatttattttatggggaatgaagagcaacaatcacatacacgctgcgtttacaacc
atatagaacagatggaggaacgagaaattgtggacattttggaaaggtttttgcaaaaccataaccaat
tagtacaattgttcaagactctttctaacagactgcaaaacgataactatgtcattgttattaaagcag
acaaagtaccctatggagagcacgcaggcacatataatgttccaaccattaatgaagtggcagttgtta
tggctggtgacccatgtgaacgtagagacattcgcatacaacgcagagataatacgatgcaaataattc
aagacaatcatcgttcttacgatgctttgcagtatccgttaatattttgggaaggagaagacggatatc
atttaaatattaaacaacggaatccaactacaggtacaacttatagattatcttacaattttttttttg
aataaataatttttgtttatctcataattatatttatttacaggtgaagaactgaccaagaaagttagt
gccatgaatttctacgcatatcggttaatgattcgtgctaatgaagataataatattctccgctgtaga
cagctatttcatcagtacatcgtcgatatgtatgtaaaaattgaaagtgaaagattaaggtatataaaa

ttcaatcaagcaaaacttcgagctgaagagtatattcatttgcgtgatgcagtaatcggtaatgtagat
gcaacaaacgtcattaacaacatcggtaccacatatattctcccatcatcatatattggtagtccgcgt
catatgcaagaatatattcaagacgctatgacatacgtacgtgcatacggtcgaccagatctttttatc
acatttacatgtaatccaaattgggatgaaatcaaaaacttgctgttgtcaggtcaaacatcgatgcat
cgccacgatatcactgcgcgtgtctttaaacaaaaattaaaatctttgatgaatttgatcacacattac
tcggtttttggtgaaacacgttgttggctttactctgttgagtggcaaaaacgaggtttacctcatgcg
catattttgatttggttggtggataaagtacgcccagaagaaattgacaaaattatttcagcggaaatt
ccagatccgaatgttgatcaagaactgtttaacattgttactactaatatgattcatggtccatgcggt
actctaaacatgatgtcaccatgcatgaatgatggaaaatgtacgaaacgttttccaaaaccatgtcaa
actgatactatcaccaatattgacggttatccatcttaccggcgcagagacgtagataatggcggtcaa
tcgtatgaattgcgactgtcaaacggtgtaagagtagatattgataatcggtgggtggttccatattcg
ccattactatgcaaaacctataaagcgcacataaacgttgaactgtgcagttcggtgaaatctatcaaa
tacatttgtaagtacgttcacaagggcagtgataaagctatatttgctgttcaaaatgtaaatgacaat
gacgaaataacacgttatcaaatgggtcgatacataagtagcaatgaagctgtctggcgcattttttacg
tttcctatacatgaaagggatcctgcagttatacatttggctgtgcatctttgaaaacggacagcgtgt
ttattttacagaacagactgcactacaacaagcattaacggctccaaaaacaactcttactgaattttt
caatctttgtaatcgacaagatgttgttggtcaattcgcaaaaacattaatgtatactgatgttcctaa
atttttttacatggaataagcaatcgaaaaattgggaaccacgaaaacgagggattccagtcacaggatt
cgccgacatatttatgacaaatactttaggtcgactatatacagttcatcctaagcaacgcgaatgttt
ttttttgcgtttgttattggttaatgttcttggaccgacatccttccaatatttacgaaaagtcaatgg
tattttatacgacactttcttcgatgcgtgtcgtgagttacatttattggaggatgataaccattggga
tctcacacttgcagatgcagcactgagctcatctccacaacaaattcgtcaattattttcaataatatt
aacaacgtgttttccgtctgaagcatctgctctctggaacaaatataaagactcaatgagtgaagatat
tttgcatcggattagaattactaatcaaaatcctgatattcaattctccgcagaaatatacaatgaggc
tttaattatgattgaggatatttgtattcttatttcaaacatgccactcatccattttggcatgccggc
gccgaaccgcccagcagtagatatcatcaacagtgatgttcaacgtgaacaccagtttgataagacttc
gttggctacttttgttgctaataatgaacaattgcttacagctgaacaacgaaatgtatatgatcaaat
taacgtatcaattgcagcacaacaaggtggattctttttttggatgcaccaggtggcactggtaaaac
ctttctcatctcactgatttaagtgtttctatggcaatccacttccactgttcaagaaaacatactctg
cgaacaatttcaaacatgccactcatccattttggcatgccggcgccgaaccgcccagcagtagatatc
atcaacagtgatgttgaacgtgaacaccagtttgataagacttcgttggctacttttgttgctaataat
gaacaattgcttacagctgaacaacgaaatgtatatgatcaaattaacgtatcaattgcagcacaacaa
ggtggattctttttttggatgcaccaggtggcactggtaaaacctttctcatctcactgatacttgcg
cgcattcgatcacaaaatcatattgcattggccattgcttcatcaggcattgcagcgacgttacttgat
ggtggacggactgcgcattcagcacttaagttacctttgaatgttcatacaaatcccgaagcaatgtgt
aacataaagaagcattccggcatggctgaagttttgagaaaatgcaaaattattatttgggatgaatgt
acaatggcccacaagtctcaggcattgcagcgacgttacttgatggtggacggactgcgcattcagcac
ttaagttacctttgaatgttcatacaaatcccgaagcaatgtgtaacataaagaagcattccggcatgg
ctgaagttttgagaaaatgcaaaattattatttgggatgaatgtacaatggcccacaagcattcgcttg
aagctcttcgacaggtccctgaaggatatcaaagataatactaggcttttcggtggtgctctactgcta
ctgtctggtgatttcaggcaaacattgccagtaattccacgtgcgacatatgcagacgaagtaaacgca
tgtttgaaggaatcttatctatggcggagtgtcagtaaattgagccttactatcaatatgcgcgttcaa
ctacaaaatgatccattagcgtcaggattctctgaacaattgttagacattggcaacggtaaaattcaa
ctgtatgaagatacccaatttattaaatttccagagaacttttgcaacatggtggctaccaaagatgaa

ttaataaatagtatctttccagatttaagatataactatactaatcatgaatggctacgagagcgagct
attttagctgcaaaaaatttagatgtagacgccatcaattttaaaatacaacagtcattgcctggtaat
gaaattacatttaaatcaattgacaccgttgttgatcctgacgaagtggtcaactatccagtagaattt
ttgaattctttagatttacctggaatgccaccacataatttgcgactgaagattggctcacctataatt
ttgcttcgtaatttaaatgccccgagattgtgtaatggcacgcgattagttataaaaaaatcatgggc
aacattattgaagcgaatattttatctggaaaattccaaggtgaaattgtactttaccgcggatccca
atgattccttcagattcacctataccatttaaacgcttacaatttccgatccgtttggcatatgctatg
actattaataagtcacaaggtcaaacgatgacattttgtggcttagatttagaaaatccgtgtttttct
cacggccaattgtacgttgcgtgttccagagttggaaaaccatcgagtttgtttgtttatactcctcat
ggactaactaaaaatattgtacatccaatggcattacgataaattaagtttttgtaaattaaaaataaa
taaatatatatgttataaaatgttttgagtgcaagctttttcacctttcattaaaatgagttaaagttt
tcatttacgatgctgttatttatttaacaaagtaataacttttcaataataattttcatcaaagcggtc
caaattgtttcagctcattaaaaaaagtatgaaattaaagacgtgtgtacaggacaacgtctgtcgggt
ccgctag

>*Cotesia vestalis* (JZSA01002113.1: 8105-15507)

tcttaatatatatatttcttgtgtgcgtgtgtatttgactgaactcctcctaaacgactggaccaattt
tgatgaaatttttttgtgtgtgttcgtggagattcgagaatggtttagatttacagtttggtctactgga
aaatgtttttttcaattaatttcttatttataaggagttgttgattttggaatgttttacattagatccg
gcggacggcgctatcatcgcattcaatattattctatttcaattttagtttgtcctgacagatggtgct
acgattaatttgaaaaaaatttgttattcaattcatgtgctgattaaaatattaaatgtaattatttt
ttaaagagttttgaagtgtgtataaataattttgtgaattcagatgtttttttaattaatttttatttg
taaggagtttttgattttggaatgttttacatcagattcgacggatggcgctatcatcgcattcaatat
tattctattttaattttagtttgtcccgacagatagtgctacgatcaatttgaaaaagtatttcgttat
tttgtgttataattgtgttattcaagtgaagttgctaattaaaatatttaatgtaattagttttcgaag
agttctgaagtgtatataaataacttatacattttgtaaatttagtgcgtaatcgaagtgagtatattt
tttataaattatctttttttaatttacacgtgcgtttattaacaaccaacataaccttcaagtttatttg
tgtagttagaggattgaatttttttaattatttttattgacaattaattcatttttggagatattaaat
aaaaaaactcacaaaatgcgattacatgttctttatctaaaatttgtcataataaatcttttgagttaa
ttaattaaaaattcattatcaataagtaactcatattcattgtatattattttgcaatattttattttt
gtagaaacatgccacgtcttcgtggaagagcaagaaatattggtcgacgtactcagcacgcgcaattag
ttcatgatcatctattgaatagaacagtagaagaacattctatggataatgtaaatttaagagatcgag
ctgcatgtacacgtgcaaatgaaaatttagagcaacgtgctcaacttcttcgtgctaatacattaagac
aacgagaggcacgtcaacgagcgaccaacgcacatagagaacgtaaccaacagcgagtacaagataatc
gagcattggcacgagcattacttaatcgtctcgcgtttgaatatgatcctggaatagattattcatcac
atgcatttatcacgattggtagtatggacaaagagtgtcaacattgtcatgctttcaagtacaaaggtg
aatcagctggtttatgttgcgcatctagaaaaatatcactgtcaccgctaaaaccaccaccagaacctt
taaaaacgcttttagctggaatcacatctcaatcgaaattatttttacggaaaattcgtaaattaaatt
catgcttccaaatgacatcatttgcagcaacaaaaattattcataatggagatggccgtaattttgaat
ccacattcaaaattcaaggccaagtatatcaccaaattggttcgttacttccaatgcctgatgccgatc
caaaatttttacaaatttattttatggggaatgaagagcaacaatcacatacacgctgcgtttacaacc
atatagaacagatggaggaacgagaaattgtggacattttggaaaggtttttgcaaaaccataaccaat
tagtacaattgttcaagactctttctaacagactgcaaaacgataactatgtcattgttattaaagcag
acaaagtaccctatggagagcacgcaggcacatataatgttccaaccattaatgaagtggcagttgtta
tggctggtgacccatgtgaacgtagagacattcgcatacaacgcagagataatacgatgcaaataattc

aagacaatcatcgttcttacgatgctttgcagtatccgttaatattttgggaagcagaagacggatatc
atttaaatattaaacaacggaatccaactacaggtacaacttatagattatcttacaattttttttttg
aataaataattttttgtttatctcataattatatttatttacaggtgaagaactgaccaagaaagttagt
gccatgaatttctacgcatatcggttaatgattcgtgctaatgaagataataatattctccgctgtaga
cagctatttcatcagtacatcgtcgatatgtatgtaaaaattgaaagtgaaagattaaggtatataaaa
ttcaatcaagcaaaacttcgagctgaagagtatattcatttgcgtgatgcagtaatcggtaacgtagat
gcaacaaacgtcattaacaacatcggtaccacatatattctcccatcatcatatattggtagtccgcgt
catatgcaagaatatattcaagacgctatgacatacgtacgtgcatacggtcgaccagatctttttatc
acatttacatgtaatccaaattgggatgaaatcaaaaacttgctgttatcaggtcaaacatcgatgcat
cgccacgatatcactgcgcgtgtctttaaacaaaaattaaaatctttgatgaatttgatcacacattac
tcggtttttttgtgaaacacgttgttggctttactctgttgagtggcaaaaacgaggtttacctcatgcg
catattttgatttggttggtggataaagtacgcccagaagaaattgacaaaattatttcagcggaaatt
ccagatccgaatgttgatcaagaactgtttaacattgttactactaatatgattcatggtccatgcggt
actctaaacatgatgtcaccatgcatgaataatggaaaatgtaagaaacgttttccaaaaccatgtcaa
actgatactatcaccaatattgacggttatccatcttaccggcgcagagacgtagataatggcggtcaa
tcgtatgaattgcgactgtcaaacggtgtaagagtagatattgataatcggtgggtggttccatattcg
ccattactatgcaaaacctataaagcgcacataaacgttgaactgtgcagttcggtgaaatctatcaaa
tacatttgtaagtacgttcacaagggcagtgatgaagctatatttgctgttcaaaatgtaaatgacaat
gacgaaataacacgttatcaaatgggtcgatacataagtagcaatgaagctgtctggcgcatttttacg
tttcctttacatgaaagggatcctgcagttatacatttggctgtgcatcttgaaaacggacagcgtgtt
tattttacagaacagactgcactacaacaagcattaacggctccaaaaacaactcttactgaattttc
aatcttggtaatcgacaagatgttgttggtcaattcgcaaaaacattaatgtatactgatgttcctaaa
tttttttacatggaataagcaatcgaaaaattgggaaccacgaaaacgagggattccagtcccaggattc
gccgacatatttatgacaaatactttaggtcgactatatacagttcatcctaagcaacgcgaatgtttt
tttttttgcgtttgttattggttaatgttcttggaccgacatccttccaatatttacgaaaagtcaatgg
tattttatacgacactttcttcgatgcgtgtcgtgagttacatttattggatgatgataaccattggga
tctcacacttgcagatgcagcactgagctcttctccacaacaaattcgtcaattattttcaataatatt
aacaacgtgttttccgtctgaagcatctgctctctggaacaaatataaagactcaatgagtgaagatat
tttgcatcggattagaattactaatcaaaatcctgatattcaattctccgcagaaatatacaatgaggc
tttaattatgattgaggatatttgtattcttatttcaaacatgccactcatccattttggcatgccggc
gccgaaccgcccagcagtagatatcatcaacagtgatgttcaacgtgaacaccagtttgataagacttc
tttggctacttttgttgctaataatgaacaattgcttacagctgaacaacgaaatgtatatgatcaaat
taacgtatcaattgtaccacaacaaggtggattctttttttttggatgcaccaggtggcactggtaaaac
atttcttatcgcactgatacttgcgcggattcgatctcaaaatcatatggcattggccattgcttcatc
aggcattgcagcaacgttacttgatggtggacggactgcgcattcagcacttaaattacctttgaacgt
tcatacaaatcccgaagcaatgtgtaacatgcacaagcattcaggcatggctgaagttttgagaaaatg
taaaattattatctgggatgaatgtacaatggcccacaagcatccgcttgaagctctcgacaggtccct
gaaagatatcaaaaataatgctcggcttttcggtggtgctctactgctgctgtctggtgatttcagaca
aacattaccagtcattccacgcgcgacatatgcagacgaaataaacgcatgtttgaaagaatcttatct
atggccaagtgtcactaaattatgccttactattaatatgcgcgttcgacttcaaaatgatccattagc
gtcaggattctctgaaaaattgttagacattggcaacggtaaaattcaattgtatgaaaatacacaata
tattcgacttccagagaattttcgcaacatggtggctaccaaagatgagttaataacaagtatctttcc
agatttaagacataattatactaatcatgaatggctgcgagagcgagctattttagccgcaacaaattt
agatgttgacgccatcaattttaaaatacaacagtcattgcctggtgatgaaattacatttaaatcgat

tgacactgttgttgatcctgacgaagttgtcaactattctgaagagtttctaaattcattagatttacc
tggaatgccaccacataatttgcgactgaaaactggctcacctataattttgcttcgaaatttaaatgc
atcaaaattgtgtaatggcacgcgattagttgtaaaaaaaatcatgggcaacattcttgaagccactat
tttgggtggaaaatttcaaggtgaagttgtactttaccacggatcccaatgattccttcagattcgcc
tataccattcaaacgtttccaatttccaatccgcttggcatatgctatgactataaataagtcacaagg
ccaaacaatgacaatttgtggcttagatttagaaaacccatgtttttctcacggccaattatatgttgc
ttgttccagagttggaaaaccatcgaatttatttatttatacacctcagggtttaaccaaaaatattgt
acatccaatggcattacgataaattaagttttataaataaaaaaatatatttttttaaatgaaatagtt
tgagtgtaagctttttttacctttaattaaaacaagttgaagatttcattacacatactcacacgctatc
gattagtctaattgtaataaatttacatgtaatgacgttagatattgtcataacatttgaataataatt
ttcatcaaaatggtccagaatgttttagcttattaaaaaaagtttgaaattttcaaattaaagacgtgt
agacaggacaacgtctgtcgggtccgtgtatatgatcaaattaacgtatcaattgtaccacaacaaggt
ggattctttttttggatgcaccaggtggcactggtaaaacctttctcatctcactgataattgcgcgc
attcgatcacaaaatcatattgcattggccattgcttcatcaggcattgcagcgacgttacttgatggc
ggacggactgcgcattcagcacttaagttacctttgaatgttcatacaaatcccgaagcaatgtgtaac
ataaagaagcattccggcatggctgaagttttgagaaaatgcaaaattattatttgggatgaatgtaca
atggcccacaagcattcgcttgaagctctcgacaggtccctgaaggatatcaaagataatactaggctt
ttcggtggtgctctactgctactgtctggtgatttcaggcaaacattgccagtaattccacgtgcgtca
tatgcagacgaagtaaacgcatgtttgaaggaatcttatctatggcggagtgtcagtaaattgagcctt
actatcaatatgcgcgttcaactacaaaatgatccattagcgtcaggattctctgaacaattgttagac
attggcaacggtaaaattcaactgtatgaagatacccaatttattaaatttccagagaacttttgcaac
atggtggctaccaaaaatgaattaataaatagtatctttccagatttaagatataactatactaatcat
gaatggctacgagagcgagctattttagctgcaaaaaatttagatgtagacgccatcaattttaaaata
caacagtcattgcctggtaatgaaattacatttaaatcaattgacaccgttgttgatcctgacgaagtg
gtcaactatccagtagaatttttgaattctttagatttacctggaatgccaccacataatttgcgactg
aagattggctcacctataattttgcttcgtaatttaaatgccccgagattgtgtaatggcacgcgatta
gttataaaaaaaatcatgggcaacattattgaagcgaatattttatctggaaaattccaaggtgaaatt
gtactttaccgcggatcccaatgattccttcagattcacctataccatttaaacgcttacaatttccg
atccgtttggcatatgctatgactattaataagtcacaaggtcaaacgatgacattttgtggcttagat
ttagaaaatccgtgtttttctcacggccaattgtacgttgcgtgttccagagttggaaaaccatcgagt
ttgtttgtttatactcctcatggactaactaaaaatattgtacatccaatggcattacgctaaattaag
tttttgtaaattaaaaataaatatatatatatgttataaaatgttttgagtgcaagctttttcaccttt
cattaaaatgagttaaagttttcatttacgatgctgttatttatttaacaaagtaataacttttcaata
ataattttcatcaaagcggtccagattgttcagctcattaaaaaaagtatgaaattaaagacgtgtgta
caggacaacgtctgtcgggtccgctag

>*Homalodisca vitripennis* (JJNS01160820.1: 13417- 8013)

atattttcttgtgttcatgtgtatgtttctgaactccacttaaacgactgcaccgatttaaatgaaatg
ttgtgtgtgttttcaatggaattcgagaatggttgcgattcacaatttggtccagtggaaaattatttt
aaattaatttttcatttgtaagtagttgaatattttagggtgttttacattggatccggcagactgcac
tacgagtcgaaatacaaagtgaactgatgtttaacagctgttaacattttcagctaaagtaaagaaaag
aaatgctaacatcgcatttaatattattctattttaattttagtttgtcccgacagtcagtgctttgat
caaattgagaaaaatatttccttactttgtgatattattgtgttattgaaatcgtgtactaattaaaat
attacatcaaaatgtaattagtatacgaaaagttttgaagtgtgaataaaaaactatacattttgtgaa
tttagtgcggaatcgaagtgagtatgtttcttataaattttctattaaatttacacatacgtcaattaa

caaccaacctaaactataaatttgtttgtgtagctcattgatttcatatcgaagttttcaattgtttt
aataaaaattatttacttttggaaatattaaatgaagaaagcacaaatctaattacactttttttctaa
atttcttacaaaaatctttggagtaaattaattaaaatttcgtcttcaataagaaacttattcgacgt
gtattattttatagtactttatttttacagaaaaatgccacgtcttcgtggatgagcaagaaatactgg
tcgacgtactcggcatgcagatttggtccgtaatcgtcgattaaatagaacagttgaagaacacttgac
ggataatacaaatttcagagaccaagttgcatgtacacgtgaaaatgaaaattcagagcaacgcactca
acgtcttcatgccaatagaataagacaaccagaggtacgtcaaagagtgaccgacgcggatagagaacg
taaccaacgcaagactgcaagataatcgagctttggcacgaacacttaaatccgagataatcgaaagat
aaaattaatttccgacaagaaaggtccagtcacgttttctcgtatctctaatggttaagctacaaaac
gccctcagagtccaaagtttggttgaatccggaatgaatcaataaaataggtctactttaaacttccag
atccctaaactaatgctcggacttaacctgtgtccgaatttcggttatccccgcatttagccaacttcc
caattaggggcctgggggcatagcccccagcgagccgaatgcgagtcctgaaatagactattcgtcaaa
tgcattaatcacgatctgtagtatggacaaagaatgtcaacattgtcagcttttaggtataaaggtgaa
tcagctggtttatgttgcgcatctggaaaaatttcactgccactgctaaatccaccatcggttcctttt
taaaacacttttaggtggaacaacatctcaatcaaacttgtttttggggaaaattcataagttcatgct
tccaaatgacctcatttagagcaacaaaaattgctcataatgaaggtagagcacgcaggcgcatataat
gttccaactcttagtgaagttgcagttgttatggctgatgacccataacgtggtgaacgtcgagacata
cgcatacaacacagagataatacgatgcaaataattcaagacaatcatcgttatcacgatgctttgcag
tatccgttgatattttgggaaggagaagatggatatcatttaaatattaaacaaaggaatccaactaca
gttaaaccttatagattatcttacaatttttttataatcaataatttctgtttatctcataaacatata
tttatacacaggtgaagaactgataaagaaagttagtgctataaacttctatgcatatcgaataatgat
tcgtgctaatgaagacaacaacattctccgatgcagacaactatttcatcagtacattgttgatatgta
tgtaaaaatcgaaagtgagagattacgacatataaaatttaatcaagcaaaacttcgtgctgaggagta
cattcatttgcgtgacaccgtaatcggtaacgtagatgcaacaaatgacatcaacaacatcggtactgc
atatatcctaccatcatcatatattggtagtccacgtcatatgcaagaatactttcaagacgccatgac
ttacgtacgtgcataaggccgaccagatctttttattacatttaagtgtaatccaaactgggatgaaat
taaaaatttgctgttgtcaggtcaaatatcgatgcatcgccttgatatcactgcacgtgtcttcaaaca
aaaatcgaaatctttgataaatttaattccatatcaatcggtatttggtgaaacacgttgttggcttta
ctctgttgagtggcaaatacctcacgcgcatattttgatttggttggtgcataaagtacgcccagatga
aattgacaatattatttcagcggaaattctagatccgaatgttgatcaagaattgtttgagattgttac
tactaatatgatcagtggtccatgtggtactctaaagataatgtcaccatgtatggacaatggaaaatg
tacaagacgttttccaaaaccatgtcaaacaaatactatcaccaatattgacggttatccattctaccg
acgtagagacatagacaatagcggtcaatcataagaattccgtctgtcaaattgtgtaagagtagatat
tgatcatcgctgggtagttccttattcgccattgttgtgcaaaacctataaagcgcacataaacgtcga
actatgcagttcggtaaagtctatcaaatacatttgtaagtatgtttacaagggcagtgataaagctat
attcgctgttcaaaatgtaaacgaaaatgacgaaataacactttcaaaaatgggtcgatacgtaagtag
caatgaagctatttggcccattttacgtttcctatacataaaaggtatcctgctgttatacatttgtc
tgtgcatcttgaaaacgggcagcgtgtttacttaacagaacagagtgtactgcaataagctttaacggc
tccaaaagcaactcttgttgaattttcaatctttgtagtcgacaagatgttattgttggtatattcgc
aaagacattgatgtatactgatgttcctaaatttttacatggaataaacaatcaaaacaaattgggaa
ccacggaaacgaggaattccagtccaggatttgccgacatattcatgacaaatactttaggtcgattat
atacagttcattctaagcaacgcgaatgtgtttttgcgtttgttattggttaacgttcctggatcgaca
tcctttcaatatttgcgtacaatcaatggtactttatacggcactttcttcgaagcggtcgtgcagcac
tgagctcatctcaacaacaaatcaattatatacaataatattaacaacgtgttttccgtatgaagcatc

tgctctgtggagcaagtataaagactcaatgagtgaggatattttgcatcggattaaaattaataatca
aaatcttgatattgcattctccgcagaaatatataacgagtcattaataatgattgaggatatttgcat
tcttatttcgaacatgccatttatctatttcggcatgccagcgccgaaccgcccagcagtaaatatcat
caacagcgatgtaaatattatcaacgtgaacatcagttcgatcagacttctttggctactttgtggcta
attatgatcaattgtttactgctgagcaacgaaatgtatatgatcaaattaacgttacaatttcagcaa
aacaaggtgaattcttttttttttggacgcaccaggtggcactggtaaaacattcctcatctcactgat
acttgcgcgcattcgatcacaaaatcatattgtattggcaattgcttcatcaggcattgcagccacctt
atttgatggtggacggactgcgcattcagcacttaaattacctttaaatgttcatacaaatcccgaagc
aatgtgtaacataatgaagcattcaggcatggctgaagttttaagaaaatgagaaattattatctggga
tgaatgtacaatagtccacaagcattcatttgaagctctcgataggtccctgaaagatatcaaagataa
tacaaagcttttcggtgatgctctattgctgctgtctggcgatttcagacaaaaattaccagttattcc
acgtgcgacatatgcagacaatgtttgaaagaatcttatctatggcgaagtgtcagtaaattatgcctt
actattaatatgcgcgttcaacttcaaaatgatccattagcgtcaggatttgctgaacaattattagat
attggcaacggtattttaaacatatttatactaatcatggatgtctacgagagcgagctattttagccg
cgaaaaacttagatgttaacgccatcaacttcaaaatacatcagccatatcctggtaatgaaattacat
ttaaatctattgatactgttgttgatccagacgaagttgtcaattgctttagagagtttttaaattcac
tagatttaactggaatgccaccacataattttcgactgaagattggctcacctataacttttcttcgaa
atttaaataccccgaaattatgtaatggcacgcgattagttataaaaataatcatggacaacattcttg
aagccactatttaaagcgggaaatttcaaggtgaagttgtacttctaccacggatccaaatgattcctt
cgagttcacttataccatttaaacgtttacaatttccgcttggcatatgctgtgactataaataagtca
caatgacaaacaatgactatgttgcgtgttccagagttgaaaaaccatcggattggtttgtttatacgc
ctcaaggacaaatattgtttacggatatcaaatagcattacgataaattaaatttttgtaaattaaaaa
atatatttgttatacaacagtttaagtacaagtttttccacttttaataaaaaggacttaaaatttgt
atttaacactgcttctagatttcattccactcatacactatacacatactcacacgctatcggttggtt
gaattgtgataaatgtgcatgaaatgacgttagatattaaaataacgttttaataatagttttcaccaa
aacgatccagatcgatacagcttattaaataaaagtttgaaattttcgaattaaagacgtacgaacagg
acaacgtctgtcgggtctgctag

>*Athalia rosae* (AOFN01007542.1: 24588-16524)

tcttaatatatatatttcttgagtgcgtgtgtatgtgactgaactcctcctaaacgactggaccaattt
tgatgaaatttttttgtgtgtgttcgtggagattcgagaatggtttagatttacagtttggtctactgga
aaatgtttttttcaattaatttgttatttataaggagttgttgattttggaatgttttacattagatccg
gtggacggcgctatcatcgcattcaatattattctatttcaattttagtttgtcccgacagatagtgct
acgataaatttgaaaaagtatttcgttattttgaattaaaattgtgttattcaagtgaagttgctaatt
aaaatatttaatgtaattagttttcgaagagttgtgaagtgtatataaataacttatacattttgtaaa
tttagtgcggaatcgaagtgagtatattttgtataaattatctttttttaatttacacgtgcgtttatta
acaaccaacataaccttcaagtttatttgtgtagttagtgaatttttttaattatttttattgacaatt
aattcattttggagataataaaaaaaaaaaatcacaaaatgcgattacatgttctttatctaaaattt
gtcataataaatctttagagttaattaattaaaaatacattatcaataagtaactcatattcattgtat
attattttgcaatattttatttttgtagaaaaatgccacgtcttcgtggaagagcaagaaatattggtc
gacgtactcagcacgcgcaattagtacatgatcatagattgaatagaacagtagaagaacattctatgg
ataatgtaaatttaagagatcgagctgcatgtacacgtgcaaatgaaaatttagagcaacgtgctcaac
gtcttcgtgctaatacattaagacaacgagaggcacgtcaacgagcgaccaacgcagatagagaacgta
accaacagcgagtacaagataatcaagcattggcacgagcatcacttaatcgtcgcgcgtgtgaatatg
atccagaaatagattattcatcacatgcattgatcacgattggtagtatggacaaagagtgtcaacatt

gtcatgcttacaagtacaaaggtgaatcagctggtttatgttgcgcatctggaaaaatatcactgccac
cgctaaaaccaccaccagaacctttaaaaacgcttttagcaggaatcacatctcaatcgaaattatttt
taaggaaaattcgtaaattaaattcatgcttccaaatgctctgacatctgacacatgacaaaaattatt
cataatgaagatggccgtaattttgaatccacattcaaaattcaaggccaagtatatcaccaaattggt
tcgttacttccaatgcctgatgccgatccaaaattttgcaaatttatttcatggggaatgaagagcaa
caatcacataaacgctgcgtttacaaccatatagaacagatggaggaacgagaaattgtggacatttg
aaaaggtttttgaaaaaccataaccaattagtacaattgttcaagactctttctaacagactgcaaaac
gataactatgtcattgttattaaagcagacaaagtaccctatggagagcacgcaggcacatataatgtt
ccaaccattaatgaagtggcagttgttatggctggtgacccatgtgaacgtagagacattcgcatacaa
cgcagagataatacgatgcaaataattcaagacaatcatcgttcttacgatgctttgcagtatccgtta
atattttgggaaggagaagacggatatcatttaaatattaaacaacggaatccaactacaggtacaact
tatagattatcttaaaaattttttaggaataaataattttgtttatctcataattatatttattgac
aggtgaagaactgaccaagaaagttagtgccatgaatttctacgcatatcggttaatgattcgtgcaaa
tgaagataataatattctccgctgtagacagctatttcatcagtacatcgtcgatatgtatgtaaaaat
tgaaagtgaaagattaaggtatataaaattcaatcaagcaaaacttcgagctgaagagtatattcattt
gcgggatgcagtaatcggtaatgtagatgcaacaaacgtcattaacaacatcggtaccacatatattct
cccatcatcatatattggtagtccgcgtcatatgcaagaatatattcaagacgctatgacatacgtacg
tgcatacggtcgaccagatctttttatcacatttacatgtaatccaaattgggatgaaatcaaaaactt
gctgttgtcaggtcaaacatcgatgcatcgccacgatatcactgcgcgtgtctttaaacaaaaattaaa
atctttgatgaatttgatcacacattactcggtttttggtgaaacacgttgttggctttactctgttga
gtggcaaaaacgaggtttacctcatgcgcatattttgatttggttggtggataaagtacgcccagaaga
aattgacaaaattatttcagcggaaattccagatccgaatgttgatcaagaactgtttaacattgttac
tactaatatgattcatggtccatgcggtactctaaacatgatgtcaccatgcatgaatgatggaaaatg
tacgaaacgttttccaaaaccatgtcaaactgatactatcaccaatattgacggttatccatcttaccg
gcgcagagacgtagataatggcggtcaatcgtatgaattgcgactgtcaaacggtgtaagagtagatat
tgataatcggtgggtggttccatattcgccattactatgcaaaacctataaagcgcacataaacgttga
actgtgcagttcggtgaaatctatcaaatacatttgtaagtacgttcacaagggcagtgataaagctat
atttgctgttcaaaatgtaaatgacaatgacgaaataacacgttatcaaatgggtcgatacataagtag
caatgaagctgtctggcgcattttacgtttcctatacatgaaagggatcctgcagttatacatttggc
tgtgcatcttgaaaacggacagcgtgtttattttccagaacagactgcactacaacaagcattaacggc
tccaaaaacaactcttactgaatttttcaatctttgtaatcgacaagatgttgttggttaattcgcaaa
aacattaatgtatactgatgttcctaaatttttacatggaataagcaatcgaaaaattgggaaccacg
aaaacgagggattccagtcccaggattcgccgacatatttatgacaaatactttaggtcgactatatac
agttcatcctaagcaacgcgaatgtttttttttgcgtttgttattggttaatgttcttggaccgacatc
cttttaatatttacgaaaagtcaatggtattttatacgacactttcttcgatgcgtgtcgtgagttaca
tttattggaggatgataaccattgggatctcacacttgcagatgcagcactgagctcttctccacaaca
aattcgtcaattattttcaataatattaacaacgtgttttccgtctgaagcatctgctctatggaacaa
atataaagactcaatgagtgaagatattttgcatcggatcagaattactaatcaaaatcctgatagtca
attctccgcagaaatatacaatgaggctttaataatgattgaggatatttgtattcttatttcaaacat
gccactcatccattttggcatgccggcgccgaaccgcccagcagtagatatcatcgacagtgatgttca
acgtgaacaccagtttgataagacttcgttggctacttttgttgctaataatgaacaattgcttacagc
tgaacaacgaaatgtatatgatcgaattaacgtatcaattgcagcacaaaaaggtggattcttttttt
ggatgcaccaggtggcactggtaaaacctttctcatctcactgatacttgcgcgcattcgatcacaaaa
tcatattgcattggccattgcttcatcaggcattgcagcgacgttacttgatggtggacggactgcgca

ttcagcacttaagttacctttctacaacacatgcattaaaaactcgtgtttttgccctcctcggcggg
atggaagtcacagttttcggacgagcgggcaaaaaacttgccagcgggcaaaaaactctggcagcaggg
aaataaactcgccgccgggaaaaaaactctgtccgccgggaaaaaaactctcgtagccgtgtaataaac
caagaaagtgctgcccctagcgtagaactatattgagtacgatgttttcatgacgagttgtatactaac
cgaaggactaaggcgccgttcgaacggttttaagtgttctaacctcaaaaatggagtctactacagaaa
atgaccgtccaactacgcctcctgatatacgtgacgcagcggataaagtttcatttcacctgttgccgg
ataaatcgaaagaagtttatgaatccgcctacaaggcatacaccgactggagaacgtccaaaggcgcga
attcgtcttcggaatcagttttactggcatatttcgatcagatgtcgcaacaatataaacctacaacta
tctggtcgttatactcgaagctcaaaagcaccatcaatatcagagaaaaagttgacataagcagctaca
aaacattatcggctttttttaaaaaaacagtcacgtggatttcgcagcaaaaaatccagcgtatttacat
ccgaggaagtacagtctttttttgacaagggccccagacgacaaatatcttgcaacaaaggtaaaattac
agtatatacatataacatacactctacacacctacacctacactctatttacaatcaactgtgttattt
acagatcgttctaatttttgggattttcggcgcattgaggaccgatgaattaacaaacattcaagtacg
cgatatgaaaaagcaagggaatctactgcaggtccaagttcctaaaactaaaacgatgctaccacgatc
tttcataattcctgaggattgtgtggagtacgttacacgatatcagtctctccgtcctgaaaatgtacc
ctccggccgcttcttcataaattaccaaaatggtaaatgtactgcgcaggtgattggaaaaaacaagtt
tgccggtatgccgaaagaaatcgctgagtttcttgatttacctgatgctgcatcatacaccggccatac
ttttcgccgtacctccgcaacaatgcttgttgacaatggagcgaacatggaaacgctgaagaggcatgg
tggctggaaatcaagcactgttgcggagggatacatagcagaatccttaggaaacaaaaaaaatatcgg
ggatatgataatgtcttctctgaatttgccttcaacagcaacctcagccgacaaccgcagtgtcaaaat
atcatcgccaaagcgtcctaaaatggacgattcaataaacgcagtaccttctacagcgaaagtgcattg
tggcccggagcaaaaagtattcactttcgaaaattgtacaaatgttacaattaattacacagggaatcc
gggaaatcctctgtaattttctattagctcattagctgtgataaaacgtaacaaaaccactcttggctc
gagagtaaggttgtcagaggtttttagatgattggatgatattaataataagaaaaactgatttgtgat
gataaacatatcctgaatataaaatatataagtctaaatatacgtacctactattagagataagaaact
gatttcaagttaagcttgattgcagtacctatgcatactattaaaattagggaaaattctgtgattcca
agagaataagagtgaacatagtttaagtttgatactatcataaatataaaaagaactttgtgatttcaa
catcaataaaaatcatcttgaaaatataagaaacaaaaatatgaaaatatttaaaatataaaatatata
atcctgatataattgaatatgaaaaaacagatgtaaggttttcattttgctagaaagattttttatgaaa
catcagatctttgataatgtttttagtcgggggctttgcccccccggcccccccacgggggcttcgcccc
tcgaccccaccaggggctccgcccctggaccccgccgtcacgcacagtggtctatcacagttcagtttt
tcaaggatgtagtaactatttgatcgcatttctgcaaagtccgaattatgcacaccttcggcggttttt
taggtcattttatcgtagtcgggtaagaagtagtatatgccacacgggaggagcggattttcaccttgt
gctcgtgcaaacctcgctttgtcgtaacttaacctgccaatgcatgagtaattcgtaatttccaatatg
tatttttcactcgtttttaatgcatgtgttatagaaaatagtatatgcaccacgggaaagggcaagcgt
tttgtctcgcgcgatgtttgccgaactcgtcttcactctcgggcgccttcggcgccctcgacttacaac
tcgttctgcaaacatcggcgctcgaccgaaaacacatgcttccttccctagatgcataatatactattg
aatgttcatacaaatcccgaagcaatgtgtaacataaagaagcattccggcatggctgaagttttgaga
aaatgcaaaattattatttgggatgaatgtacaatggcccacaagcattcgcttgaagctctcgacagg
tccctgaaggatatcaaagataatactaggcttttcggtggtgctctactgctactgtctggtgatttc
aggcaaacattgccagtaattccacgtgcgacatatgcagacgaagtaaacgcatgtttgaaggaatct
tatctatggcggagtgtcagtaaattgagccttactatcaatatgcgcgttcaactacaaaatgatcca
ttagcgtcaggattctctgaacaattgttagacattggcaacggtaaaattcaactgtatgaaaatacc
caatttattaaatttccagagaacttttgcaacatggtggctaccaaagatgaattaataaatagtatc

tttccagatttaagatataactatactcatcatgaatggctacgagagcgagctattttagctgcaaaa
aatttagatgtagacgccatcaattttaaaatacaacagtcattgcctggtaatgaaattacatttaaa
tcaattgacaccgttgttaatcctgacgaagtggtcaactatccagtagaattttgaattctttagat
ttacctggaatgccaccacataatttgcgactgaagattggctcacctataattttgcttcgtaattta
aatgccccgagattgtgtaatggcacgcgattagttataaaaaaatcatgggcaacattattgaagcg
aatattttatctggaaaattccaaggtgaaattgtacttttaccgcggatcccaatgattccttcagat
tcacctataacatttaaacgcttacaatttccgatccgtttggcatatgctatgactattaataagtca
caaggtcaaacgatgacattttgtggcttagatttagaaaatccgtgtttttctcacggccaattgtac
gttgcgtgttccagagttggaaaaccatcgagtttgtttgtttatacttctcatggactaactaaaaat
attgtacatccaatggcattacgctaaattaagtttttgtaaattaaaaataaataaatatatatgtta
taaaatgttttgagtgcaagctttttcacctttcattaaaatgagttaaagttttcatttacgatgctg
ttatttatttaacaaagtaataacttatcaataataattttcatcaaagcggtccagattgtttcagct
cattaaaaaaagtatgaaattaaagacgtgtgtacaggacaacgtctgtcgggtccgctag

>*Timema cristinae* (MSSY03008002.1: 97200- 90981)

tcttaatatatataattcttcagtacgtgtgtatgtcactgaactcctcctaaaccgctagaccgattt
tgatgaaatttttgtgtgtgtttgagcgggttcatggatggttttgattcacaattgaactcggtaga
tggcgcttcagatcgggttttaggatttatttttattctttagggaatttatttgaggggatttttatt
aaagatttttgcatatcagaacatgtaacctcacaatttcactccccaccaccaccaaccaatattta
taataatgaatataatatgaataattgaccgttaaaaccttctgaatagtgaataattgaacttctgag
aataattcatagttatgtttgtgtaccataggtggcattgatttcnaattctgtggtatttgacgtacg
nataattgaacttctgagaataattcatagttatgtttgtgtaccataggtggcattgatttcgaattc
tgtggtatatgacgtacgcatcaatacatgtgcaagtctcttgcgtcgtggcttggcgttgttttggca
tttcaatttctgtacgatgtgtttagatatttcatgtatcacaatcgcgtgaatattgagtgtattttt
catcaagtgtactcaaatacatacattgggaaaatgtgtaaggtgctgtgttgaagtgaatatcttaca
tgatcttccataaatttaaattatatacatgcgtattcaatgacaatcaatataacctgtaaatttcta
ttttaagtgcttatttgtgtcgatgattttataaaatttacggagtgttaaacatctcattggaaaaaa
aaacatgtaatccattaaattcaaatataagttgcttaaattttggtgttgtgtgtttattaaagaaca
atgccacgtcttcgtggacgaggaggaaatattggtcgacgtacacagaatttacaaatagtccacacc
tgtcggttaaattgaatggcagaagatcaattgacggacaatgcaaatttaagagtacaggttgcaaac
acacgcgcaaatgaaagtcaacagcaacgcaatgaacgccttcgagctaatgcattgagacaaagacag
gcgcgtcaacgagccactgatgcacaaagagcgcatgaacaacagcgcttgcaagtacatcgcgcattg
acacgcgcatcacttctccgtcttgagttcgaatatgagtcggacatcgactattcgacacattcacaa
attgtcattggcgctatggacaaagaatgccaacattgccatgcattcaaatacaaaggtgaatcagcc
agtttgctgcgcgtctggaaaagttgtacttccaccattgaattcgccgccagagccattgaaaacact
attggctggagccacacctcaatcaaaattgtttttgcgtaaaatttgcaaattcaattcttgcttcca
aatgacatcattcggaacaccaaaaatcgttcagaatgaggatggttgcaatttgaatcaacgtttaa
gattcagggccaagtatatcatcaaataggttctttaatctcaaagcctgatgccgatccaaaattctt
acaaatctacttcatggtcgatgaagtgcaacaaataaaagtacgctgccagtacaaccacatcgagca
gatggaggagcgagaaattgtggtgactttggaactgtttttgcgaaatcacaaccagttggtccagtt
gttttatacagtttcaaacagactgcaaaatgacaactacatgattgtcttcaaagcagacacagtacc
atttggagagcatgcgggctgatacaattcaccaaccgttaatgaagttgcagttgttatggttgtcga
catatatgaacgtcgagatatatggatccaacacagtgataacacggttcacacaattcaagacagtca
tcgttcttacgatgctttgcaatatctgttgatattttgggaaggagaagatggatatcattttaacat
caaacaaataaatccaatgacaggtacaatctacatattatttactttttctttttctgtaagattaat

ttatttttgtcttatttatatttttactttgcaggtacagaaaccaacaataaagtgagagcgatgaac
ttctacatatatcggttgatgattcgagccaatgaagacaacaacattctccgatgccgccagctgttt
catcaatacatcgttgatatgtatgcgaaaattgaaagcgagcgattacggtacatcaagcataatcaa
gcgaagctgcgttctgaggagtacattcatttgcgtgatgctattgtcggtaacgttgatggaaccact
aatagcaacgacatcagtaccgcatatattcttccatcatcatatattggtagtccgcgtcatatgcaa
gaatacattcaagacgccatgactttcgtgcgcgcgtacagccgaccagatttgttcattacatttacg
tgcaatcctaactgggatgaaattaaaatgtttttattgccaggtcaaacaacaatgcatcgccatgat
atcactgcacgtgtcttcaaacaaaaattaaaatcgttgatgaacttgattacacatcactctgtattc
ggcgaaacacgttgatggctgtattctgttgaatggcaaaaacgaggtttgccacatgcacatattttg
atttggttcgtcgacaaagtacggtcagaagaaattgataaaattatttcagcagaaattccggatatg
aatatcgataaatagttgttggacatttttttctgctaacatgatccatggtccatgtggcgctctaaac
attacggcgccgtgcatggacaatggaaaatatacgaaacgtttctcaaaagcgtgtgtaaacgatact
gtcgctaatatcaaccgttatccattgtatcgtcgtagaggcacagataatggtggacaatcatatcaa
ttgcgcatgtccaacagtggaacggttgacattgacaattgctgggtggttccgtattcgccattgctg
tgcaaaatctacaaagctcacattaacgttgagttctgcagttcggtgaagtccataaaatacatttgc
aaatactttcataagggcagagatatggctgtatttgctgttcaaaatgtaaatgataacgacgaaata
aaacgttatcaaatgggccgatatatgagcagcaatgaagcagtttggcgcattttgagtttttcccata
cacgaaaacaccctgctgttatacatttggccgtgcaccttgaaaatggacagcatgtttatttcacag
aagagactgctctacaacgagcattaactgccccaaaaaccacactgagattttcgaactatgcaacca
accagatattttcggtcgattcgcaaagacattaatgtgccacaattttgacgtgaaacaaacggtag
aaaaagtgggacccacaaaagcgaggcctttcagtccaaggatacgacggaatatttatgggaaataca
ttaggccgattatacacagtccatccaaagcaacgcgaatgcttcttcttgcgtttgttgttggttaac
gttcctggacctacgtccttccaatactttcgaaaagtcaatggtattttgtacgacacgtaccacgat
gcgtgtcgcgaattgcatttgctggaagataacaaccattgggatctcacacttgcagatgcagtacag
agttcctctcctcatcaaattcgccaattattcgcaatattattgacatgtttaccgttgcaagcttct
gttatctgggataaatataaagattccatgagcgacgacattctgcatcgtattcggaccgctgatcaa
gatccaaatatcgattttgtcgcatgaaatatataacgagtcgttaataaagattgaggatatttgcat
tctcatttctaatatgcctctccatcatttcggtacgccatcgcctaaccgcccagcaacagacatcat
taacagcaatattctgcgtgaacaacattttgatgcggcctctttgactactttcgttgtaaataatga
gcaactgctcactgctaaacaaaagaacatatacgatcgaattaatttgtcaattgcagcacaacaagg
tggattttttttttggacgtaccaggcggcactggcaaaacattcctcatatcactgatactagcgcgc
attcgatcacaaaattatattgcgttggcaattgcttcatcaggcattgcagcgactttgcttgatggt
ggacggacatcacattcagcactcaagttgccattgaacgttcacacaaatccagacgtaatgtgcaat
ataaagaagcattcaggcatggcaaaaattttgcaaaaatgcaaaattattatctgggatgaatgcact
atggcccacaagcattcacttgaagctctcgatagaacgatgaaagatctcaaaacgataataccagac
ttttctgtggcgctctactactactgtccggtgattttagacagacattgccggtcattccacgcgcta
catatgcggacgaagttaatgcatgtttgaaacaatcatacctaaggcgaaatgtcaatacattatgcc
ttaacataaatatacgtgttcaagtgcaaaacgatccattggcactgtcattctcagaacaattgctca
acattggcaatggaaaaattctattgcatggaaatacaccatgaataaaattgccagacaattttgca
acatggtagccatgaaagatgctttgattgaaagcataatcccagatttgcgcgctaattacaataatc
atgcgtggctatggaatgagctattttagccgcagaaaatatcgatgttgaagctattaatttcaaaat
acaacagttattgcctggcaatgagatatcgtttaaatcgatcgacactgttgtcgatcctgacgaagc
agtcaattatcctgtagaatttttgaattcactggttttaccgggaatgccaccacatagcatgccatt
aaaaattggttcaccaatcattttgcttcggaatttaaattcaccaaagctatgcaatggcacgtgatt

gttcataaaaaaaattatggttaatgttcttgaagctaccattttgaacggcaagtttcaaggtgaggt
tgttctcctgccacggatcccaatgatcacttcagacgcgcccattccattcagacacttgcaatttcc
aattcgcttggcattcgcaatgactattaataagtcacaaggccaaacaatgtccatttgtgggttgga
tctagaaaatcactgcttctcacatggtcaattatatgttgcgtgttctcgcgttggcaaaccatcaag
tttgttcatttatacacctcatgtattaacgaaaaacattgttcatcaactcgcattaagataaaaaca
aaacaaactttattgattttgtcggaccgtttttataacattaaaaccttaggtataagaggaggtga
ggtttctacgccgctgactgtacgtataatcgaaatagtatgtacttcgtacaacaagtacatactatt
tagatggtacgtatataggcaagttgttctgcttaagcccgtaaaggttctaggcccccaacagtatat
gttttgcactatgaaaatagaaacaattaaaaagtaatttgacatttgtcatatttgaaggtccgtcaa
taaaataaaattagctgtacccggcatacaaatttgtaagcaagcttcgcaatttttttcgatacagct
gatatttgtggagttgtgaatgaaattgcaaaaattgtagggatcactattccatatattattattttt
aataatggatacaatgtatgtataagtgtctagcagttcctattctaagtaaaatttattagtttcaga
aacagacagacagaatgggaaagcattgccattactgtgaaacaaagtatttgtaatgtactattgtct
caatatagttgattttgaggaaagaaaagatgttttattttgtatgttgtcttgacaaaccctgaattt
ttaatatttgtgtaactcantaattngagttcattgtgaatttgtaattgtgatgatcacatagtgttt
gttttattatcgaaatcaattatgccacccaaacaaaaaaccaacgttcgggcaggacaacgtctgtcg
ggtccactag

NNNNNN Rep motif NNNNNN Helicase domain

Data S2. The reconstructed potential autonomous *Hel1 Helitrons*.

>*Parasteatoda tepidariorum* (5608 bp)

tctttatatatatatttcctctgtgcgtgtgtttgtcactgaactcctcctaagcggctggaccgcttt
aaataaaattttctgtgtaccttcagaaggattcgagaatggtttaaattcacaattaggtccaatatc
aatttttttagttaatttttttattgagtgggaaaaattctacttcttaaaagttatacttcttatgcga
cttccatcaagaaagcgttaaacgtttaaagctacattttcattggctgggaaatcacgtggtaggatc
cagttttccctcattcatttccatattgttttggttctcactagcataaaaataataaaagtcataaaa
ctttttttttctgtaaatattttttttagtttgttttgatacacatataatttaatagggagcaggaaaa
agagtcgttgaatagaatttgtaatctcgtgttattattgtgtttgaaaagttttgaagtgtgcatttt
aaaaatgtaaaaaactacgcattttgtagattcagtgcggaattgaagtgagtattttgtataattta
gatcgaatttatacgtgcgtaggttaacaaccactctaacctataaatttatttgtgaagctaattgat
ttcatataaaactttttcaattgttttttatttaaaatttcttatttgaaacagtaaataaagaaaaaca
aaaaaataaaattgcacatttttttatctaaatttggtacaataaatcttttcagtcctgttacttaaat
aaaatatcattttcaataaattactcatattgtttatgtactattttacagtattttgttttacaaaaa
aatgtcacgtcttcgtggaagagcaagaaatattggtcgacgtactcgaaacgcacaattggtccatga
tcgtcgattgaatagaacagttgaagaacacttgacggataatgtgaattcaagaaatcaagttgcatc
ttcacgtgaaaatgaaaatttagagcaacgcaatcaacgtcttcgtgcaaatgcattaagacaacgaga
gacacgccaacgagcgaccaacgaaaacagagaacgtaaccaacggcgaatgcaagataatcgagcatt
gatacgagcatcacttaatcgtcttatgtttgaatatgaccctgaaatagactattcgtcacatgcatt
aatcttaatcggtagtatggacaacgagtgtcaatattgtcatgctttaaagtacaaaggtgaatcagc
tggtttatgttgcgcatctggaaaaatttcacttccaccgttaaatccatcatcggaaccttttaaaac
attattagctggaaccgcatctcaatcgaaattgtttttgcggaaaattcgtaaatccaattcttgctt
tcaaatgacatcatttggagcaacaaaaattgttcataatgagggtggtcgtaattttgaatctacatt
taagaatcaaggtcaagtgtaccaccaaattggttcattgcttccaatgcctaatgtcgatccaaaatt
cttacaaatttactttatgggcgataaggagcaacaaactcatacacgatgcatatacaaccatataga
gcagatggaggaacgagaaattgtggacattttggaaacgttcttgcaaaaccataaccaattgatacg
attgttcaagactctttccaacagactgcaaaacgacaactacgccattgttattaaagcagacaaagt
aagcagcaacgcaggcacatataatgttccaactgttaatgaagttgcagttgttatggctggtgaccc
atgtgaacgtcgagatattcgcatacaacgcagagataatacaatacaaataattcaagacaatcattg
ttcttacgatgcactgcaatatccgttgatattttgggaaggagaagatggatatcatttaaatattaa
acaaaggaatccaacaacaggcgaagaactaatcaagaaagttagtgcgatgaacttctacgcataccg
gttgatgattcgtgaaaatgaagataataacattctccgaagcagacagctatttcatcaattcattgc
tgatatgtatgtaaaaattgaaagtgagagattgcgttatataaattttaatcaagcgaaacttcgttc
tgaggagtacattcatttgcgtgacgccataatcggtaacgtagatgcaacgaatgatatcaacaacat
cggtaccgcatatattcttccatcatcatacattggtagtccgcgtcatatgcaagaatatattcaaga
cgccatgacttacgtacgcgcatacggccgaccagatctttttatcacatttacgtgtaatccaaactg
ggcagaaattaaaaatttgctgttatcaggtcaaacatcgatgcatcgccatgatatcattgcacgcgt
gttaaaacaaaaattgaaatctctgatgaaattgattacacatcactcggtatttggtgaagcacgatg
ttggctttattctgttgaatggcaaaaacgaggtttacctcatgcgcatattttgatttggttggtgga
caaagtgcgcccagaagaaattgacaaaattatttcagcggaaattccaaatccgaatgctgatcaaga
attgtttgacattgtgactactaatatgatccatggtccatgtggttctctcaacatgatgtcaccatg
tatggataatggaaaatgtacgaaacgttttcttaaaccatgtcaaactgacactatcagcaatatcga
tggttatccatcttaccgtcgtagagatgtagacaatggcggtcaatcatatgaattgcgtctatcaaa
cggcgtaagagttgacatcgatagtcgctgggtggttccatactcaccattgctgtgcaaaacctataa

agcacacatgaacgttgagttatgcagttcggtgaagtctatcaagtacatttgtaagtacgttcataa
gggcagagataaagctatattcgctgttcaaagtgtaaataagaatgacgaaataacacatcatcaaat
gggacgatacattagtagtaatgaagctatttggcgaatttttacatttcctatacatgaaagggatcc
tgctgttgtacatttggctgtgcatcttgaaaatggacagcgtgtttatttcaccgaacagaatgcact
acaacaagctttaacagctccaaaaacaactcttactgaattttcaacctttgtaatcgacaagatat
tgttggtgaatttacaaaggcattaatgtatactgatgttcctaaatttttacatggaataaacaatc
aaaaagttgggaaccacggaaacgaggcactccagtcccaggatttgccgacatagttatgacaaatac
tttaggacgattatatacagttcttcctaagcaacgcgaatgctttttctgcgtttgttattggttaa
cgttcctggaccgacatccttccaatacttgcgaaaagtcaacggtgctttatacggcactttatttga
tgcgtgtcgtgagttacatttattggaggatgataaccattgggacctcacacttgcagacgcagcact
gagttcatctccacagcaaattcgtcaattatttcaataatattgacaacgtgttttccgtctgaagc
atctgctctgtggaataaatataaagactcaatgagtgaggatattttgtatcggattagaattactaa
tcaaaatctctgtcttgaattctccgcagaaatatataacgaggcattaataatgattgaggatatttg
ttttcttatttcgaacatgccgctcatgaattttggtatgccagcgccaagcagcagatatcatcaaca
gcgacgtgaacaccagttcgatatgacttatctggctacttttgttgctaacaatgaacgattgcttac
tgctgagcaacgaaatgatcaaattaacgtgtcaattgcagcacaacaaggtggatttttttctctgga
cgcaccaggtggcactggtaaaacattccttatctcactaatacttgcgctcattcgatctcaaaatca
tattgcattggcaattgcttcatcaggcattgcagcaaccttacttgatggtggacggacagcgcattc
agcacttaaattacctttgaatattcataccaatcccgaagcaatgtgtaacataaagaagcattcagg
catggctgaagttttgagaaaatgtaaaattattatctgggatgaatgtacaatggcccacaagcattc
gcttgaagctctcgacaggtccctaaaagatatcaaagataatactcggctttttggtggtgctctact
gctgctgtcaggtgatttcagacaaacattacccgtcattccacgtgcgacatatgcggacgaaataaa
cgcatgtttgaaagaatcttatctgtggcgaagtgtcagtaaattatgccttactcttaatatgcgtgt
tcaacttcaaaatgatccattagcgtcaagattctctgaacaactgttagacattggcaatggtaaaat
tcaattgtatgaagatacacaatatattcgactcccacagaatttttgcaacatggtgcctaccaaaga
ggagttaataaaaagtatctttccagatttgccacataattatactaatcatgcatggctacgtgagcg
agctattttagccgcaaaaaatttagatgttgacgcaatcaattttaaagtacaacaatcattgcctgg
tagtgaaattacattcaaatcgattgacactgtagtgaatcctgatgaagttgtcaattatcctgccga
gtttttaaattcactggatttacctggaatgccaccccataatttgcgattgaagattgattcacctat
aattttgcttagaaatttgaatgctccaaaattgtgtaatggcacgcgactggtcataaaaaaaatcat
gggtaacattttggaagcaactattttaagtggaaaatttaaaggtgaagttgtacttataccgcggat
cccaatgattccttcagattcgcctataccattcaaacgtttgcaatttccaatcctcttagcatttga
tatgactattaataagtcacaaggtcaaacaatgacaatttgtggcttagatttagaaaacccgtgcct
ttctcacggtcaatcatatattgcgtgttccagagttggaaaaccctcgaatttgtttgcgtatgctcc
tgaaggattaaccaaaaatattgtacatctaatggcattacgataacttaagtttttttaattgaaaa
aatatatctgttataaaatagtttaagtttcactttttacctttaattaaaataagcttaagttttg
atttaaaacaacctctacatttaacttcgctgatagtatacacatactcacacgctatcgattggtcga
attgtgataagatatatgtaatgacgttagataatataataactttttaataaaaatttgcataaaaac
gatccagattgttacagcttattcaaaaaaatttgaaattttccaattaaagacgtgtgtacaggacat
cgtctgtcgggtccgctag

>*Papilio machaon* (5841 bp)

tttgaaaaaaatttttgttattcaattcatgtgctgattaaaatattaaatgtaattatttttaaagag
ttttgaagtgtgtataaataattttgtgaattcagatgttttttaattaattttatttgtaaggagt
ttttgattttggaatgttttacatcagattcgacggatggcgctatcatcgcattcaatattattcaat

ttcaatttagtttgtcccgacagatggtgctacgattaatttgaaaaaaatttgttattcaattcat
gtgctgattaaaatattaaatgtaattatttttaaagagttttgaagtgtgtataaataattttgtga
attcagatgtttttttaattgattttttatttgtaaggagtttttgattttggaatgttttacatcagat
tcgatggatggcgctatcatcgcattcaatattattctattttaattttagtttgtcccgacagatagt
gctacgatcaatttgaaaaagtatttcgttattttgtgttataattgtgttattcaagtgaagttgcta
attaaaatatttaatgtaattagttttcgaagagttctgaagtgtatataaataacttatacattttgt
aaatttagtgcggaatcgaagtgagtataaccttcaagtttatttgtgtagttagaggattgaatttt
ttaattattttattgacaattaattcattttggagatattaaataaaaaaactcacaaaatgcgatt
acatgttctttatctaaaatttgtcataataaatcttttgagttaatactcataattatatttatttac
aggtgaagaactgaccaagaaagttagtgccatgaatttctacgcatatcggttaatgattcatgctaa
tgaagataataatattctccgctgtagacagctatttcatcagtgtaattaaaaaaaaataatttagaa
ataaaatctgtcataaaaaattcattatcaataagtaactcatattcattgtatattattttgcaatat
tttatttttacagtagaagaacattataactcatattcattgtatattattttgcaatattttattttt
gtagaaaaatgccacgtcttcgtggaagagcaagaaatattggtcgacgtactcagcacgcgcaattag
ttcatgatcatcgattgaatagaacagtagaagaacattctatggataatgtaaatttaagagatcgag
ctgcatgtacacgtgcaaatgaaaatttagagcaacgtgctcaacgtcttcgtgctaatacattaagac
aacgagaggcacgtcaacgagcgaccaacgcacatagagaacgtaaccaacagcgagtacaagataatc
gagcattggcacgagcatcacttaatcgtctcgcgtttgaatatgatcctgaaatagattattcatcac
atgcattgatcacgattggtagtatggacaaagagtgtcaacattgtcatgctttcaagtacaaaggtg
aatcagctggtttatgttgcgcatctggaaaaatatcactgccaccgctaaaaccaccaccagaacctt
taaaaacgcttttagctggaatcacatctcaatcgaaattatttttacggaaaattcgtaaattaaatt
catgcttccaaatgacatcatttgcagcaacaaaaattattcataatgaagatggccgtaattttgaat
ccacattcaaaattcaaggccaagtatatcaccaaattggttcgttacttccaatgcctgatgccgatc
caaaatttttacaaatttattttatggggaatgaagagcaacaatcacatacacgctgcgtttacaacc
atatagaacagatggaggaacgagaaattgtggacattttggaaaggtttttgcaaaaccataaccaat
tagtacaattgttcaagactctttctaacagactgcaaaacgataactatgtcattgttattaaagcag
acaaagtaccctatggagagcacgcaggcacatataatgttccaaccattaatgaagtggcagttgtta
tggctggtgacccatgtgaacgtagagacattcgcatacaacgcagagataatacgatgcaaataattc
aagacaatcatcgttcttacgatgctttgcagtatccgttaatattttgggaaggagaagacggatatc
atttaaatattaaacaacggaatccaactacaggtgaagaactgaccaagaaagttagtgccatgaatt
tctacgcatatcggttaatgattcgtgctaatgaagataataatattctccgctgtagacagctatttc
atcagtacatcgtcgatatgtatgtaaaaattgaaagtgaaagattaaggtatataaaattcaatcaag
caaaacttcgagctgaagagtatattcatttgcgtgatgcagtaatcggtaatgtagatgcaacaaacg
tcattaacaacatcggtaccacatatattctcccatcatcatatattggtagtccgcgtcatatgcaag
aatatattcaagacgctatgacatacgtacgtgcatacggtcgaccagatctttttatcacatttacat
gtaatccaaattgggatgaaatcaaaaacttgctgttgtcaggtcaaacatcgatgcatcgccacgata
tcactgcgcgtgtctttaaacaaaaattaaaatctttgatgaatttgatcacacattactcggttttg
gtgaaacacgttgttggctttactctgttgagtggcaaaaacgaggtttacctcatgcgcatattttga
tttggttggtggataaagtacgcccagaagaaattgacaaaattatttcagcggaaattccagatccga
atgttgatcaagaactgtttaacattgttactactaatatgattcatggtccatgcggtactctaaaca
tgatgtcaccatgcatgaatgatggaaaatgtacgaaacgttttccaaaaccatgtcaaactgatacta
tcaccaatattgacggttatccatcttaccggcgcagagacgtagataatggcggtcaatcgtatgaat
tgcgactgtcaaacggtgtaagagtagatattgataatcggtgggtggttccatattcgccattactat
gcaaaacctataaagcgcacataaacgttgaactgtgcagttcggtgaaatctatcaaatacatttgta

agtacgttcacaagggcagtgataaagctatatttgctgttcaaaatgtaaatgacaatgacgaaataa
cacgttatcaaatgggtcgatacataagtagcaatgaagctgtctggcgcattttacgtttcctatac
atgaaagggatcctgcagttatacatttggctgtgcatcttgaaaacggacagcgtgtttattttacag
aacagactgcactacaacaagcattaacggctccaaaaacaactcttactgaattttcaatctttgta
atcgacaagatgttgttggtcaattcgcaaaaacattaatgtatactgatgttcctaaatttttacat
ggaataagcaatcgaaaaattgggaaccacgaaaacgagggattccagtcacaggattcgccgacatat
ttatgacaaatactttaggtcgactatatacagttcatcctaagcaacgcgaatgtttttttttgcgtt
tgttattggttaatgttcttggaccgacatccttccaatatttacgaaaagtcaatggtattttatacg
acactttcttcgatgcgtgtcgtgagttacatttattggaggatgataaccattgggatctcacacttg
cagatgcagcactgagctcatctccacaacaaattcgtcaattattttcaataatattaacaacgtgtt
ttccgtctgaagcatctgctctctggaacaaatataaagactcaatgagtgaagatattttgcatcgga
ttagaattactaatcaaaatcctgatattcaattctccgcagaaatatacaatgaggctttaattatga
ttgaggatatttgtattcttatttcaaacatgccactcatccattttggcatgccggcgccgaaccgcc
cagcagtagatatcatcaacagtgatgttcaacgtgaacaccagtttgataagacttcgttggctactt
ttgttgctaataatgaacaattgcttacagctgaacaacgaaatgtatatgatcaaattaacgtatcaa
ttgcagcacaacaaggtggattcttttttttggatgcaccaggtggcactggtaaaacctttctcatct
cactgatacttgcgcgcattcgatcacaaaatcatattgcattggccattgcttcatcaggcattgcag
cgacgttacttgatggtggacggactgcgcattcagcacttaagttacctttgaatgttcatacaaatc
ccgaagcaatgtgtaacataaagaagcattccggcatggctgaagttttgagaaaatgcaaaattatta
tttgggatgaatgtacaatggcccacaagcattcgcttgaagctctcgacaggtccctgaaggatatca
aagataatactaggcttttcggtggtgctctactgctactgtctggtgatttcaggcaaacattgccag
taattccacgtgcgacatatgcagacgaagtaaacgcatgtttgaaggaatcttatctatggcggagtg
tcagtaaattgagccttactatcaatatgcgcgttcaactacaaaatgatccattagcgtcaggattct
ctgaacaattgttagacattggcaacggtaaaattcaactgtatgaagatacccaatttattaaatttc
cagagaacttttgcaacatggtggctaccaaagatgaattaataaatagtatctttccagatttaagat
ataactatactaatcatgaatggctacgagagcgagctattttagctgcaaaaaatttagatgtagacg
ccatcaattttaaaatacaacagtcattgcctggtaatgaaattacatttaaatcaattgacaccgttg
ttgatcctgacgaagtggtcaactatccagtagaatttttgaattctttagatttacctggaatgccac
cacataatttgcgactgaagattggctcacctataattttgcttcgtaatttaaatgccccgagattgt
gtaatggcacgcgattagttataaaaaaaatcatgggcaacattattgaagcgaatattttatctggaa
aattccaaggtgaaattgtacttttaccgcggatcccaatgattccttcagattcacctataccattta
aacgcttacaatttccgatccgtttggcatatgctatgactattaataagtcacaaggtcaaacgatga
cattttgtggcttagatttagaaaatccgtgtttttctcacggccaattgtacgttgcgtgttccagag
ttggaaaaccatcgagtttgtttgtttatactcctcatggactaactaaaaatattgtacatccaatgg
cattacgataaattaagttttgtaaattaaaaataaataaatatatatgttataaaatgttttgagtg
caagcttttcacctttcattaaaatgagttaaagttttcatttacgatgctgttatttatttaacaaa
gtaataacttttcaataataattttcatcaaagcggtccaaattgtttcagctcattaaaaaaagtatg
aaattaaagacgtgtgtacaggacaacgtctgtcgggtccgctag

> *Cotesia vestalis* (5702 bp)

tcttaatatatatatttcttgtgtgcgtgtgtatttgactgaactcctcctaaacgactggaccaattt
tgatgaaatttttgtgtgtgttcgtggagattcgagaatggtttagatttacagtttggtctactgga
aaatgtttttcaattaatttcttatttataaggagttgttgattttggaatgttttacattagatccg
gcggacggcgctatcatcgcattcaatattattctatttcaattttagtttgtcctgacagatggtgct
acgattaatttgaaaaaaatttgttattcaattcatgtgctgattaaaatattaaatgtaattatttt

ttaaagagttttgaagtgtgtataaataatttgtgaattcagatgtttttttaattaattttatttg
taaggagtttttgattttggaatgttttacatcagattcgacggatggcgctatcatcgcattcaatat
tattctattttaattttagtttgtcccgacagatagtgctacgatcaatttgaaaaagtatttcgttat
tttgtgttataattgtgttattcaagtgaagttgctaattaaaatatttaatgtaattagttttcgaag
agttctgaagtgtatataaataacttatacattttgtaaatttagtgcgtaatcgaagtgagtatattt
tttataaattatctttttttaatttacacgtgcgtttattaacaaccaacataaccttcaagtttatttg
tgtagttagaggattgaatttttttaattatttttattgacaattaattcatttttggagatattaaat
aaaaaaactcacaaaatgcgattacatgttctttatctaaaatttgtcataataaatcttttgagttaa
ttaattaaaaattcattatcaataagtaactcatattcattgtatattattttgcaatattttattttt
gtagaaacatgccacgtcttcgtggaagagcaagaaatattggtcgacgtactcagcacgcgcaattag
ttcatgatcatctattgaatagaacagtagaagaacattctatggataatgtaaatttaagagatcgag
ctgcatgtacacgtgcaaatgaaaatttagagcaacgtgctcaacttcttcgtgctaatacattaagac
aacgagaggcacgtcaacgagcgaccaacgcacatagagaacgtaaccaacagcgagtacaagataatc
gagcattggcacgagcattacttaatcgtctcgcgtttgaatatgatcctggaatagattattcatcac
atgcatttatcacgattggtagtatggacaaagagtgtcaacattgtcatgctttcaagtacaaaggtg
aatcagctggtttatgttgcgcatctagaaaaatatcactgtcaccgctaaaaccaccaccagaacctt
taaaaacgcttttagctggaatcacatctcaatcgaaattatttttacggaaaattcgtaaattaaatt
catgcttccaaatgacatcatttgcagcaacaaaaattattcataatggagatggccgtaattttgaat
ccacattcaaaattcaaggccaagtatatcaccaaattggttcgttacttccaatgcctgatgccgatc
caaaatttttacaaatttattttatggggaatgaagagcaacaatcacatacacgctgcgtttacaacc
atatagaacagatggaggaacgagaaattgtggacattttggaaaggtttttgcaaaaccataaccaat
tagtacaattgttcaagactctttctaacagactgcaaaacgataactatgtcattgttattaaagcag
acaaagtaccctatggagagcacgcaggcacatataatgttccaaccattaatgaagtggcagttgtta
tggctggtgacccatgtgaacgtagagacattcgcatacaacgcagagataatacgatgcaaataattc
aagacaatcatcgttcttacgatgctttgcagtatccgttaatattttgggaagcagaagacggatatc
atttaaatattaaacaacggaatccaactacaggtgaagaactgaccaagaaagttagtgccatgaatt
tctacgcatatcggttaatgattcgtgctaatgaagataataatattctccgctgtagacagctatttc
atcagtacatcgtcgatatgtatgtaaaaattgaaagtgaaagattaaggtatataaaattcaatcaag
caaaacttcgagctgaagagtatattcatttgcgtgatgcagtaatcggtaacgtagatgcaacaaacg
tcattaacaacatcggtaccacatatattctcccatcatcatatattggtagtccgcgtcatatgcaag
aatatattcaagacgctatgacatacgtacgtgcatacggtcgaccagatctttttatcacatttacat
gtaatccaaattgggatgaaatcaaaaacttgctgttatcaggtcaaacatcgatgcatcgccacgata
tcactgcgcgtgtctttaaacaaaaattaaaatctttgatgaatttgatcacacattactcggtttttt
gtgaaacacgttgttggctttactctgttgagtggcaaaaacgaggtttacctcatgcgcatattttga
tttggttggtggataaagtacgcccagaagaaattgacaaaattatttcagcggaaattccagatccga
atgttgatcaagaactgtttaacattgttactactaatatgattcatggtccatgcggtactctaaaca
tgatgtcaccatgcatgaataatggaaaatgtaagaaacgttttccaaaaccatgtcaaactgatacta
tcaccaatattgacggttatccatcttaccggcgcagagacgtagataatggcggtcaatcgtatgaat
tgcgactgtcaaacggtgtaagagtagatattgataatcggtgggtggttccatattcgccattactat
gcaaaacctataaagcgcacataaacgttgaactgtgcagttcggtgaaatctatcaaatacatttgta
agtacgttcacaagggcagtgatgaagctatatttgctgttcaaaatgtaaatgacaatgacgaaataa
cacgttatcaaatgggtcgatacataagtagcaatgaagctgtctggcgcatttttacgtttcctttac
atgaaagggatcctgcagttatacatttggctgtgcatcttgaaaacggacagcgtgtttattttacag
aacagactgcactacaacaagcattaacggctccaaaaacaactcttactgaatttttcaatcttggta

atcgacaagatgttgttggtcaattcgcaaaaacattaatgtatactgatgttcctaaatttttacat
ggaatgaacaatcgaagaattgggaaccgcggaaacgaggcattccaatcccaggattcgctgatatat
ttatgacaaatactttaggtcgattatatacagttcatcctaagcaacgcaaatgtttttttttgcgtt
tattactggttaacgtccctggaccgacgtccttccaatatttgcgaaaagtcaacggtactttatacg
actctttcttcgatgcgtgtcgtgagttacatttattggatgatgataaccattgggatctcacacttg
cagatgcagcactgagctcttctccacaacaaattcgtcaattattttcaataatattaacaacgtgtt
ttccgtctgaagcatctgctctctggaacaaatataaagactcaatgagtgaagatattttgcatcgga
ttagaattactaatcaaaatcctgatattcaattctccgcagaaatatacaatgaggctttaattatga
ttgaggatatttgtattcttatttcaaacatgccactcatccattttggcatgccggcgccgaaccgcc
cagcagtagatatcatcaacagtgatgttcaacgtgaacaccagtttgataagacttctttggctactt
ttgttgctaataatgaacaattgcttacagctgaacaacgaaatgtatatgatcaaattaacgtatcaa
ttgtaccacaacaaggtggattcttttttttggatgcaccaggtggcactggtaaaacatttcttatcg
cactgatacttgcgcggattcgatctcaaaatcatatggcattggccattgcttcatcaggcattgcag
caacgttacttgatggtggacggactgcgcattcagcacttaaattacctttgaacgttcatacaaatc
ccgaagcaatgtgtaacatgcacaagcattcaggcatggctgaagttttgagaaaatgtaaaattatta
tctgggatgaatgtacaatggcccacaagcatccgcttgaagctctcgacaggtccctgaaagatatca
aaaataatgctcggcttttcggtggtgctctactgctgctgtctggtgatttcagacaaacattaccag
tcattccacgcgcgacatatgcagacgaaataaacgcatgtttgaaagaatcttatctatggccaagtg
tcactaaattatgccttactattaatatgcgcgttcgacttcaaaatgatccattagcgtcaggattct
ctgaaaaattgttagacattggcaacggtaaaattcaattgtatgaaaatacacaatatattcgacttc
cagagaattttcgcaacatggtggctaccaaagatgagttaataacaagtatctttccagatttaagac
ataattatactaatcatgaatggctgcgagagcgagctattttagccgcaacaaatttagatgttgacg
ccatcaattttaaaatacaacagtcattgcctggtgatgaaattacatttaaatcgattgacactgttg
ttgatcctgacgaagttgtcaactattctgaagagtttctaaattcattagatttacctggaatgccac
cacataatttgcgactgaaaactggctcacctataattttgcttcgaaatttaaatgcatcaaaattgt
gtaatggcacgcgattagttgtaaaaaaaatcatgggcaacattcttgaagccactattttgggtggaa
aatttcaaggtgaagttgtacttttaccacggatcccaatgattccttcagattcgcctataccattca
aacgtttccaatttccaatccgcttggcatatgctatgactataaataagtcacaaggccaaacaatga
caatttgtggcttagatttagaaaacccatgtttttctcacggccaattatatgttgcttgttccagag
ttggaaaaccatcgaatttatttatttatacacctcagggtttaaccaaaaatattgtacatccaatgg
cattacgataaattaagtttttgtaaattaaaaataaatatatatatatgttataaaatgttttgagtg
caagctttttcacctttcattaaaatgagttaaagttttcatttacgatgctgttatttatttaacaaa
gtaataacttttcaataataattttcatcaaagcggtccagattgttcagctcattaaaaaaagtatga
aattaaagacgtgtgtacaggacaacgtctgtcgggtccgctag

>*Athalia rosae* (5454 bp)

tcttaatatatatatttcttgagtgcgtgtgtatgtgactgaactcctcctaaacgactggaccaattt
tgatgaaatttttttgtgtgtgttcgtggagattcgagaatggtttagatttacagtttggtctactgga
aaatgtttttttcaattaatttgttatttataaggagttgttgattttggaatgttttacattagatccg
gtggacggcgctatcatcgcattcaatattattctatttcaattttagtttgtcccgacagatagtgct
acgataaatttgaaaaagtatttcgttattttgaattaaaattgtgttattcaagtgaagttgctaatt
aaaatatttaatgtaattagttttcgaagagttgtgaagtgtatataaataacttatacattttgtaaa
tttagtgcggaatcgaagtgagtatattttgtataaattatctttttttaatttacacgtgcgtttatta
acaaccaacataaccttcaagtttatttgtgtagttagtgaatttttttaattatttttattgacaatt
aattcattttttggagataataaaaaaaaaaaaaatcacaaaatgcgattacatgttctttatctaaaattt

gtcataataaatctttagagttaattaattaaaaatacattatcaataagtaactcatattcattgtat
attattttgcaatattttatttttgtagaaaaatgccacgtcttcgtggaagagcaagaaatattggtc
gacgtactcagcacgcgcaattagtacatgatcatagattgaatagaacagtagaagaacattctatgg
ataatgtaaatttaagagatcgagctgcatgtacacgtgcaaatgaaaatttagagcaacgtgctcaac
gtcttcgtgctaatacattaagacaacgagaggcacgtcaacgagcgaccaacgcagatagagaacgta
accaacagcgagtacaagataatcaagcattggcacgagcatcacttaatcgtcgcgcgtgtgaatatg
atccagaaatagattattcatcacatgcattgatcacgattggtagtatggacaaagagtgtcaacatt
gtcatgcttacaagtacaaaggtgaatcagctggtttatgttgcgcatctggaaaaatatcactgccac
cgctaaaaccaccaccagaacctttaaaaacgcttttagcaggaatcacatctcaatcgaaattatttt
taaggaaaattcgtaaattaaattcatgcttccaaatgctctcatctgacacaacaaaaattattcata
atgaagatggccgtaattttgaatccacattcaaaattcaaggccaagtatatcaccaaattggttcgt
tacttccaatgcctgatgccgatccaaaatttttgcaaatttatttcatggggaatgaagagcaacaat
cacataaacgctgcgtttacaaccatatagaacagatggaggaacgagaaattgtggacattttgaaaa
ggtttttgaaaaaccataaccaattagtacaattgttcaagactctttctaacagactgcaaaacgata
actatgtcattgttattaaagcagacaaagtaccctatggagagcacgcaggcacatataatgttccaa
ccattaatgaagtggcagttgttatggctggtgacccatgtgaacgtagagacattcgcatacaacgca
gagataatacgatgcaaataattcaagacaatcatcgttcttacgatgctttgcagtatccgttaatat
tttgggaaggagaagacggatatcatttaaatattaaacaacggaatccaactacaggtgaagaactga
ccaagaaagttagtgccatgaatttctacgcatatcggttaatgattcgtgcaaatgaagataataata
ttctccgctgtagacagctatttcatcagtacatcgtcgatatgtatgtaaaaattgaaagtgaaagat
taaggtatataaaattcaatcaagcaaaacttcgagctgaagagtatattcatttgcgggatgcagtaa
tcggtaatgtagatgcaacaaacgtcattaacaacatcggtaccacatatattctcccatcatcatata
ttggtagtccgcgtcatatgcaagaatatattcaagacgctatgacatacgtacgtgcatacggtcgac
cagatctttttatcacatttacatgtaatccaaattgggatgaaatcaaaaacttgctgttgtcaggtc
aaacatcgatgcatcgccacgatatcactgcgcgtgtctttaaacaaaaattaaaatctttgatgaatt
tgatcacacattactcggtttttggtgaaacacgttgttggctttactctgttgagtggcaaaaacgag
gtttacctcatgcgcatattttgatttggttggtggataaagtacgcccagaagaaattgacaaaatta
tttcagcggaaattccagatccgaatgttgatcaagaactgtttaacattgttactactaatatgattc
atggtccatgcggtactctaaacatgatgtcaccatgcatgaatgatggaaaatgtacgaaacgttttc
caaaaccatgtcaaactgatactatcaccaatattgacggttatccatcttaccggcgcagagacgtag
ataatggcggtcaatcgtatgaattgcgactgtcaaacggtgtaagagtagatattgataatcggtggg
tggttccatattcgccattactatgcaaaacctataaagcgcacataaacgttgaactgtgcagttcgg
tgaaatctatcaaatacatttgtaagtacgttcacaagggcagtgataaagctatatttgctgttcaaa
atgtaaatgacaatgacgaaataacacgttatcaaatgggtcgatacataagtagcaatgaagctgtct
ggcgcattttttacgtttcctatacatgaaagggatcctgcagttatacatttggctgtgcatcttgaaa
acggacagcgtgtttattttccagaacagactgcactacaacaagcattaacggctccaaaaacaactc
ttactgaattttttcaatctttgtaatcgacaagatgttgttggtcaattcgcaaaaacattaatgtata
ctgatgttcctaaatttttttacatggaataagcaatcgaaaaattgggaaccacgaaaacgagggattc
cagtcccaggattcgccgacatatttatgacaaatactttaggtcgactatatacagttcatcctaagc
aacgcgaatgtttttttttgcgtttgttattggttaatgttcttggaccgacatcctttcaatatttac
gaaaagtcaatggtattttatacgacactttcttcgatgcgtgtcgtgagttacatttattggaggatg
ataaccattgggatctcacacttgcagatgcagcactgagctcttctccacaacaaattcgtcaattat
tttcaataatattaacaacgtgttttccgtctgaagcatctgctctatggaacaaatataaagactcaa
tgagtgaagatattttgcatcggatcagaattactaatcaaaatcctgatagtcaattctccgcagaaa

tatacaatgaggctttaataatgattgaggatatttgtattcttatttcaaacatgccactcatccatt
ttggcatgccggcgccgaaccgcccagcagtagatatcatcgacagtgatgttcaacgtgaacaccagt
ttgataagacttcgttggctactttgttgctaataatgaacaattgcttacagctgaacaacgaaatg
tatatgatcgaattaacgtatcaattgcagcacaaaaaggtggattctttttttggatgcaccaggtg
gcactggtaaaacctttctcatctcactgatacttgcgcgcattcgatcacaaaatcatattgcattgg
ccattgcttcatcaggcattgcagcgacgttacttgatggtggacggactgcgcattcagcacttaagt
tacctctattgaatgttcatacaaatcccgaagcaatgtgtaacataaagaagcattccggcatggctg
aagttttgagaaaatgcaaaattattatttgggatgaatgtacaatggcccacaagcattcgcttgaag
ctctcgacaggtccctgaaggatatcaaagataatactaggcttttcggtggtgctctactgctactgt
ctggtgatttcaggcaaacattgccagtaattccacgtgcgacatatgcagacgaagtaaacgcatgtt
tgaaggaatcttatctatggcggagtgtcagtaaattgagccttactatcaatatgcgcgttcaactac
aaaatgatccattagcgtcaggattctctgaacaattgttagacattggcaacggtaaaattcaactgt
atgaaaatacccaatttattaaatttccagagaacttttgcaacatggtggctaccaaagatgaattaa
taaatagtatctttccagatttaagatataactatactcatcatgaatggctacgagagcgagctattt
tagctgcaaaaaatttagatgtagacgccatcaattttaaaatacaacagtcattgcctggtaatgaaa
ttacatttaaatcaattgacaccgttgttaatcctgacgaagtggtcaactatccagtagaatttttga
attctttagatttacctggaatgccaccacataatttgcgactgaagattggctcacctataattttgc
ttcgtaatttaaatgccccgagattgtgtaatggcacgcgattagttataaaaaaaatcatgggcaaca
ttattgaagcgaatattttatctggaaaattccaaggtgaaattgtacttttaccgcggatcccaatga
ttccttcagattcacctataacatttaaacgcttacaatttccgatccgtttggcatatgctatgacta
ttaataagtcacaaggtcaaacgatgacattttgtggcttagatttagaaaatccgtgtttttctcacg
gccaattgtacgttgcgtgttccagagttggaaaaccatcgagtttgtttgtttatacttctcatggac
taactaaaaatattgtacatccaatggcattacgctaaattaagtttttgtaaattaaaaataaataaa
tatatatgttataaaatgttttgagtgcaagctttttcacctttcattaaaatgagttaaagttttcat
ttacgatgctgttatttatttaacaaagtaataacttatcaataataattttcatcaaagcggtccaga
ttgtttcagctcattaaaaaaagtatgaaattaaagacgtgtgtacaggacaacgtctgtcgggtccgc
tag
