# Supplementary material for: Characterization of a novel Helitron family in insect genomes: insights into classification, evolution and horizontal transfer
Source: Mob DNA. 2019 May 31;10:25. doi: 10.1186/s13100-019-0165-4 (PMC6544945; doi:10.1186/s13100-019-0165-4)
Supplement: Supplementary file 5 — Data S4. The consensus sequences of 35 Helitron exemplars. Data S5. The sequences used for evolutionary analysis. (PDF 101 kb) [file 13100_2019_165_MOESM5_ESM.pdf]

Data S4. The consensus sequences of 35 *Helitron* exemplars.

>Agla_Hel1Aa

TCTTTATATATATAATTCTTCTGTGCGTGTGTATGTCACTGAACTCCTCCTAAACGGCTGGACCGATTT
TGATGAAATTTTTTGTGTGTGTTCAAGTGGATTCGAGAATGGTTTAGATTCACAATTCGATTCGATAGA
TATGTTTTTTTAATTAATTTAACGTTTGAACAGGACAACGTTTGTCGGGTTCGCTAG

>Aros_Hel1Aa

TCTTAATATATATATTTCTTGAGTGCGTGTGTATGTGACTGAACTCCTCCTAAACGACTGGACCAATTT
TGATGAAATTTTTTGTGTGTGTTCGTGGAGATTCGAGAATGGTTTAGATTTACAGTTTGGTCTACTGGA
AAATGTTTTTTCAATTAATTTGTTATTTATAAGGAGTTGTTGATTTTGGAATGTTTTACATTAGATCCG
GTGGACGGCGCTATCATCGCATTCAATATTATTCTATTTCAATTTTAGTTTGTCCCGACAGATAGTGCT
ACGATAAATTTGAAAAAGTATTTCGTTATTTTGAATTAAAATTGTGTTATTCAAGTGAAGTTGCTAATT
AAAATATTTAATGTAATTAGTTTTCGAAGAGTTGTGAAGTGTATATAAATAACTTATACATTTTGTAAA
TTTAGTGCGGAATCGAAGTGAGTATATTTTGTATAAATTATCTTTTTTAATTTACACGTGCGTTTATTA
ACAACCAACATAACCTTCAAGTTTATTTGTGTAGTTAGTGAATTTTTTTAATTATTTTTATTGACAATT
AATTCATTTTTGGAGATAATAAAAAAAAAAAATCACAAAATGCGATTACATGTTCTTTATCTAAAATTT
GTCATAATAAATCTTTAGAGTTAATTAATTAAAAATACATTATCAATAAGTAACTCATATTCATTGTAT
ATTATTTTGCAATATTTTATTTTTGTAGAAAAATGCCACGTCTTCGTGGAAGAGCAAGAAATATTGGTC
GACGTACTCAGCACGCGCAATTAGTACATGATCATAGATTGAATAGAACAGTAGAAGAACATTCTATGG
ATAATGTAAATTTAAGAGATCGAGCTGCATGTACACGTGCAAATGAAAATTTAGAGCAACGTGCTCAAC
GTCTTCGTGCTAATACATTAAGACAACGAGAGGCACGTCAACGAGCGACCAACGCAGATAGAGAACGTA
ACCAACAGCGAGTACAAGATAATCAAGCATTGGCACGAGCATCACTTAATCGTCGCGCGTGTGAATATG
ATCCAGAAATAGATTATTCATCACATGCATTGATCACGATTGGTAGTATGGACAAAGAGTGTCAACATT
GTCATGCTTACAAGTACAAAGGTGAATCAGCTGGTTTATGTTGCGCATCTGGAAAAATATCACTGCCAC
CGCTAAAACCACCACCAGAACCTTTAAAAACGCTTTTAGCAGGAATCACATCTCAATCGAAATTATTTT
TAAGGAAAATTCGTAAATTAAATTCATGCTTCCAAATGCTCTGACATCTGACACATGACAAAAATTATT
CATAATGAAGATGGCCGTAATTTTGAATCCACATTCAAAATTCAAGGCCAAGTATATCACCAAATTGGT
TCGTTACTTCCAATGCCTGATGCCGATCCAAAATTTTTGCAAATTTATTTCATGGGGAATGAAGAGCAA
CAATCACATAAACGCTGCGTTTACAACCATATAGAACAGATGGAGGAACGAGAAATTGTGGACATTTTG
AAAAGGTTTTTGAAAAACCATAACCAATTAGTACAATTGTTCAAGACTCTTTCTAACAGACTGCAAAAC
GATAACTATGTCATTGTTATTAAAGCAGACAAAGTACCCTATGGAGAGCACGCAGGCACATATAATGTT
CCAACCATTAATGAAGTGGCAGTTGTTATGGCTGGTGACCCATGTGAACGTAGAGACATTCGCATACAA
CGCAGAGATAATACGATGCAAATAATTCAAGACAATCATCGTTCTTACGATGCTTTGCAGTATCCGTTA
ATATTTTGGGAAGGAGAAGACGGATATCATTTAAATATTAAACAACGGAATCCAACTACAGGTACAACT
TATAGATTATCTTAAAAATTTTTTTAGGAATAAATAATTTTTGTTTATCTCATAATTATATTTATTGAC
AGGTGAAGAACTGACCAAGAAAGTTAGTGCCATGAATTTCTACGCATATCGGTTAATGATTCGTGCAAA
TGAAGATAATAATATTCTCCGCTGTAGACAGCTATTTCATCAGTACATCGTCGATATGTATGTAAAAAT
TGAAAGTGAAAGATTAAGGTATATAAAATTCAATCAAGCAAAACTTCGAGCTGAAGAGTATATTCATTT
GCGGGATGCAGTAATCGGTAATGTAGATGCAACAAACGTCATTAACAACATCGGTACCACATATATTCT
CCCATCATCATATATTGGTAGTCCGCGTCATATGCAAGAATATATTCAAGACGCTATGACATACGTACG
TGCATACGGTCGACCAGATCTTTTTATCACATTTACATGTAATCCAAATTGGGATGAAATCAAAAACTT
GCTGTTGTCAGGTCAAACATCGATGCATCGCCACGATATCACTGCGCGTGTCTTTAAACAAAAATTAAA
ATCTTTGATGAATTTGATCACACATTACTCGGTTTTTGGTGAAACACGTTGTTGGCTTTACTCTGTTGA
GTGGCAAAAACGAGGTTTACCTCATGCGCATATTTTGATTTGGTTGGTGGATAAAGTACGCCCAGAAGA
AATTGACAAAATTATTTCAGCGGAAATTCCAGATCCGAATGTTGATCAAGAACTGTTTAACATTGTTAC

TACTAATATGATTCATGGTCCATGCGGTACTCTAAACATGATGTCACCATGCATGAATGATGGAAAATG
TACGAAACGTTTTCCAAAACCATGTCAAACTGATACTATCACCAATATTGACGGTTATCCATCTTACCG
GCGCAGAGACGTAGATAATGGCGGTCAATCGTATGAATTGCGACTGTCAAACGGTGTAAGAGTAGATAT
TGATAATCGGTGGGTGGTTCCATATTCGCCATTACTATGCAAAACCTATAAAGCGCACATAAACGTTGA
ACTGTGCAGTTCGGTGAAATCTATCAAATACATTTGTAAGTACGTTCACAAGGGCAGTGATAAAGCTAT
ATTTGCTGTTCAAAATGTAAATGACAATGACGAAATAACACGTTATCAAATGGGTCGATACATAAGTAG
CAATGAAGCTGTCTGGCGCATTTTTACGTTTCCTATACATGAAAGGGATCCTGCAGTTATACATTTGGC
TGTGCATCTTGAAAACGGACAGCGTGTTTATTTTCCAGAACAGACTGCACTACAACAAGCATTAACGGC
TCCAAAAACAACTCTTACTGAATTTTTCAATCTTTGTAATCGACAAGATGTTGTTGGTTAATTCGCAAA
AACATTAATGTATACTGATGTTCCTAAATTTTTTACATGGAATAAGCAATCGAAAAATTGGGAACCACG
AAAACGAGGGATTCCAGTCCCAGGATTCGCCGACATATTTATGACAAATACTTTAGGTCGACTATATAC
AGTTCATCCTAAGCAACGCGAATGTTTTTTTTTGCGTTTGTTATTGGTTAATGTTCTTGGACCGACATC
CTTTTAATATTTACGAAAAGTCAATGGTATTTTATACGACACTTTCTTCGATGCGTGTCGTGAGTTACA
TTTATTGGAGGATGATAACCATTGGGATCTCACACTTGCAGATGCAGCACTGAGCTCTTCTCCACAACA
AATTCGTCAATTATTTTCAATAATATTAACAACGTGTTTTCCGTCTGAAGCATCTGCTCTATGGAACAA
ATATAAAGACTCAATGAGTGAAGATATTTTGCATCGGATCAGAATTACTAATCAAAATCCTGATAGTCA
ATTCTCCGCAGAAATATACAATGAGGCTTTAATAATGATTGAGGATATTTGTATTCTTATTTCAAACAT
GCCACTCATCCATTTTGGCATGCCGGCGCCGAACCGCCCAGCAGTAGATATCATCGACAGTGATGTTCA
ACGTGAACACCAGTTTGATAAGACTTCGTTGGCTACTTTTGTTGCTAATAATGAACAATTGCTTACAGC
TGAACAACGAAATGTATATGATCGAATTAACGTATCAATTGCAGCACAAAAAGGTGGATTCTTTTTTTT
GGATGCACCAGGTGGCACTGGTAAAACCTTTCTCATCTCACTGATACTTGCGCGCATTCGATCACAAAA
TCATATTGCATTGGCCATTGCTTCATCAGGCATTGCAGCGACGTTACTTGATGGTGGACGGACTGCGCA
TTCAGCACTTAAGTTACCTTTCTACAACACATGCATTAAAAACTCGTGTTTTTTGCCCTCCTCGGCGGG
ATGGAAGTCACAGTTTTCGGACGAGCGGGCAAAAAACTTGCCAGCGGGCAAAAAACTCTGGCAGCAGGG
AAATAAACTCGCCGCCGGGAAAAAAACTCTGTCCGCCGGGAAAAAAACTCTCGTAGCCGTGTAATAAAC
CAAGAAAGTGCTGCCCCTAGCGTAGAACTATATTGAGTACGATGTTTTCATGACGAGTTGTATACTAAC
CGAAGGACTAAGGCGCCGTTCGAACGGTTTTAAGTGTTCTAACCTCAAAAATGGAGTCTACTACAGAAA
ATGACCGTCCAACTACGCCTCCTGATATACGTGACGCAGCGGATAAAGTTTCATTTCACCTGTTGCCGG
ATAAATCGAAAGAAGTTTATGAATCCGCCTACAAGGCATACACCGACTGGAGAACGTCCAAAGGCGCGA
ATTCGTCTTCGGAATCAGTTTTACTGGCATATTTCGATCAGATGTCGCAACAATATAAACCTACAACTA
TCTGGTCGTTATACTCGAAGCTCAAAAGCACCATCAATATCAGAGAAAAAGTTGACATAAGCAGCTACA
AAACATTATCGGCTTTTTTAAAAAAACAGTCACGTGGATTTCGCAGCAAAAAATCCAGCGTATTTACAT
CCGAGGAAGTACAGTCTTTTTTGACAAGGGCCCCAGACGACAAATATCTTGCAACAAAGGTAAAATTAC
AGTATATACATATAACATACACTCTACACACCTACACCTACACTCTATTTACAATCAACTGTGTTATTT
ACAGATCGTTCTAATTTTTGGGATTTTCGGCGCATTGAGGACCGATGAATTAACAAACATTCAAGTACG
CGATATGAAAAAGCAAGGGAATCTACTGCAGGTCCAAGTTCCTAAAACTAAAACGATGCTACCACGATC
TTTCATAATTCCTGAGGATTGTGTGGAGTACGTTACACGATATCAGTCTCTCCGTCCTGAAAATGTACC
CTCCGGCCGCTTCTTCATAAATTACCAAAATGGTAAATGTACTGCGCAGGTGATTGGAAAAAACAAGTT
TGCCGGTATGCCGAAAGAAATCGCTGAGTTTCTTGATTTACCTGATGCTGCATCATACACCGGCCATAC
TTTTCGCCGTACCTCCGCAACAATGCTTGTTGACAATGGAGCGAACATGGAAACGCTGAAGAGGCATGG
TGGCTGGAAATCAAGCACTGTTGCGGAGGGATACATAGCAGAATCCTTAGGAAACAAAAAAAATATCGG
GGATATGATAATGTCTTCTCTGAATTTGCCTTCAACAGCAACCTCAGCCGACAACCGCAGTGTCAAAAT
ATCATCGCCAAAGCGTCCTAAAATGGACGATTCAATAAACGCAGTACCTTCTACAGCGAAAGTGCATTG
TGGCCCGGAGCAAAAAGTATTCACTTTCGAAAATTGTACAAATGTTACAATTAATTACACAGGGAATCC

GGGAAATCCTCTGTAATTTTCTATTAGCTCATTAGCTGTGATAAAACGTAACAAAACCACTCTTGGCTC
GAGAGTAAGGTTGTCAGAGGTTTTTAGATGATTGGATGATATTAATAATAAGAAAAACTGATTTGTGAT
GATAAACATATCCTGAATATAAAATATATAAGTCTAAATATACGTACCTACTATTAGAGATAAGAAACT
GATTTCAAGTTAAGCTTGATTGCAGTACCTATGCATACTATTAAAATTAGGGAAAATTCTGTGATTCCA
AGAGAATAAGAGTGAACATAGTTTAAGTTTGATACTATCATAAATATAAAAAGAACTTTGTGATTTCAA
CATCAATAAAAATCATCTTGAAAATATAAGAAACAAAAATATGAAAATATTTAAAATATAAAATATATA
ATCCTGATATAATTGAATATGAAAAAACAGATGTAAGGTTTTCATTTTGCTAGAAAGATTTTTATGAAA
CATCAGATCTTTGATAATGTTTTTAGTCGGGGGGCTTTGCCCCCCGGCCCCCCACGGGGGCTTCGCCCC
TCGACCCCACCAGGGGCTCCGCCCCTGGACCCCGCCGTCACGCACAGTGGTCTATCACAGTTCAGTTTT
TCAAGGATGTAGTAACTATTTGATCGCATTTCTGCAAAGTCCGAATTATGCACACCTTCGGCGGTTTTT
TAGGTCATTTTATCGTAGTCGGGTAAGAAGTAGTATATGCCACACGGGAGGAGCGGATTTTCACCTTGT
GCTCGTGCAAACCTCGCTTTGTCGTAACTTAACCTGCCAATGCATGAGTAATTCGTAATTTCCAATATG
TATTTTTCACTCGTTTTTAATGCATGTGTTATAGAAAATAGTATATGCACCACGGGAAAGGGCAAGCGT
TTTGTCTCGCGCGATGTTTGCCGAACTCGTCTTCACTCTCGGGCGCCTTCGGCGCCCTCGACTTACAAC
TCGTTCTGCAAACATCGGCGCTCGACCGAAAACACATGCTTCCTTCCCTAGATGCATAATATACTATTG
AATGTTCATACAAATCCCGAAGCAATGTGTAACATAAAGAAGCATTCCGGCATGGCTGAAGTTTTGAGA
AAATGCAAAATTATTATTTGGGATGAATGTACAATGGCCCACAAGCATTCGCTTGAAGCTCTCGACAGG
TCCCTGAAGGATATCAAAGATAATACTAGGCTTTTCGGTGGTGCTCTACTGCTACTGTCTGGTGATTTC
AGGCAAACATTGCCAGTAATTCCACGTGCGACATATGCAGACGAAGTAAACGCATGTTTGAAGGAATCT
TATCTATGGCGGAGTGTCAGTAAATTGAGCCTTACTATCAATATGCGCGTTCAACTACAAAATGATCCA
TTAGCGTCAGGATTCTCTGAACAATTGTTAGACATTGGCAACGGTAAAATTCAACTGTATGAAAATACC
CAATTTATTAAATTTCCAGAGAACTTTTGCAACATGGTGGCTACCAAAGATGAATTAATAAATAGTATC
TTTCCAGATTTAAGATATAACTATACTCATCATGAATGGCTACGAGAGCGAGCTATTTTAGCTGCAAAA
AATTTAGATGTAGACGCCATCAATTTTAAAATACAACAGTCATTGCCTGGTAATGAAATTACATTTAAA
TCAATTGACACCGTTGTTAATCCTGACGAAGTGGTCAACTATCCAGTAGAATTTTTGAATTCTTTAGAT
TTACCTGGAATGCCACCACATAATTTGCGACTGAAGATTGGCTCACCTATAATTTTGCTTCGTAATTTA
AATGCCCCGAGATTGTGTAATGGCACGCGATTAGTTATAAAAAAAATCATGGGCAACATTATTGAAGCG
AATATTTTATCTGGAAAATTCCAAGGTGAAATTGTACTTTTACCGCGGATCCCAATGATTCCTTCAGAT
TCACCTATAACATTTAAACGCTTACAATTTCCGATCCGTTTGGCATATGCTATGACTATTAATAAGTCA
CAAGGTCAAACGATGACATTTTGTGGCTTAGATTTAGAAAATCCGTGTTTTTCTCACGGCCAATTGTAC
GTTGCGTGTTCCAGAGTTGGAAAACCATCGAGTTTGTTTGTTTATACTTCTCATGGACTAACTAAAAAT
ATTGTACATCCAATGGCATTACGCTAAATTAAGTTTTTGTAAATTAAAAATAAATAAATATATATGTTA
TAAAATGTTTTGAGTGCAAGCTTTTTCACCTTTCATTAAAATGAGTTAAAGTTTTCATTTACGATGCTG
TTATTTATTTAACAAAGTAATAACTTATCAATAATAATTTTCATCAAAGCGGTCCAGATTGTTTCAGCT
CATTAAAAAAAGTATGAAATTAAAGACGTGTGTACAGGACAACGTCTGTCGGGTCCGCTAG

>Bger_Hel1Aa

TCTTAATATATATATTTCTTGTGTGCGTGTGTATGTGACTGAACTCCTCCTAAACGACTGGACCGATTT
TGATGAAATTTTTTGTGTGTGTTCGTGGAGATTCGAGAATGGTTTAGATTTACAGTTTGGTCTACTGGA
AAATGTTTTTTCAATTAATTTCTTATTTATAAGGAGTTGTTGATTTTGGAATGTTTTACATTAGATCCG
GCGGACGGCGCTATCATCGCATTCAATATTATTCTATTTCAATTTTAGTTTGTCCCGACAGATGGTGCT
ACGATTAATTTGAAAAA

AGAAAATCCGTGTTTTTCTCACGGCCAATTGTACGTTGCGTGTTCCAGAGTTGGAAAACCATCGAGTTT
GTTTGTTTATACTCCTCATGGACTAACTAAAAATATTGTACATCCAATGGCATTACGATAAATTAAGTT
TTTGTAAATTAAAAAAAAATAAATATATATGTTATAAAATGTTTTGAGTGCAAGCTTTTTCACCTTTCA

TTAAAATAAGTTAAAGTTTTCATTTACGATGCTGTTATTTATTTAACAAAGTAATAACTTTTCAATAAT
AATTTTCATCAAAGCGGTCCAGATTGTTTCAGCTCATTAAAAAAAGTATGAAATTAAAGACGTGTGTAC
AGGACAACGTCTGTCGGGTCCGCTAG

>Bmor_Hel1Aa

TCTTAATATATATAATTCTTCTGTCCGTGTGTATGTTAGTGAACTCCTCCTAAACGGCTGGACTGATTT
TGATGAAATTTTTTGTGTGTGTTCCAGTGGGTTCGAGAGTGGTTTAGATTCACAATTNGATATTGTAAT
AACTTTTTTTAAATTCATCAAAACAGTCCAGATTGTTATAACTTATTCAAAAAGTTTGNAATTTTTAGA
TTTAAGATTTGTGTATAGGACAACATCTGTCGGGTCTGCTAG

>Bmor_Hel1Ca

TCTTTATATATATATTTCTTCTGTGCGTGTGTTTGTCACTGAACTCCTCCTAAACGGCTGGACCGATTT
AATGATTTTTTTTGTATGCTTTGGGTGGCCCCTGGATGGTTTAGATTCACAAATCAGCCCGGCAGATGG
CGCTGCAGTCGGTATCTAGGTTATTTATCTTTCCTAGAAATAATTTATATGGCAAAACAACGTTTGCCG
GGCCAGCTAG

>Bole_Hel1Aa

TCTTTATATATTATATATATTTCTTGTGTGCGTGTGTATGTCACTGAACTCCTCCTAGACGGCTGGACC
GATTTTGATGAAATTTTTTGTGTGAGTTCAAGGGGATTCGAGGATGGTTTAGATTACATTTGTCCACTG
GAAAATGTTTATTTAACTAATTTTTCATTTATAAGTAGTTGTTAATTTTGGAATGTTTTAATTGGATCC
GGTAACTGCGCTACCATCGCAGATGTTAGAACGTTTTACATAGAATTCCGGCAGACTGCGCTGCAGTCG
TGACAGATATTCAAAAGTATATTTGAGCCTGGTAGATGGCGCTGAGATCAGATTCTAAGATAGCATAAG
TATAGTTTAAACTATTAAGACTATAGACATGTGTATGGTGTATAGAGTTAAACATTCATACTGCTATGC
TACTCTATACGGAAATAGTGAATATACAAAACTAAGAAATATGTAAATAAATTGTTTATATTTCTTGCT
CTTCCGCGAAGACGTGGCATTTTTTGTAGAAATAAAATACTATGAAAAATACACAAGGACCAATGAGGA
TTAGTCATAAAAAAAATCATGGGCAACATTTTTGAAGCCACTATTTTGAGTGGAAAATTTCAAGGTGAA
GTTGTACTTCTGCCACGGAYTCCAATGATTYCTTCAGATTCGCCGTTACCATTTAAACATTTACAATTA
CCCATCTGCTTGGCATTTGCGATGACTATTAATAAGGCACAAGGTCAAACAATGACGATTTGTGGATTA
GATTTAGAAAATCCGTGCTTTTCWCACGGCCAATTATACGTTGCGTGCTCGAGAGTAGGAAAACCATCG
AATTTATTTGTTTATACCTCTCAAGGATTAACCAAAAATATTGTACATCCTATGGCATTACRATAACTT
CAGTTTTTGTAGATAATTAACTCTAAGAATAAATACCCGTTATAGAATAGATTCAACCTTTTCACAATC
AATTAAAAGACTTCAAGTTTTCATTCAATACAGCTTCTACAATTAACATCTTGAAAGCATTCTAATATA
CATATTGACACGCTATCGATTGGTCGGATTGTGATAAATATATATATATGACGTTAGATATTGTAATAA
ATTTCTAATAAAAATTTTCATCAAAACGGTCTAATTTTACAGCTAATTTCAAAAAGTTTACATTTAAGA
CGTGGGACAGGACAACGTCTGTCGGGCTTCTAG

>Btry_Hel1Ba

TCTTTATATATATAAATCTTCTGACCGTGTGTTTGTAATTGAACTGCTCCTAAACGGCTGGACCGATTT
TGATGAAATTTTTTGTGTGTGTTTAAGGAGATTCGAGGATGGTTTAGATTCACAATTTGGTCCACTGGA
AAATGTTTTTTTAATTAATTTTTCATTTGTAATTAATTGCTAATTTTGGAATGTTTTACATTGGATCCG
ATAGATTGCGCTACCATCGCAGTATCCAATATTCAAACCTTAAATTGGCGTAAACGTGCAATAAACAAA
ACGATGCAAAAGTAAAAGGCGACATCTGTAGACAAGTTTTCATAATGTGGATAGAGTTGTTCGTTCTTA
AGTAATAAATTGGGTGATTCAATACATCTATGGGTAGCTATATCTATATATAAAAAAGAAAGTTGTGTT
AGTTGCACCATTTATAACTCAAGAACGGCTAAACCGATTTGGCTGAAAATTGGTGGAGAAGTAGCTTAG
AACCAGRGTAAGGACATAGGATACCTTTTATCTCTTTATGTCAAAATTTAATACAACTCATAAATACAA
AAATTATATTTCAAATAATAAGCATTTGTTTAAAATTTATGTTTGTAGGAATCATTTGAATATATGCGT
ACAATTATARACAGATTCTTCCTCAAAAATAATACTATTGCTAAGTATTTGGAATAGTAGAAAGGAACT
ACAACCAAATACGCAGTAAAATAAATTATAACTGGCTCAGTAATCAGTCGTTGAGTATGTATTATACCA

CGTGCTCCCAAAAGACTGATGTTATAACCTTTTCAGCCGACTTTTTCGTCTTTCCACGCCTGAGAACCG
GTTCATCATGTGCAGTCCACTAGAATGACAGTCGATTGGACTTCAGTGGGAAATGATGGAGTTATGTTT
CTATTGTCACACACCGACGCAAAAATTCGGTTTTATTACGATTAAAGAGCACTCAAACACTTCTCAGAA
TCAGTTATTCGTTGTTTTTGTTGGATTATGAACTTGCGAGGCACCCACTTTGCACAGAGCTTTCGCCTC
TTTAAGCTTAGCAAATATCTTTTCATCGGTGGATTTCCCTGAGCCCAAATTCAACAGTATTTTCCCCCA
AAAAGCAATGCTTTATTAACACTCGAAATGCCTTATGATCCATTTTTTTAAACTAACACAAGTTGCTTC
ATAAAAATGCTATATCTCATTAACTAATAGTTATAATCCAACTAATAGAAATCATATAGATGGTAGTAG
TAGTATTATCTATGTATCAGCCACAGTGAAGCGTGGACGGGTCCTCTAG

>Cnem_Hel1Ea

TCTTTAATATATATATAATTCTTCTGTGAGTGTGTATGTCACTGAACTCCTTCTAGACGACTGGACCGATG
TCGATGAAACTTTTTGTATGTGTTCTAGGGAATTCGAGAATGGTTTAGATTCACAATTTGGTCCACTGG
AAAATGTTTTTTTTAATTATTTTTTTAATAATAAGTAGTTGTTGATTTTAGAATGTTTCACATCGGATC
CGACAGACTGCGCTACTGTCTAAGCATTAATGTCAAGCGATACGAAGTTTGCTGGGTCAGCTAG

>Csup_Hel1Aa

TCTTTATATATATATAATTCTTCTGTGAGTGTGTATGTCACTGAACTTCTCTTAAACGACTGGACCGATTT
TGATGAAATTTTTTGTGTGTGTTCAAGGGGATCTGAGAATGGTTTAGATTCACAAAGACGTGTAGACAG
GACAACGTCTGTCGGGTCCGCTAG

>Csup_Hel1Ab

TCTTTATATATATATAATTCTTCTGTGAGTGTGTATGTCACTGAACTTCTCTTAAACGACTGGACCGATTT
TGATGAAATTTTTTGTGTGTGTTCAAGGGGATCTGAGAATGGTTTAGATTCACAATTTTGTCCGCTGGA
CAATGTTTTTTTAATTAATTTTTAATTTATTAGTAGTTGTTGATTTTGGAATGTTTTACATTGGATCCG
ACAGACGGCGCTACCATACTAGACAGGACAACGTCTGTCGGGTCCGCTAG

>Csup_Hel1Ea

TCTTTATATATATATAATTCTTCTGTGAGTGTGTATGTCACTGAACTTCTCTTAAACGACTGGACCGATTT
TGATGAAATTTTTTGTGTGTGTTCAAGGGGATCTGAGAATGGTTTAGATTCACAATTTTGTCCGCTGGA
CAATGTTTTTTTAATTAATTTTTTTTATGGCGGTACGAAGTTCGCTGGGTCAGCTAG

>Cves_Hel1Aa

TCTTAATATATATATATTTCTTGTGTGCGTGTGTATGTGACTGAACTCCTCCTAAACGACTGGACCGATTT
TGATGAAATTTTTTGTGTGTGTTCAAGGGGATCTGGGAATGGTTTAGATTCACAATTTTGTCCGCTGGA
CAATGTTTTTTTAATTAATTTTTAATTTATTAGTAGTTGTTGATTTTGGAATGTTTTACATTGGATCCG
ACAGACGGCGCTACCATCGCAGTGTCAAATTAAAGACGTGTAGACAGGACAACGTCTGTCGGGTCCGCT
AG

>Cvic_Hel1Ca

TCTTTATATATATATAATTCTTCTGTACGTGTGTTAGTAACTGAACTCCTCCTTAACGGCTGGGCCGATTT
CGATGAAATTTGTTGTGTGTGTTTGAGTGGGTCCCTGGATGGTTTAGATTCACAATTGACCTATATAGG
AACAATGGGCATGGCACCTCCCATACAAAGTAAAAATTTATTATTGCATATCTGGAGTACTATTATAGT
GGGAGTCTTCAAACTTTGTGTGAGCTATGGCAGAACAACGTTTGCCGGGACAGCTAG

>Cvir_Hel1Ea

TCTTTATATATATATAATTCTTGTGTTCGTGTGTTTGTCACTGAACTCCTTCTAGACGACTGGACCGATTT
TGATGAAACTTTTTGTGTGTGTTCAAGGGAATTCGAGAATGGTTTAGATTCACAATTTGGTCTCCTGGA
AAATGTTTTTTTAATTATTTTTTTAATAATAAGTAGTTGTTGATTTTGGAATGTTTCACATTGGATCCG
ACAGACTGCGCTACTATCGAAGCATTAATGTCAAGCGATACGAAGTTTGCTGGGTCAGCTAG

>Daus_Hel1Aa

TCTTTATATATATATATAATTCTTCTGTGCGTGTGTATGTCACTGAACTCCTCCTAAACGGCTGGACCGAT

TTTGATGAAATTTTTTGTGTGTGTTCAAGTGGATTCGAGAATGGTTTAGATTCACAATTCGGTCCACTA
CAAAATGTTTTTTTAATAAATTTTTTATTTATAAGTTATTGTTGATCTTGGAATGTTTTACATTGTATC
CGGCAGACAGCGCTGCGATCGCGGTGTCGAATATCCAAAAATATTTCATTTTACTTCCAGTTTGTCCCG
ATAGTGGCGCTGCGATCAGATTCAGGAGAATTTCGTAACTTTGTGTTGTTATTGTGTTAAAATAAATCG
CGTGCTAATTGAAATATTACATTCAGATGTAATTAGTGTGTGAAGGGTTTTGAAGTGTAGATAAAAACT
ATACATTGTGCAAATTTAAGTTTCTATTCAACATAGCTTCTATATTCAACTTAGCTCATAGTATATACA
TATTCACACGCAATCGATAGGTCGAATTGTGGTAACTGTACATGTGATGACGTCAGGTATTGTAATAAC
TTTTTTTATAAAAATTTTCATCAAAACGGTCCAGATTGTTACAACTTATTAAAAAAAGTTTGAAATTTT
CGGATTTAAGACATATGTACAGGACAACGTCTGTCGGGTCCGCTAG

>Hvit_Hel1Ga

TCTTTATATATATATATTTCTTGTGTTCGTGTGTATGTGACTGAACTCCTCCTAAACGACTGGACCGAT
TTTGATGAAATTTTGTGTGTGTTTTCAATGGAATTCGAGAATGGTTTAGATTCACAATTTGGTCCAATT
AAATTGTTTTTTAATTAATTTTTATTTATAAATTGTTGTTGATTTTGGAGTGTTTTACTTGATCCGGCA
GACTGCGCTACGACACGTAATACAAAGTGAACTGATACTTAACAGCTGTTAATACTTTCAGCTGAAGTT
CATCAAAGAGGCAGAAACATTTGTTTACATTTAAAATTTAGAAAATTATTATTGTAAAGTGCTTGATCA
GTAGTGTAATGCAGATTGCATAGAAGAAGTTATTGCCTAATTTTAAATTTAGATTGCACCAATGATCAA
TAAACTATCTAATGCAATGAAAATACTATTAAACGCTGTTATAAAAACCACCTCATTGTACAAAGCCGT
GAATGTTTGCACATAAGGTAATCGAATTTGGTTAGATCGAAGGTAAATGAAAAGAGCCGAAAGAAGACG
CTTTATGATCGAAATTGGTTTTCGTTGATACATTTTCTTGATCGGAGCACAAGGCAAACTCCACAATAA
TACTGGAAATATCTTAGACAGAAAATTAATTTTAACAGACCGAATTTGATTCTTTATAACCTAATTAGT
TTATCGAGATTCATCCATATAAATTAATTGTACTGAATAACTTATCAACACCTAGCAAAAACCACGAAA
GATAGAGGCAGGAAATATGGTACATACCTTCATAATGCGCCCAAGAGGCTCACGAAGGAACGACTATCA
ATTATCTCAGGAATGTTTAGAATGTGATAGAAAAAGAATACGTGAATATAGTGTACTGTTTTTCAACAG
GAAATTGCGTGCGAAGCCGCGGGCAACTGCTAG

>Lmig_Hel1Aa

TCTTAATAATATATATTTCTTCTGTCCGTGTGTTTGTCACTAAACTCTCCCTAAACGGCTGGATCGATT
TACATGAAATTTTTTGTGTGTGTTTAAGGGGATTCGAGAATGGTTTAGATTCACATTTTGGACCACTGC
AAAATGATTTTTTAAATTAATTTTTTATTTGTAAGTAGTCGTTGATTTTGGAATGTTTTACATTGGATC
CGACAGACTGCGCTACCATCGCAGTATCAAACATTCAGTAATATCCTATTTTATTTTTAGTTTGTCCCG
ACAGTTAGGGCTGCGATCAGATTGAGAAAATATTTCGTAATCTCTTTATCACATTTACGTGTATTCCAA
ACTGGGATGAAATTAAAAATTTGCTGTTGTCAGGTCAAACATCGATGCATCGCCATGATATCACTACAC
GTGTCTTTAAACAAAAAATAAAATATTTGATGAATTTGATTACACATCACTCGGTCTTTGTGAAACACG
TTGTTGGCTTTACTCTGTTGAGTGGCAAAAACGAGGTTTACTTCATGTGCATATTTTAATTTGGTTCGT
GGATAAAGTACGCCCAGAAGAAATTTAGTGCGGAATCGAAGTCAGTATATTTATTATTTTTTTATTAAA
TTTATCCGTGTGTTGATTAACAACCACCCTATTTCAACTTCGATGAGTTACTCATATTCGTTGTGTATT
TTTTTGCAGTATGCCACGTCTTCGTGGAAGAGCAAGAAATATTGGTCGACGTACTCGGAACGCGCAATT
GGTCCATGATCGTCGATTAAATAGAACAGTTGAAGAACACTGATGGATAACAGAAATGTAAGAGATCAA
GTTGCATGTACACGTGAAAATGTAAATTCCGAACATCGCACTCAACGTCTTCGTGCTAATTCTTTAAGA
CAACAAGACGCACGTCAACAAGCGACCAACGCACATAGAGAACGTGACCAGCGGCGATGCAAGAAGACG
AGTATTGGCACGAGGATCGCTTAATCGTCTCGCGTTTGAATATGATCCTGAAATAGATTATTCATCACA
TCCATTAATCACGATCGGTACTATGGATAAAAAGTGTCAACATTGTCATGCTTTCAAGTACCAAGCTTA
ATCAGCTGGTTTATGTTGCGCATCTGGAAAAATATCACTGCCACCGCGAAAACCACCGCCGGAACCTTT
AAAACCACTTTTAGCTGGAATCTCATCTCAATCGAAATTGTTTTTGCGGAAAATCCGTAAATTCAATCC
ATGCTTCCAAATGACATCATTTGGAGCAACAAAAATTGTTCATAATGAAGATGGTCGTAATTTTGAATC

TACATTCAAGATTAAGGTCAAGTGTACCACCAAACTGGTTCATTGCTTTCAATGCCTGATGCCGATCCT
AAATTTTTACAAATTTACTTCATGGGCGATGAGGAGCAGCAAATCATAGGCAACATTCATGAAGCAACT
ATTTTAAGTGGAAAATTCCAAGGCGAAGTTGTACTCCTACCACGAATCCCAATGATTCCTTCAGATTCA
CCTATACCATTCAAATGTTTACAATTTCCAATCCGCTTGGCATATGCTATGACAATAAACAAGTCACAA
GGCAATGACAATTTGTAGCTTAGATTTAGAAAATCCGTGTTTTTCTCTCGGCCAATTATATGTTGCATG
TTCCAGAGTTCGAAAACCATCAATTTTTTTTGTTATACTCCTCAAGGCTTAACTGAAAATATTGTACAT
CCAATGGCATTACGATAAATTAAGTTTTTGTAAATTAAAAAATATATGTTATAAAATAGTTTAGTGTAA
TTTTTTTACCTTCAATTAAAACAAGTTAAAGTTTTCATTTAAGATACTGCTATTTATTTAACACAGCTA
TTCGGCATTTCACTTCACTCCTACAGTATTCACATACTCACACGCTATCAATTGGTCGAATTATGATAA
ATGTACATGTAATGACGTTAGATATTGTAATAACTTTTTAATAGTAATTTTCATCAAAACGGTCCAGAT
AGTTTCAGCTTATTTAAAAAAGTTTGAAATTTTCGAATTAAGGATGTGTGTACAGGACAACGTCTGTCG
GGTCCGCTAG

>Mcin_Hel1Ca

TCTTTATATATATATATTTCTTGTGTGCGTGTGTATGTCACTGAACTCCTCCTAGACGGCTGGACGGAT
TTTGATGAAATTTTTTGTGTGAGTTCAAGGGGATTCGAGGATGGTTTAGATTCACAATTTGGTCCACTG
GAAAATGTTTATTTAACTAATTTTTCATTTATAAGTAGTTGTTAATTTTGGAATGTTTTACATTGGATC
CGGTAGACTGCGCTACCATCGCAGCATCAAATATTAAATATTATTCTATTTTAATTTCAGTTTGTCCCG
ACAGTTGGTGCTGCGATCAAATTGAGAAAAATATTTGAAATTTTGTGTTATGGCAAAACAACGTTTGCG
GGGTCAGCTAG

>Mcon_Hel1Aa

TCTTAATATATATATATTTTCTTGTGTACGTGTGTATGTCACTGAACTCCTCCTAAACGGCTGGACCGA
TGTTGATGAAATTTTTTGTGTGTGTTCAAGGGGATTCGAGAATGGTTTAGATTCACAATTGGACCCGGT
AGGTGGCGCTGCAATCGATTTCTATAATTTTTCGTCATACACAAAGCAAACGTTCGGACAGGACAACGT
CTGTCGGGACCGCTAG

>Mper_Hel1Fa

TCTTTATATATATAATTCTACTGTACGTGTGTATGTCACTGAACTCCTCCTAAACGGCTGGACCGATTT
GAATGAATTTTTTTGTATGCGTTTGGGTGACGCCCTGGATGGTTTAGATTCACAATTGGACCCGATAGG
TCCGACCCGGGGAGTTGCTCAAACAGGGATTTTAAGATTTACGAAGGAAATATTTGTTTATATACGGTT
GCTATTGGTTATAGGAGAAATATAAATATATTTAGTAGAAATTGGTATTTTTTTTTTTGTTTATTGGTT
GCCTGCAGTGGTTCATCGGGCAGATCAGGTATAAGCATGCATACAATTGAATTTTCGCACATTGCCTAC
CAAGGTGGCTGAGTTGCACTTATAGCCGCTCATCGGCGTGTCCGCAATCAAAATAAAAATAAAACATAT
TATTTATATAAAGTACTCATCAAGCGGGGTCCGCAATCCACAGCAGGTAGGCATAATATACTGCTCGCA
TTATCACCATCGAGTCTCATATAGAGTTGGCATTTTTAACGGGCAACAAAGTGCACGGGATCAGCTAG

>Ncla_Hel1Aa

TCTTTATATATGTAATTCTTCTGTGCGTGTGTATGTAACTGAACTCCTCCTAAACGGCTGGACCGATTT
TGATGAAAGTTTTTGTCTGTTCCAGTGGACTCGAGAATGGTTTAGATTTATAATTCGGTTTAGAAATGT
TTATTTAATTAATTTTTTATTTATAAATTGTTGATCTTGGAATGTTTTACATTGGATCCGGCAGACTGC
GCTACGATCGCAGTATCGAATATTCAAAAATCTTTTATTTTGAATTTAAGATGTGTGTACAGGACAACG
TCTGTCGGGTCCTCTAG

>Obru_Hel1Ia

TCTTAATATATATAATTCTTCTGTACGTGTGTATGTCACTGAACTCCTCCTAAACGGCTGGACCGATTT
TGATGTTTTTTTTGTGTGTGTTTGAGTGGGTCCCTGGATGGTTTAGATTCACAACTGGACCCGGTAGGT
GGCGCTCAGTCGGGTCACCGGGACCACGCGAACGAAGTCGCGAGCGGGCCGCTAG

>Pdom_Hel1Aa

TCTTTATATATATATATATAATTCTTCTGTGCGTGTGTATGTCACTGAACTCCTCCTAAACGGCTGGACCG
ATTTTAATGAAATTTTTTGTGTGTCTTCAGGTGGATTCGADAATDGTTTAGATTTACAATTCGGTTCAA
TTTAAATTGTTTATTTAATTAATTTTTATTTATAAATTGTTATACGACAAGTTTATAGGTCGATATTTT
TTGTTGATATGATACTTTTCGATATATTCGATATATTTCTCTTATTTCGTATCTTCTTGACCTATCTTT
GACCTTTTTAATAAAAATTTTCATCAAAACGGTCCAAATTGTTACAGCTTATTCAAAAAAGTTTGAAAT
TTTCGAATTAAAGACGTGTGTABAGGACCAACGTCTGTCGGGTCCGCTAG

>Pgla_Hel1Ga

TCTTTATATATATATTTCTTGTGTGCGTGTGTATGTCACTGAACTCCTCCTAGACGGCTGGACCGATTT
TGATGAAATTTTTTGTGTGAGTTCAAGGGGATTCGAGGATGGTTTAGATTCACAATATATATAAGGAGT
GCGTGCGAAGCCGCGGGCAACTGCTAG

>Pgra_Hel1Aa

TCTTAATATATATATTTCTTGTGTGCGTGTGTATGTCACTGAACTCCTCCTAAACGACTGGACCGATTT
TGATGAAATTTTTTGTGTGTGTTCAAGGGGATTTGAGAATGGTTTAGATTCACAATTTGGTCCACTGGA
AAATGTTTTTTTAATTAATGA
AAACCATCGAATTTGTTTGTTTATACGCCTCAAGGATTAACCAAAAATATTGTACATCCAATGGCATTG
CGATAAATTGAGTTTTTGTAAATTAAAAAATATATGTTATAAAATAGTTTGAGTGTAAGCTTTTTCACC
TTTAATTAAAACAAGTTCAAGTTTTTATTTATGATGCTGCTATTGCTACACTCCACAGATAATATCCGG
GCCTGTAATCAGTCGTTTCATTGATTTAAAATTTTCCGTGAGTCTTAGAAATATCCGTGTTTGTAATCA
AAGACATACAGTGGCTGCTATTTTTTGATCCACAGATTCCATCTGTGGAGTGTGGCAATAAACACATCT
TTTTATTTAACACAGCTTCTACATTTCACTTCACTAATACAGTATACACATACTCACACGCTATCGAAT
GGTCGAATTGTGATAAGTGTACATGTAATGACGATAAAGATTGTAATAACTTTTTAATAATAATTTTCA
TCAAATCGGTCCAGATTGTTATAGCTTATTAAAAAAAGTTTGAAATTTTCGAATTAAAAACGTGTGTAC
AGGACAACGTCTGTCGGGTCCGCTAG

>Pmac_Hel1Ha

TCTTAATATATATATTTCTTGTGTGCGTGTGTATGTGACTGAACTCCTCCTAAACGACTGGACCAATTT
TGATGAAATTTTTTGTGTGTGTTCGTGGAGATTCGAGAATGGTTTAGATTTACAGTTTTTTTTTTTTTT
TTTTTAATTCCGCGCGGACGGAGTCGCGACCGACAGCTAG

>Prap_Hel1Aa

TCTTAATATATATATTTCTTGTGTGCGTGTGTATGTGACTGAACTCCTCCTAAACGACTGGACCGATTT
AGACGAAATTTTTTGTGTGTGTTCAAGGGGATCTGGGAATGGTTTAGATTCACAATTTTGTCCGCTGGA
CAATGTTTTTTTAATTAATTTTCAATTTATTAGTTGTTGTTGATTTTGGAATGTTTTACATTGGATCCG
ACAGACGGCGCTACCATCGCAGTGTCAAATTTTAAATAATATTCGAATTTTAATTTTAGTCTGTCCCGA
AATTTAAAAAAAGTTTTGTTATCATTGTGTTATATCGTGTGTGACCATGTGCTGGATCGTTAGATATTG
TCATAACATTTGAATAATAATTTTCATCAAAATGGCTTATTAAAAATTGAAATTTTGAAATTAAAGACG
TGTAGACAGGACAACGTCTGTCGGATCCGCTAG

>Ptep_Hel2Ca

TCTATATATATAAAAATCTCTTGTCACAGTTTTAGTGTTTAAACTCCTCCGAAACGGCTTGACCGATTT
TGATGAAATTTTATATGTATATTCAGTAGGTCTGAGAATAGGTTTATAACTAATTTTTTTTTCATTTTT
TGGACAGATTTAACGTATATTTTAAATATATCTTTATTTAATCGTATTCGGATCCAAACTAGACATAGA
AACATAATAAATCAAAAATAATATTCACGCACGTTGCAACAAGTCATATTAAACTCTAATACTTATGCT
CGCCCTTAAAAGAAGAGCATCAAATATATTTGCAAGTATGTGAATAAAGGCAGTGATATGGCAGTATTT
CGAGTTGAAAATACCAATGTGACTGCTCTTCCAGTGAATAACAACGACGAAATAACGCTGTACCAAATT
GGCCGGTACATCAGCTCCAATGAAGCTGTTTGGCGTATCTTTGGTTTCCCAATTCATGAACGGGATCCA
ACCGTTATTCATTTAGCCGTCCATCTTGAAAACGGTCAGAGCGTATATTTCACGAACGAGACAGCGATT

GACCGAGCTATAAATCCACCAAAAACTACACTCACCGAATTTTTTGAATTGTGTAATCGTGCGGATGCT
TTTGGTGCCTTTGCACGGACATTACTCTACTCAGAAGTACCACACTATTTCACATGGGCTCAAACAAAA
AAATGGACACCCCGCAAGCAAGGCACACCGGTTGATGCATGTCCCGGTTTATTCAAATCAAACGCCTTG
GGGAGAGTATTTACAGTCAATCCAAGGCAGACTGAGTGCTTTTATTTTCGACTGTTGTTGGTTAATGTC
ACCGGCTCATTGTCATTTCAAGATATACGTAAAGTCGATGGACATCAGCATCCAACGTATAAAGATGCA
TGCCTTGCACTCGGCTTGCTGGAAGACGACAACCAGTGGGAATGTATGCTTGCTGAAGCAGCATTGAAC
TGTACTGCAAAGCAAATTCGTCTACTATTCGCTATAGTATTAACTACATGTTTCCCGGCCCGTATAGAA
ACGTTATGGGATAATCACAAAGATTCGATGACTGATGATATACGGTATCAACATCGCACACGTTGCAAC
GATCTAACGATAGCATTCAGCGATGCTATGTACAATGAAGCACTGATTGCTATTGAGGATCTTTGCATT
ATTATTGCCAACTTGCCACTCAGTCATTTCGGTATGCCTTCGCCAAATCGAAGTGCATCTGATTTATTA
AACACTGACATGAATCGTGAACTTCAATACAATACGTAGAAATGGCAGCGATTGTTACTCGCAATGTTC
CACTAATGAATGAGGAACAAAGGAACATTTATGACCGCATTATGCTCGCAGTTTCGGCAGGACAAGGTG
GATTTTTTTTTGGATGCACCAGGTGGAACTGGCAAAACATTTATTATTTCGCTAATTCTCGCTGAAATA
CGATCAAATAATGGCATCGCGTTGGCCGTTGCATCATCTGGCATTGCGGCAACTTTATTGGATGGAGGC
AGAACAGCTCATTCAGTATTTAAGCTGCCACTAAATATTCAAAACAACCCAGACGCAGTGTGCAACATA
AAAAAGCAATCGTCTATGGCCACAGTGTTGAAACACTGTAAAATTATCATCTGGGATGAATGCACTATG
GCACACAAACATTCGCTTGAGGCGTTGAACAGGACATTGAAAGATATAAAAAACAATGACAAATTATTT
GGCGGCACTCTGTTACTCCTTTCAGGTGATTTCAGACAAACACTTCCCGTCATTCCGCGTTCAACGTAC
GCTGATGAGATCAATGCTTGCTTGAAATCATCTCCACTGTGGCGTAATGTTGAAAAAGTTCAACTAAAA
GTAAATATGCGCGTCCAAATGCTTCAAGATCCATCTGCTGAAACATTCTCAAAACAATTGTTAGATATC
GGTGATGGAAAAGTTACTAAGGATGAGACTGGATGCATAAAATTACCGGACGATTTCTGCACAATCATT
GATTCACAAGATGCTCTCATTAACCTAATATTTCCCGATGTACACACGCAATACATTCATCATGAGTGG
CTGGCAGAAAGGGCGATTTTAGCAGCAAAAAATGTGGACGTCAACGAATTGAATCTGAAGATACAACAG
TTATTGCCAGGTAACTTGGTGACATATAAATCCGTTGATGCAGTTTGCGATCCCATTGAAGCTGCAAAT
TTTCCAACAGAGTTTTTAAACTCATTGGTTTACCAGGCATACCACCGCATAATTTAGTACTCAAAGTTG
GATCTCCGGTTATTTTGCTTCGTAATTTGAACCCACCACGGCTGTGCAACGGCACGCGATTAGTCGTTA
AAAAATTAATGAACAACGTTATCGAAGTCATTATTTTAAATGACAAATTCCGGGGTGAAAGTATATTAC
TTCCACGAATCCCTATTATACCCACAGATGTGCCAATTCAATTTAAACGCATTCAGTTCCCCATTAGAT
TGGCATTTGCAATGACAAGGCCAAACAATGTCTGTTTGTGTCTTAGATTTGAGCACACCATGTTTTTCA
CACGGACAATTATACGTGGCATGCTCTCGAGTGGGCAAACCATCCAGTTTATTTGTGTTAGCTAAAGAT
GGAATGACAAAAAATATTGTACACTCCATTGCACTAAGAGATTAATATTGTTGACTAATATTTTCAGAA
TTCGTTGTATATATAATTTATAAAAAAAAGTTACAATAAATTAAAAAAATATTATTTGGTGTGTTGTTA
TTTTCACATTCCGTCATCGTTCACAGCGCTCCATGCCTCTTCCACTCATATACCACATACGCACACACT
TACAATAAATAAGATATATTTTCATACTCTAGAAATAATTCATATGGCAAAACAACGTTTGCCGGGTCA
GCTAG

>Pxut_Hel1Aa

TCTTTATATATATAATTCTTCTGTGAGTGTGTATGTCACTGAACTTCTCTCAAACGACTGGACCGATTT
TGATGAAATTTTTTGTGTGTGTTCAAGGGGATCTGGGAATGGTTTAGATTCACAATTTTGTCCGCTGGA
CAATGTTTTTTTAATTAATTTTTAATTTATTAGTAGTTGTTGATTTTGGAATGTTTTACATTGGATCCG
ACAGACGGCGCTACCATCGCAGTGTCAAATTTTAAATAATATTCAAATTAAAGACGTGTAGACAGGACA
ACGTCTGTCGGGTCCGCTAG

>Pxut_Hel1Ab

TCTTTATATATATAATTCTTCTGTGAGTGTGTATGTCACTGAACTTCTCTCAAACGACTGGACCGATTT
TGATGAAATTTTTTGTGTGTGTTCAAGGGGATCTGGGAATGGTTTAGATTCACAATTTTCATCAAATAT

TAACTTCATATTAATTTATTCAAATTAAAGACGTGTAGACAGGACAACGTCTGTCGGGTCCGCTAG

>Pvar_Hel1Da

TCTTAATATATATATTTCTTCTGTGCGTGTGTTTGTCACTGAACTCCTCCTAAACGGCTGGACCGATTT
TGATGAAATTTTTTGTGTATGTTCAAGGGGATTCGAGAATGGTTTAGATTCACAATTTGGTCCACTGAA
AAAAGTTTTTTAAATTAATTTTTCATTTGTAAGTAGTTGTTAATTTCGCAAGCACAAAACTACAAAAAC
GTAACATGCTTTGCTTCTTCTTACACTTTTGTATTGGTATGTGTGTGTGTTGTTTGTAATGAGAACTCA
AAAACGATGAGGAATTGGGGAACGATAGAATAGAATAAGGGCTAGAAAGCGCGCCGGGTCATACTAG

>Sfru_Hel1Aa

TCTTTATATATATAATTCTTCTGTAAGTGTGTATGTCACTGAACTTCTCTTAAACGACTGGACCGATTT
TGATGAAATTTTTTGTGTGTGTTCAAGGGGATCTGAGAATGGTTTAGATTCACAATTTTGTCCGCTGGA
CAATGTTTTTTTAATTAATCAGACGTGTAGACAGGACAACGTCTGTCGGGTCCGCTAG

>Sfru_Hel1Ab

TCTTTATATATATAATTCTTCTGTAAGTGTGTATGTCACTGAACTTCTCTTAAACGACTGGACCGATTT
TGATGAAATTTTTTGTGTGTGTTCAAGGGGATCTGAGAATGGTTTAGATTCACAATTTTGTCCGCTGGA
CAATGTTTTTTTAATTAATTTTTAATTTATTAGTAGTTGTTGATTTTGGAATGTTTTACATTGGATCCG
ACAAATTTTCAAATTAAAGACGTGTAGACAGGACAACGTCTGTCGGGTCCGCTAG

>Sfru_Hel1Ac

TCTTTATATATATAATTCTTCTGTAAGTGTGTATGTCACTGAACTTCTCTTAAACGACTGGACCGATTT
TGATGAAATTTTTTGTGTGTGTTCAAGGGGATCTGAGAATGGTTTAGATTCACAATTTTGTCCGCTGGA
CAATGTTTTTTTAATTAATTTTTAATTTATTAGTAGTTGTTGATTTTGGAATGTTTTACATTGGATCCG
ACAGACGGCGCTACCATCGCAGTGTCAAATATTAATGACGTTAGATATTGTCATAACATTTGAATAATA
ATTTTCATCAAAAAGGTCCAGAATGTTTTAGCTTATTAAAAAAAGTTTAAAATTTTCAAATTAAAGACG
TGTAGACAGGACAACGTCTGTCGGGTCCGCTAG

>Tcri_Hel1Aa

TCTTAATATATATAATTCTTCTGTACGTGTGTATGTCACTGAACTCCTCCTAAACGGCTGGACCGATTT
TGATGAAATTTTTTGTGTGTGTTTGTGTGGGTCCATGGATGGTTTTGATTCACAATTGAACCCGGTAGG
TGGCGCTGCAATCGGGTTCTAGGATTTATTTTTATTTTTTAGGGAATTTATTTGAGGGGATTTTTTTTA
AAAGATTTTTTGCATATCAGAACATGTAACCTCACAATTTCACTCCCCCCCCCCCCCCCCAATATTTAT
AATAATGAATATAATATGAATAATTGACCGTTAAAACCTTCTGAATAGTGAATAATTGAACTTCTGAGA
ATAATTCATAGTTATTTTGTGTAACATAGGTGGCATTGATTTCGAATTCTGTGGTATTTGACGTACGCA
TAATTGAACTTCTGAGAATAATTCATAGTTATGTTTGTGTAACATAGGTGGCATTGATTTCGAATTCTG
TGGTATTTGACGTACGCATCAATACATGCGCAAGTCTCTTGCGTCGTGGCTTGGCGTTGTTTTGACATT
TCAATTTCTGTACGATGTGTTTCGATAATCCATGTATCACAATCGCGTGAATATTGAGTGTATTTTTCA
TCAAGTGTACTCAAATACATACATTGGGAAAATGTGTAAGGTGCTGTGTTGAAGTGAATATCTTACATG
ATCTTCCATAAATTTAAATTATATACATGCGTATTCAATGACAATCAATCTAACCTGCAAATTTTTATT
TTAAGTGCTTATTTGTGTCGATGATTTTATAAAATTTACGGAGTGTTAAACGTTTCATCGAAAAAAAAA
CATGTAATCCATAAAATTCAAATATAAGTTGCTTATATTTAGGTGTTGTGTGTTTATTAAAGAACAATG
CCACGTCTTCGTGGAAGAGAAGGAAATATTGGTCGACGTACACAGAATTCACAAATAGTCCACACCTGT
CGGTTAAATCGAACGGCAGAAGATCAATTGACGACAATGCAAATTTAAGAGTCCAGGTTGCAAACACAC
GTGCAAATGAAAGTCAACAGCAACGCAACGAACGCCTTCGAGCTAATGCATTGAGACAAAGACAGGCGC
GTCAAGAGCCACTGACGACACAGAGCACATGAACACAGCGCCTGCAAGTACATCGCGCATTACACGCGC
ATCACTTCTCCGTCTTGCGTTCAATATGAGTCGGACATCGACTATTCGTCACATTCACAAATTGTCACG
GCGCTATGGACAAAGAATGCCAATGCCATGCATTCAAATACAAAGGTGAATCGGCCGGTTTCTGCTGCG
CGTCTGGAAAAGTTGTACTTCCACCATTGAATTGCCGCCAGAACCATTGAAAACACAATTGGCTGGAGC

CACACCTCAATCAAAATTGTTTTTGCGTAAAATTCCAAATTCAATTCTTGCTTCCAAATACATCATTTG
GAGCAACAAAAATCGTTCAGAATGAGGATGGTCGCAATTTTGAATCAACGTTTAAGATTCAGGGCCAAG
TATATCATCAAATAGGTTCTTTAATCTCAATGCCTGATGCCGATCCAAAATTCTTGCAAATCTACTTCA
TGGGCGATGAGGAGCAACAAATAAACGCACGCTGCCAGTACAACCACATCGAGCAGATGGAGGAGCGAG
AAATTGTGGCGACTTTGGAACAGTTTTGCGAAATCACAACCAGTTGGTCCAGTTGTTTTATACCGTTTC
AAACAACTGCAAAACGACAACTACATGATCGTCAAAAGCAGACAAAGTACCATTTGGAGAGCATGCGGG
CGATACAATCACACCGTTAATGAAGTTGCAGTTGTTATGGTTGGCGACGCATATGAACGTCGAGATATA
CGGATCCAACACAGAGATAACACGGTTCACACAATTCAAGACAGTCATCGTTCTTACGACGCTTTGCAA
TATCCGTTGATATTTTGGGAAGGAGAAGATGGATATCATTTTAACATCAAACAAATAAATCCAGTGACA
GGTACAATCTACATATTATTTACTTTTTCTTTTTCTGTAAGATTAATTTCTTTTTGTCTTATTTATATT
TTTACTTTGCTGGTGCAGAAACCAACAAGAAAGTGAGCGCGATGAACTTCTACGCATATCGGTTGATGA
TTCGTGCCAATGAAGACAACAACATTCTCCGATGCCTCCAGCTGTTTCATCAATACATCGTTGATATGT
ATCCGAAAATTGAAAGCGAGCGATTACGGTACATCAAGTATAATCAAGCAAGCTGCGTTCTGAGGAGTA
CATTCATTTGCGTGATCCTATTGTCGGTAACGTCGGTAACGTTGATGGAACCACTAATAGCAATGACAT
CGTTACCGCATATATTCTTTCATCATCATATATTGGTAGTCCGCGTCATATGCAAGAATACATTCAAGA
GCCATGACTTTCGTGCGCGCGTACGGCCGACCAGATTTGTTCATTACATTTACGTGCAATCCTAACTGA
GATGAAATTAAAATTTTGTTATTGCCAGGTCAAACAGCAATGCATCGCCATGATATCACTGCACGTGTC
TTCAAACAAAAATTAATATCGTTGATTAACTAGATTACACATCAATCTGTTCATTTATACACCTCAGGG
ATTATACGAAAAACATTTTCATCAACTCGCATTAAGATAAAAACAAAACTAACTTTATTGATTTTGTCG
GACCGTTTTTATAACATCACAACCTTAGGTATAAGAGGGGGTGAGGTTTCTATGCAGCTGACTGTACGT
ATCATCGAAATAGTATGTACTTCGTACAACAAGTATATACTATTTAGATGGTACGTATATAGGTAAGTT
GTTCTGCTTAAGCCCGTAAAGGTTCTAGGCCCAAAACAGTATATGTTTTGCACTATGAAAATAGAAACA
ATTAAAAAGTAATTTGACATTTGTCATATTTGAAGGTACGTCAATAAAATAAAATTAGCTGTACCCGGC
ATACAAATTTGTAAGCAAGCTTCGCAATTTTTTTCGATACAGCTGATATTTGTGGAGTTGTGGGCGAAA
TTCCAAAAATTGTTGGGATCACTATTCCATATATTATTATTTTTAATAATGGATACCATGTATGTATAA
GTGTCTAGCAGTTCCTATTCTAAGTAAAATTTATTAGTTTCAGAAACAGACAGACGGAATGGGAAAGCA
TTGCCATTACTGTGAAACAAAGTATTTGTAATGTACTATTGTCTCAATATAGTTGATTTTGAGGAAAGA
AAAGATGTTTTATTTTGTATGTTGTCTTGACAAACCCTGAATTTTTAATATTTGTGTAACTCACTAATT
GGAGTTCATTGTGAATTTGTAATTGTGATGATCACATAGTGTTTGTTTTATTATCGAAATCAATTATGC
CACCCAAACGAAAAGCCAACGTTCGGGCAGGACAACGTCTGTCGGGTCCACTAG

Data S5. The sequences are used for evolutionary analysis.

>HeligloriaAi_Dw1

TCTTAATATATATAAATCTCGTGTCACAATGTTTGTCCTCAATGGACTCCTAAGCCACTTAACCTATTATAATAAAATTCGCACACCATGTGCAGTTTGATCTAACTTG

AGA--------GATTTAATTTT----------TCAAAAGCTGG--------------------------CACAGCGAAGCGTGGCAGGGTTTCTAG----

>HeligloriaAi_Rp1

TCTTAATATATATAAATCTCGTGTCACAATGTTTGTCCTCAATGGACTCCTAAACCACTTAACCGATTATAATAAAATTCGCACACCATGTGCAGTTCGATCCAACTTG

AGAGATAGGATAGTTTAAATCTCAAATTATAGTCGCAATTTTATAATGTAATATAACAAAAACTTTAGCCACAGCAACGCGTGGCCGGGT---CAGCTAG

>Agla_Hel1Aa

------TATATATAATTCTTCTGTGCGTGTGTATGTCACTGAACTCCTCCTAAACGGCTGGACCGATTTTGATGAAATTTT----TTGTGTGT-GTTCAAGTGGATTC

GAGA------ATGGTTTAGAT------------TCACAATTCAT-------ATGAATTAATAACGTTA--CAGGACAACGTTT-GTCGGGT---TCG----

>Aros_Hel1Aa

------TATATATATTTCTTGAGTGCGTGTGTATGTGACTGAACTCCTCCTAAACGACTGGACCAATTTTGATGAAATTTT----TTGTGTGT-GTTCGTGGAGATTC

GAGA------ATGGTTTAGAT------------TTACAGTTTAA------AATGAATTAATGATTTTA--CAGGACAACGTCT-GTCGGGT---CCG----

>Bger_Hel1Aa

------TATATATATTTCTTGTGTGCGTGTGTATGTGACTGAACTCCTCCTAAACGACTGGACCGATTTTGATGAAATTTT----TTGTGTGT-GTTCGTGGAGATTC

GAGA------ATGGTTTAGAT------------TTACAGTTTAA------AATGAATTAATGATTTTA--CAGGACAACGTCT-GTCGGGT---CCG----

>Bmor_Hel1Aa

------TATATATAATTCTTCTGTCCGTGTGTATGTTAGTGAACTCCTCCTAAACGGCTGGACTGATTTTGATGAAATTTT----TTGTGTGT-GTTCCAGTGGGTTC

GAGA------GTGGTTTAGAT------------TCACAATTNTA------ACTAATTCA-------TA--TAGGACAACATCT-GTCGGGT---CTG----

>Bmor_Hel1Ca

------TATATATATTTCTTCTGTGCGTGTGTTTGTCACTGAACTCCTCCTAAACGGCTGGACCGATTT-AATG--ATTTT----TTTTGTAT-GCTTTGGGTGGCCC

CTGG------ATGGTTTAGAT------------TCACAAATCA---------TGAGTCGGTATCTTTG--CAAAACAACGTTT-GCCGGGC---CAG----

>Bole_Hel1Aa

------ATTATATATTTCTTGTGTGCGTGTGTATGTCACTGAACTCCTCCTAGACGGCTGGACCGATTTTGATGAAATTTT----TTGTGTGA-GTTCAAGGGGATTC

GAGG------ATGGTTTAGAT------------T-AC-ATTTAA------AATGAACTAATAATTTTA--CAGGACAACGTCT-GTCGGGC---TTG----

>Btry_Hel1Ba

------TATATATAAATCTTCTGACCGTGTGTTTGTAATTGAACTGCTCCTAAACGGCTGGACCGATTTTGATGAAATTTT----TTGTGTGT-GTTTAAGGAGATTC

GAGG------ATGGTTTAGAT------------TCACAATTTAA------AATGAATTAATAATTTTG--CCACATGAAGCGT-GACGGGT---CCG----

>Cnem_Hel1Ea

------TATATATAATTCTTCTGTGAGTGTGTATGTCACTGAACTCCTTCTAGACGACTGGACCGATGTCGATGAAACTTT----TTGTATGT-GTTCTAGGGAATTC

GAGA------ATGGTTTAGAT------------TCACAATTTAA------AATGAATTATTGATTTTG--CGATACGAAGTTT-GCTGGGT---CAG----

>Csup_Hel1Aa

------TATATATAATTCTTCTGTGAGTGTGTATGTCACTGAACTTCTCTTAAACGACTGGACCGATTTTGATGAAATTTT----TTGTGTGT-GTTCAAGGGGATCT

GAGA------ATGGTTTAGAT------------TCACAA---------------------------A--CAGGACAACGTCT-GTCGGGT---CCG----

>Csup_Hel1Ab

------TATATATAATTCTTCTGTGAGTGTGTATGTCACTGAACTTCTCTTAAACGACTGGACCGATTTTGATGAAATTTT----TTGTGTGT-GTTCAAGGGGATCT

GAGA------ATGGTTTAGAT------------TCACAATTTAC------AATGAATTAATGATTTTA--CAGGACAACGTCT-GTCGGGT---CCG----

>Csup_Hel1Ea

------TATATATAATTCTTCTGTGAGTGTGTATGTCACTGAACTTCTCTTAAACGACTGGACCGATTTTGATGAAATTTT----TTGTGTGT-GTTCAAGGGGATCT

GAGA------ATGGTTTAGAT------------TCACAATTTAC------AATGAATTAATG--------CGGTACGAAGTTC-GCTGGGT---CAG----

>Cves_Hel1Aa

------TATATATATTTCTTGTGTGCGTGTGTATGTGACTGAACTCCTCCTAAACGACTGGACCGATTTTGATGAAATTTT----TTGTGTGT-GTTCAAGGGGATCT

GGGA------ATGGTTTAGAT------------TCACAATTTAC------AATGAATTAATGATTTTA--CAGGACAACGTCT-GTCGGGT---CCG----

>Cvic_Hel1Ca

------TATATATAATTCTTCTGTACGTGTGTTAGTAACTGAACTCCTCCTTAACGGCTGGGCCGATTTCGATGAAATTTG----TTGTGTGT-GTTTGAGTGGGTCC

CTGG------ATGGTTTAGAT------------TCACAATTGGA---------AAATGGGCTATTATG--CAGAACAACGTTT-GCCGGGA---CAG----

>Cvir_Hel1Ea

------TATATATAATTCTTGTGTTCGTGTGTTTGTCACTGAACTCCTTCTAGACGACTGGACCGATTTTGATGAAACTTT----TTGTGTGT-GTTCAAGGGAATTC

GAGA------ATGGTTTAGAT------------TCACAATTTAA------AATGAATTATTGATTTTG--CGATACGAAGTTT-GCTGGGT---CAG----

>Daus_Hel1Aa

------TATATATAATTCTTCTGTGCGTGTGTATGTCACTGAACTCCTCCTAAACGGCTGGACCGATTTTGATGAAATTTT----TTGTGTGT-GTTCAAGTGGATTC

GAGA------ATGGTTTAGAT------------TCACAATTCAA------AATGAATAAATGATCTTA--CAGGACAACGTCT-GTCGGGT---CCG----

>Hvit_Hel1Ga

------TATATATATTTCTTGTGTTCGTGTGTATGTGACTGAACTCCTCCTAAACGACTGGACCGATTTTGATGAAATTTT----GTGTGTGT-TTTCAATGGAATTC

GAGA------ATGGTTTAGAT------------TCACAATTTAA------ATTGAATTAATGATTTTA--CAGGAAATTGCGT-GCGAAGC---CGG----

>Lmig_Hel1Aa

-------ATATATATTTCTTCTGTCCGTGTGTTTGTCACTAAACTCTCCCTAAACGGCTGGATCGATTTACATGAAATTTT----TTGTGTGT-GTTTAAGGGGATTC

GAGA------ATGGTTTAGAT------------TCACATTTTAA------AATGAATTAATGATTTTA--CAGGACAACGTCT-GTCGGGT---CCG----

>Mcin_Hel1Ca

------TATATATATTTCTTGTGTGCGTGTGTATGTCACTGAACTCCTCCTAGACGGCTGGACGGATTTTGATGAAATTTT----TTGTGTGA-GTTCAAGGGGATTC

GAGG------ATGGTTTAGAT------------TCACAATTTAA------AATGAACTAATAATTTTG--CAAAACAACGTTT-GCGGGGT---CAG----

>Mcon_Hel1Aa

------TATATATATTTCTTGTGTACGTGTGTATGTCACTGAACTCCTCCTAAACGGCTGGACCGATGTTGATGAAATTTT----TTGTGTGT-GTTCAAGGGGATTC

GAGA------ATGGTTTAGAT------------TCACAATTGGT------GGCGAATCGATAATTTTA--CAGGACAACGTCT-GTCGGGA---CCG----

>Mper_Hel1Fa

------TATATATAATTCTACTGTACGTGTGTATGTCACTGAACTCCTCCTAAACGGCTGGACCGATTTGAATGAATTTTT----TTGTATGC-GTTTGGGTGACGCC

CTGG------ATGGTTTAGAT------------TCACAATTGAC------AGGGAAATATTGGTTATC--GGGCACAAAGTGC-ACGGGAT---CAG----

>Ncla_Hel1Aa

------TATATGTAATTCTTCTGTGCGTGTGTATGTAACTGAACTCCTCCTAAACGGCTGGACCGATTTTGATGAAAGTTT----TTGTCT---GTTCCAGTGGACTC

GAGA------ATGGTTTAGAT------------TTATAATTCGA------AATGAATTAATGATCTTA--CAGGACAACGTCT-GTCGGGT---CCG----

>Obru_Hel1Ia

-------TTATATAATTCTTCTGTACGTGTGTATGTCACTGAACTCCTCCTAAACGGCTGGACCGATTTTGATG---TTTT----TTTTGTGT-GTTTGAGTGGGTCC

CTGG------ATGGTTTAGAT------------TCACAACTGGT------GGCGAGTC--------------GGGCGAAGTCG-AGCGGG----CCG----

>Pdom_Hel1Aa

------TATATATAATTCTTCTGTGCGTGTGTATGTCACTGAACTCCTCCTAAACGGCTGGACCGATTTTAATGAAATTTT----TTGTGTGT-CTTCAGGTGGATTC

GADA------ATDGTTTAGAT------------TTACAATTCAA------ATTGAATTAATTTTTGTA--BAGGACAACGTCT-GTCGGGT---CCG----

>Pgla_Hel1Ga

------TATATATATTTCTTGTGTGCGTGTGTATGTCACTGAACTCCTCCTAGACGGCTGGACCGATTTTGATGAAATTTT----TTGTGTGA-GTTCAAGGGGATTC

GAGG------ATGGTTTAGAT------------TCACAATATAT-----------------------------AAGAGTGCGT-GCGAAGC---CGG----

>Pgra_Hel1Aa

------TATATATATTTCTTGTGTGCGTGTGTATGTCACTGAACTCCTCCTAAACGACTGGACCGATTTTGATGAAATTTT----TTGTGTGT-GTTCAAGGGGATTT

GAGA------ATGGTTTAGAT------------TCACAATTTAA------AATGAATTAATGGATTAA--CAGGACAACGTCT-GTCGGGT---CCG----

>Pmac_Hel1Ha

------TATATATATTTCTTGTGTGCGTGTGTATGTGACTGAACTCCTCCTAAACGACTGGACCAATTTTGATGAAATTTT----TTGTGTGT-GTTCGTGGAGATTC

GAGA------ATGGTTTAGAT------------TTACAGTTTAA---------------------TTC--CGCGCGGACG-------GAGT---CGG----

>Prap_Hel1Aa

------TATATATATTTCTTGTGTGCGTGTGTATGTGACTGAACTCCTCCTAAACGACTGGACCGATTTAGACGAAATTTT----TTGTGTGT-GTTCAAGGGGATCT

GGGA------ATGGTTTAGAT------------TCACAATTTAC------AATGAATTAATGATTTTA--CAGGACAACGTCT-GTCGGAT---CCG----

>Ptep_Hel2Ca

------TATATATAAATCTCTTGTCACAGTTTTAGTGTTTAAACTCCTCCGAAACGGCTTGACCGATTTTGATGAAATTTT----ATATGTAT-ATTC-AGTAGGTCT

GAGA------ATGGTTTATAT------------TCATTTTTTAA--------CGAAATATACACGTTG--CAAAACAACGTTT-GCCGGGT---CAG----

>Pxut_Hel1Aa

------TATATATAATTCTTCTGTGAGTGTGTATGTCACTGAACTTCTCTCAAACGACTGGACCGATTTTGATGAAATTTT----TTGTGTGT-GTTCAAGGGGATCT

GGGA------ATGGTTTAGAT------------TCACAATTTAC------AATGAATTAATGATTTTA--CAGGACAACGTCT-GTCGGGT---CCG----

>Pxut_Hel1Ab

------TATATATAATTCTTCTGTGAGTGTGTATGTCACTGAACTTCTCTCAAACGACTGGACCGATTTTGATGAAATTTT----TTGTGTGT-GTTCAAGGGGATCT

GGGA------ATGGTTTAGAT------------TCACAATT-------------ATCAAATAATTTAA--CAGGACAACGTCT-GTCGGGT---CCG----

>Pvar_Hel1Da

------TATATATATTTCTTCTGTGCGTGTGTTTGTCACTGAACTCCTCCTAAACGGCTGGACCGATTTTGATGAAATTTT----TTGTGTAT-GTTCAAGGGGATTC

GAGA------ATGGTTTAGAT------------TCACAATTTAA------AAAGAATTAATAATTTCA--TAGAATAAGGGCT-GCCGGGT---CAG----

>Sfru_Hel1Aa

------TATATATAATTCTTCTGTAAGTGTGTATGTCACTGAACTTCTCTTAAACGACTGGACCGATTTTGATGAAATTTT----TTGTGTGT-GTTCAAGGGGATCT

GAGA------ATGGTTTAGAT------------TCACAATTTAC------AATGAATTAA------TA--CAGGACAACGTCT-GTCGGGT---CCG----

>Sfru_Hel1Ab

------TATATATAATTCTTCTGTAAGTGTGTATGTCACTGAACTTCTCTTAAACGACTGGACCGATTTTGATGAAATTTT----TTGTGTGT-GTTCAAGGGGATCT

GAGA------ATGGTTTAGAT------------TCACAATTTAC------AATGAATTAATGATTTTA--CAGGACAACGTCT-GTCGGGT---CCG----

>Sfru_Hel1Ac

------TATATATAATTCTTCTGTAAGTGTGTATGTCACTGAACTTCTCTTAAACGACTGGACCGATTTTGATGAAATTTT----TTGTGTGT-GTTCAAGGGGATCT

GAGA------ATGGTTTAGAT------------TCACAATTTAC------AATGAATTAATGATTTTA--CAGGACAACGTCT-GTCGGGT---CCG----

>Tcri_Hel1Aa

------TATATATAATTCTTCTGTACGTGTGTATGTCACTGAACTCCTCCTAAACGGCTGGACCGATTTTGATGAAATTTT----TTGTGTGT-GTTTGTGTGGGTCC

ATGG------ATGGTTTTGAT------------TCACAATTGGA------TTTAAATTTATGATTTCG--CAGGACAACGTCT-GTCGGGT---CCG----
